# Supplementary material for: Prematurity detection evaluating interaction between the skin of the newborn and light: protocol for the preemie-test multicentre clinical trial in Brazilian hospitals to validate a new medical device
Source: BMJ Open. 2019 Mar 5;9(3):e027442. doi: 10.1136/bmjopen-2018-027442 (PMC6429838; doi:10.1136/bmjopen-2018-027442)
Supplement: Supplementary file 1 [file bmjopen-2018-027442supp001.pdf]

TERMO DE CONSENTIMENTO LIVRE E ESCLARECIDO

Detecção da prematuridade através da interação entre a luz e a pele neonatal: a validação do Preemie-Teste

Sob responsabilidade da pesquisadora Profa Zilma Silveira Nogueira Reis

Cara senhora, você está sendo convidada a participar deste estudo porque acaba de ter um parto no hospital (nome do hospital do centro colaborador) ____.

Apresentação do estudo

O objetivo deste estudo é descobrir novas técnicas para estimar a idade de um bebê ao nascer e identificar aqueles que nasceram antes de nove meses, os prematuros. A idade gestacional desconhecida pode aumentar o risco dos bebês no momento de seu nascimento. As técnicas atuais para se estimar a idade do bebê possuem grande margem de erro.

Acreditamos que a pele possui características que, se bem estudadas, podem refletir a idade das pessoas, e também dos bebês. Por isso, estamos desenvolvendo um novo equipamento médico que se encontra em teste. Ele utiliza a luz para avaliar a composição da pele do bebê e detectar sua idade. Os resultados poderão beneficiar os bebês que nascem sem a informação confiável da idade gestacional.

Instituições envolvidas no estudo

O estudo é desenvolvido pela Faculdade de Medicina da Universidade Federal de Minas Gerais (UFMG), em cooperação com maternidades brasileiras, entre elas a que você se encontra internada. A previsão deste estudo é que 787 crianças recém-nascidas sejam examinadas.

A participação no estudo, riscos e cuidados

Convidamos você e seu bebê para participar deste estudo. Isso incluirá um exame na pele do bebê com a luz, uma breve entrevista com você e a consulta aos registros de saúde sobre a gravidez e os do seu bebê neste hospital. Na entrevista serão tomados todos os cuidados a fim de minimizar os constrangimentos para você. A consulta ao prontuário médico será realizada resguardando o direito de sigilo da informação. Pedimos sua permissão para fotografar a caderneta da gestante ou outro documento equivalente, para conferir a idade gestacional calculada pelos ciclos menstruais e pelos exames de ultrassom. As partes da fotografia que contenham sua identificação serão retiradas da imagem e a manteremos até o final do estudo, quando o arquivo será apagado dos registros da pesquisa.

Pedimos sua permissão para fazer um exame na pele de seu bebê, na região da sola do pé, usando um equipamento em teste. O exame é indolor e externo ao corpo, considerado não-invasivo. A parte que encosta no bebê é pequena e não apresenta pontas que possam ferir a sua pele. Outros equipamentos parecidos, que emitem luz, já são usados nos bebês de forma segura. Por exemplo o oxímetro que faz teste do coraçãozinho. Assim como esse, não se espera que ocorram efeitos imediatos ou futuros na saúde do bebê. Os riscos do teste que faremos incluem a exposição do pé do bebê com perda temporária de calor do corpo e estresse. Cuidados serão tomados a fim de minimizar estes desconfortos. Esclarecemos que o teste dura alguns segundos reduzindo ao mínimo chance de causar marcas ou irritação no local. Caso seu bebê apresente sinais de desconforto durante o exame, o mesmo será interrompido. Você ou familiares poderão permanecer junto ao seu filho durante o exame. Nas crianças que estiverem na Unidade Neonatal, o exame será realizado onde ela já está sendo cuidada, acompanhado pelo profissional de saúde que já está cuidando dela. Caso o seu bebê seja prematuro, todos os devidos cuidados serão tomados antes de cada exame para reduzir a chance de perda de calor, seguindo todas as recomendações de um bebê que fica em incubadora.

Esclarecemos que este estudo não trará benefícios diretos a você ou seu filho, entretanto auxiliará na validação de um novo teste que poderá no futuro identificar o bebê prematuro. Os resultados poderão também gerar informações que ajudem a melhorar os cuidados com outros bebês, quando a idade gestacional é desconhecida. Informamos que os resultados da pesquisa serão publicados em revistas científicas e apresentados em congressos, sem contudo revelar sua identidade ou a do bebê.

As informações obtidas durante a pesquisa serão confidenciais, guardadas em computadores, protegidos por senha e não serão usadas para outros fins. O roubo das informações que coletaremos no estudo é um risco remoto. Para isso, as melhores práticas em segurança de dados serão empregadas. Também poderão ter acesso aos dados da pesquisa o comitê que coordena o estudo, assim como a agência reguladora ANVISA, sem jamais violar a confidencialidade e privacidade dos dados, para que seja possível monitorar se os procedimentos de qualidade e segurança da pesquisa estão sendo seguidos.

Seus direitos como participante

Informamos que a sua participação deve ser voluntária, ou seja, não é obrigatória e caso não concorde ou resolva desistir a qualquer momento isto não trará nenhum constrangimento para você ou para a forma como você será tratada neste hospital. Também não está previsto nenhum tipo de pagamento por sua participação na pesquisa. Este estudo não implica em gastos para você, pois não terá que se deslocar para outro local, permanecer mais tempo no hospital, uma vez que o exame é feito durante sua internação e de seu bebê na maternidade. Caso seja de seu interesse, os resultados do exame que estarão guardados com o pesquisador e lhe serão entregues assim que você solicitar.

Os pesquisadores garantem que acompanharão gratuitamente seu bebê durante a realização do exame e a qualquer momento que se fizer necessário, em qualquer problema que por ventura esteja associado ao estudo ou efeito do teste com a luz.

Este Termo de Consentimento está elaborado em duas vias iguais. Ambas devem ser assinadas por você, pelo pai da criança e pelo pesquisador. Uma via ficará com o participante e a outra com o pesquisador.

O Comitê de Ética em Pesquisa da UFMG pode ser contatado em caso de haver dúvidas quanto aos aspectos éticos da pesquisa, através do telefone (31) 3409-4592 ou endereço completo apresentado a seguir.

Meu nome	
Documento de identidade	
Data de hoje	

Eu declaro que estou em condições de tomar esta decisão e ciente do que foi exposto acima. Autorizo o uso de minhas informações de saúde e as do meu bebê para este projeto de pesquisa, assim como a realização do novo teste. Participo voluntariamente deste estudo e estou ciente que o exame na pele do meu bebê com a luz não traz prejuízo à sua saúde

Assinatura da puérpera:
Assinatura do pai da criança:
Assinatura do pesquisador:

Telefones de contato:
Maternidade Hospital das Clínicas da UFMG – (31) 34099422
Hospital (nome e telefone do hospital colaborador)
Zilma Reis – (31) 985177473 e-mail: skinage.ufmg@gmail.com
Comitê de Ética em Pesquisa da UFMG – Av. Prof. Antônio Carlos, 6627, Unidade Administrativa II, 2° andar, sala 2005, Campus Pampulha, CEP: 31270-901. E-mail:coep@prpq.ufmg.br. Fone (31) 34094592.
Comitê de Ética em Pesquisa do centro colaborador e endereço completo, com e-mail.
