# Supplementary material for: Prematurity detection evaluating interaction between the skin of the newborn and light: protocol for the preemie-test multicentre clinical trial in Brazilian hospitals to validate a new medical device
Source: BMJ Open. 2019 Mar 5;9(3):e027442. doi: 10.1136/bmjopen-2018-027442 (PMC6429838; doi:10.1136/bmjopen-2018-027442)
Supplement: Author's manuscript [file bmjopen-2018-027442.draft_revisions.pdf]

BMJ Open is committed to open peer review. As part of this commitment we make the peer review history of every article we publish publicly available.

When an article is published we post the peer reviewers' comments and the authors' responses online. We also post the versions of the paper that were used during peer review. These are the versions that the peer review comments apply to.

The versions of the paper that follow are the versions that were submitted during the peer review process. They are not the versions of record or the final published versions. They should not be cited or distributed as the published version of this manuscript.

BMJ Open is an open access journal and the full, final, typeset and author-corrected version of record of the manuscript is available on our site with no access controls, subscription charges or pay-per-view fees (http://bmjopen.bmj.com).

If you have any questions on BMJ Open's open peer review process please email editorial.bmjopen@bmj.com

BMJ Open

Prematurity detection evaluating interaction between newborn skin and light: The Preemie-Test Multicenter Clinical Trial to validate a new medical device

Journal:	*BMJ Open*
Manuscript ID	bmjopen-2018-027442
Article Type:	Protocol
Date Submitted by the Author:	24-Oct-2018
Complete List of Authors:	Reis, Zilma; Universidade Federal de Minas Gerais, Gynecology and Obstetrics<br>Guimarães, Rodney; Faculty of Medicine, Universidade Federal de Minas Gerais, Ginecologia e Obstetrícia<br>Rego, Maria Albertina; Faculty of Medicine, Universidade Federal de Minas Gerais, Pediatrics<br>Maia de Castro Romanelli , Roberta ; Universidade Federal de Minas Gerais Faculdade de Medicina,<br>Gaspar, Juliano; Faculty of Medicine, Universidade Federal de Minas Gerais, Obstetrics and Gynecology<br>Vitral, Gabriela; Faculty of Medicine, Universidade Federal de Minas Gerais<br>dos Reis, Marconi; Faculty of Medicine, Universidade Federal de Minas Gerais, Pediatrics<br>Colósimo, Enrico; Universidade Federal de Minas Gerais, Statistics<br>Neves, Gabriela; Hospital Sofia Feldman<br>Vale, Marynea; Hospital Universitario da Universidade Federal do Maranhao, Pediatrics<br>Nader, Paulo; Universidade Luterana do Brasil. Hospital Universitário de Canoas<br>de Moura, Martha; Hospital Materno Infantil de Brasília<br>de Aguiar, Regina ; Faculty of Medicine, Universidade Federal de Minas Gerais, Obstetrics and Gynecology
Keywords:	Gestational Age, Infant, Premature, Skin Physiological Phenomena, Photomedicine, Equipment and Supplies

SCHOLARONE™
Manuscripts

Prematurity detection evaluating interaction between newborn skin and light: The Preemie-Test Multicenter Clinical Trial to validate a new medical device

Zilma Silveira Nogueira Reis[1]*; Rodney Nascimento Guimarães[1]; Maria Albertina Santiago Rego[1]; Roberta Maia de Castro Romanelli[1]; Juliano de Souza Gaspar[1]; Gabriela Luíza Nogueira Vitral[1]; Marconi Augusto Aguiar dos Reis[1]; Enrico Antônio Colósimo[2]; Gabriela Silveira Neves[3]; Marynea do Vale Nunes[4]; Paulo de Jesus Hartmann Nader[5]; Martha David Rocha de Moura[6]; Regina Amélia Pessoa Lopes de Aguiar[1]

1 - Faculty of Medicine, Universidade Federal de Minas Gerais, Brazil.

2 - Statistics Department, Universidade Federal de Minas Gerais, Brazil.

3 - Hospital Sofia Feldman, Brazil.

4 - Hospital Universitário da Universidade Federal do Maranhão, Brazil.

5 - Universidade Luterana do Brasil. Hospital Universitário de Canoas, Brazil.

6 - Hospital Materno Infantil de Brasília, Brazil.

*Corresponding author: Zilma Silveira Nogueira Reis. Avenida Professor Alfredo Balena, 190, sala 601. Zip Code 30.130.100 Belo Horizonte, Brazil. Phone: +55 0 31 3409-9467. Email: zilma@medicina.ufmg.br.

For peer review only - http://bmjopen.bmj.com/site/about/guidelines.xhtml

Abstract

Introduction: Recognizing prematurity is critical in order to attend to immediate needs in childbirth settings, guiding the extent of medical care provided for newborns. A new medical device has been developed to carry out the Preemie-Test, an innovative approach to estimate gestational age (GA), based on the photobiological properties of the newborn's skin. This study will validate the Preemie-Test for GA estimation at birth and its accuracy to detect prematurity. Secondarily, the study intends to associate the infant´s skin reflectance with lung maturity, as well as evaluate safety, precision, and usability of a new medical device to offer a suitable product for health professionals during childbirth and in neonatal care settings.

Methods and analysis: Research protocol for diagnosis, single-group, single-blinding, and single-arm multicenter clinical trials with a reference standard. Alive newborns, with 24 weeks or more of pregnancy age, will be enrolled during the first 24 hours of life. Sample size is 787 subjects. The primary outcome is the difference between the GA calculated by the photobiological neonatal skin assessment methodology and the GA calculated by the comparator antenatal ultrasound or reliable last menstrual period. Immediate complications caused by pulmonary immaturity during the first 72 hours of life will be associated with skin reflectance in a nested case-control study.

Ethics and dissemination: Each local independent ethics review board approved the trial protocol. The authors intend to share the minimal anonymized data set necessary to replicate study findings.

Trial registration number: U1111-1205-0539; WHO Clinical Trial http://apps.who.int/trialsearch/Trial2.aspx?TrialID=RBR-3f5bm5.

For peer review only - http://bmjopen.bmj.com/site/about/guidelines.xhtml

Key-words: Gestational Age, Infant, Premature; Skin Physiological Phenomena; Photomedicine; Equipment and Supplies.

Article Summary

Strengths and limitations of this study:

- The study has the potential to validate a new approach for pregnancy dating.
- The device will be subjected to high-quality clinical study to demonstrate benefits.
- The gold standard comparator for pregnancy dating does not exist, instead a reference standard will be used with blinded primary outcome.
- The agreement endpoint between methods for gestational age determination precluded randomization of the intervention.

For peer review only

For peer review only - http://bmjopen.bmj.com/site/about/guidelines.xhtml

Introduction

In childbirth settings, health professionals continuously need to make timely decisions to provide proper neonatal care. The day of birth is the riskiest for newborns and mothers almost everywhere[1]. Perinatal causes related to prematurity and complications during childbirth, which are generally preventable through qualified health care, are the primary causes of death among newborns[1,2]. Most of these deaths took place in countries with low resources and a scarcity of health facilities[3]. The opportune recognition of prematurity is critical in order to judge the viability of the newborn and to attend to his/her immediate needs, guiding the complexity of the medical care provided for the newborn. Without reliable information on the age of the unborn phase, actions to preserve the potential for survival of the newborn can be neglected[4]. Indeed, the attempted management of the risk of mortality and severe complications are sensitive issues to the gestational age (GA), which involves temperature maintenance, ventilatory support, transport to a neonatal intensive care unit (NICU), and the early treatment of respiratory distress syndrome (RDS), the most severe complication of premature birth[5]. In addition to the GA information or birthweight, the prediction of neonatal respiratory morbidity may be critical in planning immediate medical care [6], since the respiratory system is among the last of the fetal organ systems to mature, which is associated with enhanced morbidity and mortality[6].

Current methods of dating pregnancy remain a worldwide challenge. Early obstetric ultrasound currently offers the best due date[7]. However, access to this type of exam is limited because of high equipment costs, poor training and skills of health professionals, or late prenatal care[8]. Despite a 10-days or more margin of error during the second and third trimester of gestation, ultrasound is still a reasonable methodology for GA determination, when the best opportunity was lost[7]. The calculation, based on the historical information of

For peer review only - http://bmjopen.bmj.com/site/about/guidelines.xhtml

the last menstrual period (LMP), is impacted by the uncertainty of both the fertility days and date of conception[9], due to the bias of memory, the use of hormonal contraception, and breastfeeding[10]. After birth, neurological scores, such as the New Ballard[11], show a tendency to overestimate GA in preterm infants and underestimate GA in growth-restricted infants[12]. Efforts to enhance the reliability of pregnancy dating, through more accurate and accessible technologies, seek to improve pregnancy outcomes and neonatal survival[13].

A new medical device has been developed to carry out the Preemie-Test, an innovative approach used to estimate GA, based on the photobiological properties of the newborn's skin. This reflective test is noninvasive, and the device automatically processes the light, scattered by the constituents of the skin layers, when a small optoelectronic light emitter/receiver sensor touches the newborn's skin[14]. the device under test is easy to use and every effort is being made to ensure that it has excellent accuracy, be it safe and low cost. The feasibility study provides a mathematical model to predict GA based on the skin reflectance adjusted to clinical variables (R^2 = 0.828, P <0.001)[15]. However, before the adoption or use of an innovation, an effectiveness trial of intervention is a critical step in the research chain regarding its the social utility when completing the translation from the proof of concept to clinical science [16]. The rationale for the main hypothesis in this study is that the skin maturity of a newborn, obtained by the analysis of its optical properties, is useful in pregnancy dating for clinical use and respiratory prognosis, especially in a scenario with no reliable GA based on current methods. This study aims to validate the photobiological model of the skin, called the “Preemie-test”, in order to estimate GA at birth and determine its accuracy in detecting prematurity. Secondarily, it also seeks to associate the infant’s skin reflectance with lung maturity. Moreover, this study intends to evaluate the safety, precision,

For peer review only - http://bmjopen.bmj.com/site/about/guidelines.xhtml

and the usability of a new medical device to offer a suitable product to support health professionals during childbirth and in neonatal care settings.

Methods

Study design

This study will use a protocol for diagnosis, single-group, single-blinding, and single-arm multicenter clinical trials with a reference standard. This new photobiological approach to the skin, gathered in a medical device, is currently in the pivotal phase of innovation development from the prototype to regulatory approval [17]. This step aims to provide the translation[16] of the scientific model for GA detection based on skin maturity. This Protocol version is 1, July/10th/2018. Faculty of Medicine, Universidade Federal de Minas Gerais is the Coordinator Center.

Study Settings, Ethics and Dissemination

Selected Brazilian referral centers for high-risk pregnancy and neonatal care will participate in the study, according to this protocol: Hospital das Clínicas, Universidade Federal de Minas Gerais, as the Center for Coordination; Hospital Sofia Feldman, Minas Gerais State; Hospital da Universidade Luterana do Brasil, Rio Grande do Sul State; Hospital Materno-infantil de Brasília, Distrito Federal; and Hospital Universitário da Universidade Federal do Maranhão, Maranhão State. Each local independent ethics review board approved the trial protocol, and the Brazilian National Research Council (CONEP) approved all study activities and protocol prior to the commencement of study activities, in accordance with the Declaration of Helsinki (2008), good clinical practice as set forth by the International Organization for Standardization (ISO) 14155:2011, and the Brazilian regulatory health agency's recommendations[18]. This study was logged under both protocol number CAAE

For peer review only - http://bmjopen.bmj.com/site/about/guidelines.xhtml

81347817.6.1001.5149 and the International Clinical Trials Registry Platform under Universal Trial Number U1111-1205-0539 is accessible by http://apps.who.int/trialsearch/Trial2.aspx?TrialID=RBR-3f5bm5. Parents will sign an informed consent form on behalf of the newborn before participating in the clinical trial (supplementary file).

Data Sharing Statement

The authors intend to share the minimal anonymized data set necessary to replicate study findings. Data sharing will include: the reference and comparators GA, GA estimated by the Preemie-test, birth weight, RDS or transient tachypnea of the newborn (TTN) diagnosis, ventilatory support due to pulmonary immaturity, neonatal intensive care unit (NICU) admission due to RDS or TTN, and any adverse events regarding device's safety. Unidentified data and study-related documents as ethical approvals will be accessible by URLs for researchers, regulatory agencies, and sponsors.

Patient and Public Involvement

Patients and the public were not involved in the design of this study. The results will be disseminated to study parents of participants through scientific publications, non-scientific publications, and on the website of the project: http://skinage.medicina.ufmg.br.

Eligibility criteria and participant's timeline

A prospective sequential and concurrent enrollment process will select newborns in referral hospitals centers for neonatal care. Infants are eligible with the following inclusion criteria: (1) alive newborn; (2) enrollment during first 24 hours of life; (3) be 24 weeks or more of gestational age, at birth; (4) fetus underwent an obstetric ultrasound assessment before 14 weeks of pregnancy; (5) fetus also had obstetric ultrasound assessment between 14 and 22

For peer review only - http://bmjopen.bmj.com/site/about/guidelines.xhtml

gestational weeks. Exclusion criteria are: (1) malformation with structural skin alterations; (2) skin modifiers: anhydramnios, hydrops, congenital skin diseases or chorioamnionitis. Randomisation was not appropriate to assess the agreement between different methods to assess pregnancy dating.

In a nested case-control study, we will select newborns within the first 72 hours of life, discharge, or death, whichever occurs first, with the following inclusion criteria: (1) RDS or (2) TTN diagnosis. Ranges of gestational age will randomly pair controls. Exclusion criteria include: (1) the existence of extra pulmonary conditions with tachypnea not due to prematurity and (2) diagnosis of Clinical or Laboratory-Confirmed Bloodstream Infection.

Intervention: The Preemie-Test

The Preemie-Test assessment occurs as soon as possible after birth, in the first 24 hours, inside incubators, open heating crib, common crib or in the mother's lap, in order to ensure minimum manipulation and stable clinical conditions. The acquisitions of all newborns will be stored in a database for further statistical analysis.

A noninvasive, handheld optoelectronic prototype has been developed to measure the backscattered light signal from the skin[15]. The equipment regulates the emitted light and processes the received light signal in the sensor, resulting in the prediction of GA by a mathematical model, associated or not with clinical variables. According to the Brazilian regulatory health agency (ANVISA), this medical device is categorized as a Class II safety: noninvasive and medium risk. The prototype unit of measurement and the process of GA estimation were patented under number BR1020170235688 (CTIT-PN862)[14]. An updated version of the invention received improvements in order to safeguard reliability and to minimize examiner interferences on the skin's backscattering acquisition. The light emitting-sensor touches the skin over the sole of the foot for a few seconds. The skin reflectance will

For peer review only - http://bmjopen.bmj.com/site/about/guidelines.xhtml

be sensed once the light has been emitted by a light emitting diode (LED) at wavelengths from 400 nm to 1200 nm. Data acquisitions occur automatically, without operator influence, and are obtained three times per newborn, in the same site and sequentially. Digital recordings will be uploaded to a server for further analysis. The prototype will blind the examiner to the predicted GA value.

The criterium for discontinuing the interventions for a given trial participant will be in case of parents of the newborns' request.

Training and monitoring

Systematic monitoring of data collection, through an electronic information system, would trigger any adverse event. This medical team is still responsible for the training of healthcare professionals to recruit participants, data collection, a safely performed Preemie-Test during the newborn's assessment, and the monitoring of data quality. The certification of co-participant centers involved the accomplishment of at least 30 simulated examinations by the participant health professionals in the study.

Gestational age methods of calculation and comparators

Reference-GA (R) is calculated upon enrollment, using the embryo measurement assessed by ultrasound exam at <14 weeks of gestation as a reference. Crown-rump-length (CRL) data, recorded from the ultrasound report or prenatal care book-document, will be considered the crude data, when available. Intergrowth’s 21st standard curve for ultrasound measurements from 7 weeks and 3 days up to 13 weeks and 6 days will be adjusted to all GA data, according to CRL[19].

GA methods to calculate GA in the childbirth setting, and their comparators are as follows:

For peer review only - http://bmjopen.bmj.com/site/about/guidelines.xhtml

- Preemie-Test-GA (T): data statistically determined by analyzing the acquired information stored in the device's processor.
- Comparators-GA (C): calculated using the first ultrasound exam after 13 weeks and 6 days of gestation and before 22 weeks (C1). When available, a second comparator is GA based on a reliable LMP (C2)[13].

We will take a scanning copy of the prenatal care book or the ultrasound report. After evaluating the data quality, the images will be discarded. To achieve a reliable LMP, we will interview the woman, as suggested by Nguyen et al. (2000)[13].

Primary outcome measures

The primary target is the agreement between the GA offered by the Preemie-Test (T) and the GA calculated by the comparators (C1 and C2), so as to perform the new test in scenarios without the Reference-GA (R). The outcome is the difference between the GA calculated by the photobiological neonatal skin assessment methodology in relation to the age calculated by the comparators.

Another measure for the primary target is the detection of preterm newborns, considering the age before 37 weeks of pregnancy as the threshold between term and preterm births, and analyzing sub-categories of preterm birth, based on GA[4]:

- extremely preterm (less than 28 weeks)
- very preterm (28 to 32 weeks)
- moderate to late preterm (more than 32 to less than 37 weeks).

In this case, the outcome is the proportion of the preterm newborn correctly detected at birth, based on the photobiological test of the skin, within a one-week error.

For peer review only - http://bmjopen.bmj.com/site/about/guidelines.xhtml

Secondary outcome measures

1. In a simulated scenario, in which the Reference-GA (R) is unknown, two groups will be randomly assigned from the complete database in order to compare differences among the Reference-GA (R), the GA obtained through the Preemie-Test (T), and the GA calculated by the comparators. Figure 1 presents such subgroups and measures for comparison.
2. To monitor the device's safety when in regular use by participants over a 72-hour period. Adverse events will be monitored, according to ISO 14155:2011 standards. This means any unexpected medical events, unintended disease or injury, or unfortunate clinical signs in subjects, users, or other people, whether related to the investigational medical device or not.
3. To establish the *ease of use* of the Preemie-Test measurement as a potential method for preterm newborn diagnosis.

The secondary outcome measures in the case-control nested study

Immediate complications, occurring during the first 72 hours of life due to pulmonary immaturity, are the secondary target. The outcome measures are as follows:

- To describe the relationship of the measurement of the newborn's skin reflectance with RDS and with diagnoses based on clinical and radiological findings and respiratory outcomes[6,20].
- To describe the relationship of the measurement of the newborn's skin reflectance with the TTN and with diagnoses based on clinical findings and respiratory outcomes[6].

For peer review only - http://bmjopen.bmj.com/site/about/guidelines.xhtml

For peer review only

- To describe the relationship of the measurement of the newborn's skin reflectance with ventilatory support due to pulmonary immaturity.
- To describe the relationship of the measurement of the newborn's skin reflectance with NICU admission due to RDS or TTN.

Time schedule of enrollment, intervention, and outcome measurements are presented in a schematic diagram (see Figure 2). The assessment occurs during the first 24 hours of life, but participants will be followed up for 72 hours or until discharge or death, whichever occurs first, for the monitoring of neonatal outcomes and adverse events.

Sampling and sample size

The sample size calculation is estimated based on the primary endpoint. To test the hypothesis of equivalence between the Preemie-Test GA and the comparators GA, a sample of 787 subjects is necessary to detect an effect size of 10%. Using the G-Power 3.1 software[21], we assumed an alpha error of 0.05, and a power of test of 0.80 to support a paired t-test.

Sampling intends to arrange three groups of GA enrollment to preserve enough premature newborns with 3:2:1 proportion, similar to Wilson et al. (2017)[22]: 392 term newborns, 263 premature newborns from 32 to 36 weeks and six days of GA, and 132 extremely premature newborns from 24 to 31 weeks and six days of GA.

Usability

The usability assessment will be performed by applying a checklist to participants who use the prototype device to perform the Preemie-Test. The 10 heuristics proposed by Nielsen and Marck |(1994)[23] will be adapted to build a checklist to evaluate the device, namely: (a) system visibility, (b) correspondence with the real world, (c) user control and freedom, (d)

For peer review only - http://bmjopen.bmj.com/site/about/guidelines.xhtml

consistency of results and standardization, (e) error prevention, (f) visual recognition rather than memorization, (g) flexibility and efficiency of use, (h) esthetic and minimalist design, (i) help for the user to recognize, diagnose, and recover from errors, and (j) user documentation and help.

Data collection

Standard operational procedures set data entries in structured questionaries. In this concurrent clinical trial, an electronic information system was developed to collect data in different hospitals, simultaneously. Entry forms validations were implemented with data values ranges to ensure the quality of the information. An audit of the data will be permanently performed and the data summary available on the project webpage. Double system, paper-based and electronic will permit audit concerning reliability and validity. Independent rater over-read all papers files and cross check with the electronic information from all patients.

Data analysis

Demographics and baseline characteristics of the study group, as well the intervention measurements, will be summarized by the frequencies and the mean and standard deviation (SD), the whereas median and interquartile range will be preferred for non-normally distributed continuous variables.

To model the GA prediction, computational randomization will select two subsamples in the database. One of them to train the prediction model of GA based on skin reflectance and clinical variables, such as sex, time in an incubator, phototherapy, birth weight, among others. Another part will be for the analytical validation of the predictive model.

For peer review only - http://bmjopen.bmj.com/site/about/guidelines.xhtml

Improvements in the existing prediction models for GA (Preemie-Test), will be conducted with conventional statistical and data mining analyses.

Regarding the primary endpoint, the agreement among three methods for GA will be calculated using the Intraclass coefficient correlation and Bland & Altman plots[24], and paired t-testing. The accuracy of the Preemie-Test in identifying the premature newborn, within a one-week margin of error, will be the target of the accuracy analysis.

The relationship between the measurement of the newborn's skin reflectance and complications due to pulmonary distress associated with immaturity will be evaluated by means of association tests and risk. The significance level for hypothesis tests will be 5%, together with 95% confidence intervals.

Results

The study begun with the training of health professionals in September 2018. It is anticipated that the recruitment will take place from January to December 2019. Data analysis will be finalized, the results of which are expected in May 2020.

Discussion

Strengths and Limitations

Availability of trustworthy GA information is a prerequisite for preterm birth classification and healthcare decisions[25]. In this light, the results of this clinical study have the potential to validate a new device for pregnancy dating. The Preemie-Test was prepared to operate with

For peer review only - http://bmjopen.bmj.com/site/about/guidelines.xhtml

minimum operator intervention and for use by healthcare professionals anywhere a birth takes place without a reliable GA.

The purpose of medical research involving neonates is intended to improve clinical procedures[26]. In this context, a clinical trial is a research study in which subjects are prospectively assigned to intervention and the effects of those interventions on health-related outcomes are thereby evaluated[27]. However, clinical trials on medical devices face barriers when an effective standard procedure does not exist, as is the case of the comparator procedure[28]. Our challenge in preparing the present protocol was the absence of a gold standard for pregnancy dating, since the fetal age begins upon conception; however, this information is difficult to be accurately determined[7].

References

1. Children St. Surviving the first day. In: *State of the world's mothers 2013.* Vol 1. Connecticut: Save the Children; 2013: http://www.savethechildren.org/atf/cf/%7B9def2ebe-10ae-432c-9bd0-df91d2eba74a%7D/SOWM-FULL-REPORT_2013.PDF.

2. França EB, Lansky S, Rego MAS, et al. Principais causas da mortalidade na infância no Brasil, em 1990 e 2015: estimativas do estudo de Carga Global de Doença. *Revista Brasileira de Epidemiologia.* 2017;20:46-60.

3. Federation StC. HNN website, Healthy Newborn Network. https://www.healthynewbornnetwork.org/. Published 2018. Accessed 01/17/2018, 2018.

4. Howson CP, Kinney MV, McDougall L, Lawn JE, Group BTSPBA. Born too soon: preterm birth matters. *Reprod Health.* 2013;10 Suppl 1:S1.

For peer review only - http://bmjopen.bmj.com/site/about/guidelines.xhtml

5. Bhutta ZA, Giuliani F, Haroon A, et al. Standardisation of neonatal clinical practice. *BJOG.* 2013;120 Suppl 2:56-63, v.

6. Liszewski MC, Stanescu AL, Phillips GS, Lee EY. Respiratory Distress in Neonates: Underlying Causes and Current Imaging Assessment. *Radiol Clin North Am.* 2017;55(4):629-644.

7. Committee on Obstetric Practice tAIoUiM, and the Society for Maternal-Fetal Medicine. Committee Opinion No 700: Methods for Estimating the Due Date. *Obstet Gynecol.* 2017;129(5):e150-e154.

8. Karl S, Li Wai Suen CS, Unger HW, et al. Preterm or not--an evaluation of estimates of gestational age in a cohort of women from Rural Papua New Guinea. *PLoS One.* 2015;10(5):e0124286.

9. Wilcox AJ, Weinberg CR, Baird DD. Timing of Sexual Intercourse in Relation to Ovulation — Effects on the Probability of Conception, Survival of the Pregnancy, and Sex of the Baby. *New England Journal of Medicine.* 1995;333(23):1517-1521.

10. Lynch CD, Zhang J. The research implications of the selection of a gestational age estimation method. *Paediatr Perinat Epidemiol.* 2007;21 Suppl 2:86-96.

11. Ballard JL, Khoury JC, Wedig K, Wang L, Eilers-Walsman BL, Lipp R. New Ballard Score, expanded to include extremely premature infants. *The Journal of Pediatrics.* 1991;119(3):417-423.

12. Lee AC, Panchal P, Folger L, et al. Diagnostic Accuracy of Neonatal Assessment for Gestational Age Determination: A Systematic Review. *Pediatrics.* 2017;140(6).

For peer review only - http://bmjopen.bmj.com/site/about/guidelines.xhtml

13. Nguyen TH, Larsen T, Engholm G, Møller H. Increased adverse pregnancy outcomes with unreliable last menstruation. *Obstet Gynecol.* 2000;95(6 Pt 1):867-873.

14. Reis ZSN, Guimarães RN, Inventors. Dispositivo para determinação da idade gestacional, processos e usos. Nov/1/2016, 2016.

15. Reis ZSN, Simeoni U, Vitral GLN, Souza IMFd, Rego MAS, Guimaraes RN. Newborn skin reflection: Proof of concept for a new approach for predicting gestational age at birth. A cross-sectional study. *Plos One.* 2017.

16. Reis ZSN, Vitral GLN, de Souza IMF, Rego MAS, Guimaraes RN. Newborn skin reflection: Proof of concept for a new approach for predicting gestational age at birth. A cross-sectional study. *PLOS ONE.* 2017;12(9):e0184734.

17. Kaplan AV, Baim DS, Smith JJ, et al. Medical device development: from prototype to regulatory approval. *Circulation.* 2004;109(25):3068-3072.

18. Brasil. Resolução da Diretoria Colegiada - RDC No. 10. In: Sanitária AnNdVn, ed. Vol 10. Brasilia: Diário Oficial da União, de 03 de março de 2015; 2015.

19. Papageorghiou AT, Kennedy SH, Salomon LJ, et al. International standards for early fetal size and pregnancy dating based on ultrasound measurement of crown-rump length in the first trimester of pregnancy. *Ultrasound Obstet Gynecol.* 2014;44(6):641-648.

20. Reuter S, Moser C, Baack M. Respiratory distress in the newborn. *Pediatr Rev.* 2014;35(10):417-428; quiz 429.

For peer review only

For peer review only - http://bmjopen.bmj.com/site/about/guidelines.xhtml

21. Faul F, Erdfelder E, Lang A-G, Buchner A. G* Power 3: A flexible statistical power analysis program for the social, behavioral, and biomedical sciences. *Behavior research methods.* 2007;39(2):175-191.

22. Wilson K, Hawken S, Murphy MS, et al. Postnatal Prediction of Gestational Age Using Newborn Fetal Hemoglobin Levels. *EBioMedicine.* 2017;15:203-209.

23. Nielsen J. Usability inspection methods. Conference Companion on Human Factors in Computing Systems; 1994; Boston, Massachusetts, USA.

24. Altman DG BJ. Measurement in medicine: the analysis of method comparison studies. 1983:307-317

25. Beydoun H, Ugwu B, Oehninger S. Assisted reproduction for the validation of gestational age assessment methods. *Reproductive biomedicine online.* 2011;22(4):321-326.

26. WHO. Clinical Trials in Children. Essential medicines for children. World Health Organisation. http://www.who.int/ictrp/child/en/. Accessed 08/01/2018, 2018.

27. USA. NIH's Definition of clinical trial. In: Services USDoHaH, ed. Bethesda: National Institutes of Health; 2018.

28. Neugebauer EAM, Rath A, Antoine SL, et al. Specific barriers to the conduct of randomised clinical trials on medical devices. *Trials.* 2017;18(1):427.

For peer review only - http://bmjopen.bmj.com/site/about/guidelines.xhtml

Author statement:

ZSNR: designed the study, planned data collection, prepared the team for good clinical practices, wrote and revised the paper. RNG, RAPLA, MASR, RMCR and JSG made substantial contributions to study design, planned data collection, prepared the team for good clinical practices, wrote and revised the paper. GLNV, MAAR, GSN, PJHN, MDRM, and MVN made contributions to standard procedures in methods, drafted the manuscript, reviewed the paper, and approved the final manuscript. EAC: drafted the work and reviewed it critically for important intellectual content, as statistic consultant.

Funding statement

This research was supported by the Grand Challenges Exploration from the Bill & Melinda Gates Foundation (Grant number OPP1128907, Contract), http://www.gatesfoundation.org/, and Fundação de Amparo a Pesquisa de Minas Gerais, Brazil, http://www.fapemig.br/en/, non-profit sectors. The clinical trial is funded by the Brazilian Ministry of Health, Program of Development of the Industrial Health Complex (PROCIS), project 23072.052747/2017-51, trial sponsor contact: Fotini Toscas, e-mail fotini.toscas@saude.gov.br. The funders played no role in the study design, data collection and analysis, decision to publish, or preparation of the manuscript.

Acknowledgements

The authors would like to thank Centre for Clinical Research of Hospital das Clínicas, Universidade Federal de Minas Gerais for general support and Good clinical practices

For peer review only - http://bmjopen.bmj.com/site/about/guidelines.xhtml

training; Fotini Toscas from the Brazilian Ministry of Health for the active intermediation as the trial sponsor contact.

Roles and responsibilities: ZSNR is the Principal Investigator and coordinator of the Directive Committee. JSG is the coordinator of the Data Management Team and will continuously receive report adverse events of trial interventions or trial conduct. RAPLA is the coordinator of the Clinical Trial Quality Committee, responsible for important protocol modifications, if necessary.

Conflict of interests statement

Authors declare a patent deposit on behalf of the Universidade Federal de Minas Gerais and Fundação de Amparo a Pesquisa de Minas Gerais, Brazil, http://www.fapemig.br/en/. The inventors were Reis, Zilma Silveira Nogueira and Guimaraes, Rodney Nascimento: BR1020170235688 (CTIT-PN862)[14].

Figure 1. Secondary outcome comparisons between the reference GA and the Preemie-Test in a simulated scenario without best pregnancy dating

Legends: *Gestational age from crown-rump-length data adjusted to Intergrowth's 21st fetal standard[19]. R: reference. GA: gestational age. T: test. C1: comparator 1 is the gestational age calculated using the first ultrasound exam after 13 weeks and 6 days and before 22 weeks of gestation. C2: comparator 2 is the gestational age based on a reliable last menstrual period.

For peer review only - http://bmjopen.bmj.com/site/about/guidelines.xhtml

Figure 2. Participant timeline of the study

Legends: GA: gestational age. R: reference. LMP: last menstrual period.

Word Count: 3415 words

For peer review only

For peer review only - http://bmjopen.bmj.com/site/about/guidelines.xhtml

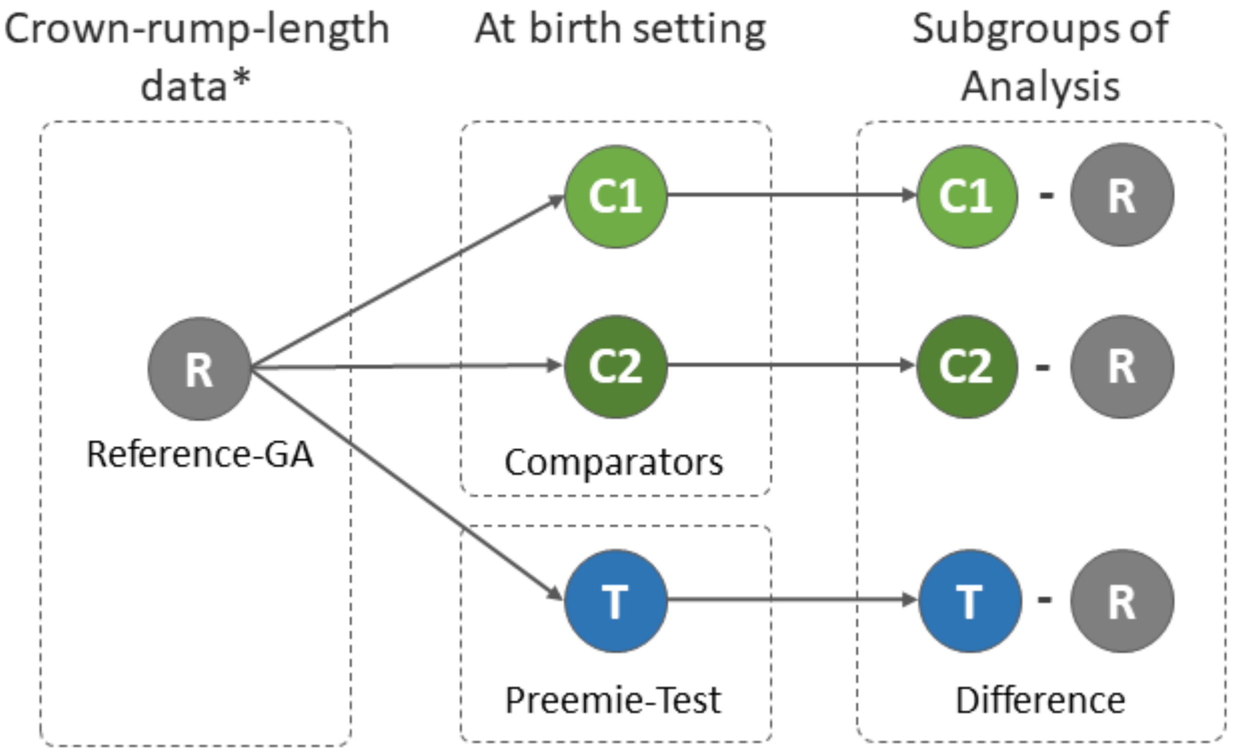
Crown-rump-length data*
At birth setting
Subgroups of Analysis
R
Reference-GA
C1
C2
Comparators
T
Preemie-Test
C1 - R
C2 - R
T - R
Difference

	STUDY PERIOD			
	Enrollment	Assessment	Close-out	Allocation
TIMEPOINT	$-t_1$	0	72 hours	Analysis
**ENROLLMENT:** Eligibility screen	X			
Informed consent	X			
**INTERVENTION:** Preemie-Test		X		
**ASSESSMENTS AND ANALYSIS:** Preemie-Test: data acquisition		X		
Reference GA: calculated by obstetric ultrasound at <14 weeks of gestation	X			X
Comparator 1: GA calculated by obstetric ultrasound at ≥ 14 and <22 weeks	X			X
Comparator 2: GA calculated by reliable LMP	X			X
Case-control nested study: lung maturity				

Fig. 2. Participant timeline of the study

GA: gestational age. R: reference. LMP: last menstrual period.

For peer review only

TERMO DE CONSENTIMENTO LIVRE E ESCLARECIDO

Detecção da prematuridade através da interação entre a luz e a pele neonatal: a validação do Preemie-Teste

Sob responsabilidade da pesquisadora Profa Zilma Silveira Nogueira Reis

Cara senhora, você está sendo convidada a participar deste estudo porque acaba de ter um parto no hospital (nome do hospital do centro colaborador) ____.

Apresentação do estudo

O objetivo deste estudo é descobrir novas técnicas para estimar a idade de um bebê ao nascer e identificar aqueles que nasceram antes de nove meses, os prematuros. A idade gestacional desconhecida pode aumentar o risco dos bebês no momento de seu nascimento. As técnicas atuais para se estimar a idade do bebê possuem grande margem de erro.

Acreditamos que a pele possui características que, se bem estudadas, podem refletir a idade das pessoas, e também dos bebês. Por isso, estamos desenvolvendo um novo equipamento médico que se encontra em teste. Ele utiliza a luz para avaliar a composição da pele do bebê e detectar sua idade. Os resultados poderão beneficiar os bebês que nascem sem a informação confiável da idade gestacional.

Instituições envolvidas no estudo

O estudo é desenvolvido pela Faculdade de Medicina da Universidade Federal de Minas Gerais (UFMG), em cooperação com maternidades brasileiras, entre elas a que você se encontra internada. A previsão deste estudo é que 787 crianças recém-nascidas sejam examinadas.

A participação no estudo, riscos e cuidados

Convidamos você e seu bebê para participar deste estudo. Isso incluirá um exame na pele do bebê com a luz, uma breve entrevista com você e a consulta aos registros de saúde sobre a gravidez e os do seu bebê neste hospital. Na entrevista serão tomados todos os cuidados a fim de minimizar os constrangimentos para você. A consulta ao prontuário médico será realizada resguardando o direito de sigilo da informação. Pedimos sua permissão para fotografar a caderneta da gestante ou outro documento equivalente, para conferir a idade gestacional calculada pelos ciclos menstruais e pelos exames de ultrassom. As partes da fotografia que contenham sua identificação serão retiradas da imagem e a manteremos até o final do estudo, quando o arquivo será apagado dos registros da pesquisa.

Pedimos sua permissão para fazer um exame na pele de seu bebê, na região da sola do pé, usando um equipamento em teste. O exame é indolor e externo ao corpo, considerado não-invasivo. A parte que encosta no bebê é pequena e não apresenta pontas que possam ferir a sua pele. Outros equipamentos parecidos, que emitem luz, já são usados nos bebês de forma segura. Por exemplo o oxímetro que faz teste do coraçãozinho. Assim como esse, não se espera que ocorram efeitos imediatos ou futuros na saúde do bebê. Os riscos do teste que faremos incluem a exposição do pé do bebê com perda temporária de calor do corpo e estresse. Cuidados serão tomados a fim de minimizar estes desconfortos. Esclarecemos que o teste dura alguns segundos reduzindo ao mínimo chance de causar marcas ou irritação no local. Caso seu bebê apresente sinais de desconforto durante o exame, o mesmo será interrompido. Você ou familiares poderão permanecer junto ao seu filho durante o exame. Nas crianças que estiverem na Unidade Neonatal, o exame será realizado onde ela já está sendo cuidada, acompanhado pelo profissional de saúde que já está cuidando dela. Caso o seu bebê seja prematuro, todos os devidos cuidados serão tomados antes de cada exame para reduzir a chance de perda de calor, seguindo todas as recomendações de um bebê que fica em incubadora.

Esclarecemos que este estudo não trará benefícios diretos a você ou seu filho, entretanto auxiliará na validação de um novo teste que poderá no futuro identificar o bebê prematuro. Os resultados poderão

For peer review only - http://bmjopen.bmj.com/site/about/guidelines.xhtml

também gerar informações que ajudem a melhorar os cuidados com outros bebês, quando a idade gestacional é desconhecida. Informamos que os resultados da pesquisa serão publicados em revistas científicas e apresentados em congressos, sem contudo revelar sua identidade ou a do bebê. As informações obtidas durante a pesquisa serão confidenciais, guardadas em computadores, protegidos por senha e não serão usadas para outros fins. O roubo das informações que coletaremos no estudo é um risco remoto. Para isso, as melhores práticas em segurança de dados serão empregadas. Também poderão ter acesso aos dados da pesquisa o comitê que coordena o estudo, assim como a agência reguladora ANVISA, sem jamais violar a confidencialidade e privacidade dos dados, para que seja possível monitorar se os procedimentos de qualidade e segurança da pesquisa estão sendo seguidos.

Seus direitos como participante

Informamos que a sua participação deve ser voluntária, ou seja, não é obrigatória e caso não concorde ou resolva desistir a qualquer momento isto não trará nenhum constrangimento para você ou para a forma como você será tratada neste hospital. Também não está previsto nenhum tipo de pagamento por sua participação na pesquisa. Este estudo não implica em gastos para você, pois não terá que se deslocar para outro local, permanecer mais tempo no hospital, uma vez que o exame é feito durante sua internação e de seu bebê na maternidade. Caso seja de seu interesse, os resultados do exame que estarão guardados com o pesquisador e lhe serão entregues assim que você solicitar.

Os pesquisadores garantem que acompanharão gratuitamente seu bebê durante a realização do exame e a qualquer momento que se fizer necessário, em qualquer problema que por ventura esteja associado ao estudo ou efeito do teste com a luz.

Este Termo de Consentimento está elaborado em duas vias iguais. Ambas devem ser assinadas por você, pelo pai da criança e pelo pesquisador. Uma via ficará com o participante e a outra com o pesquisador.

O Comitê de Ética em Pesquisa da UFMG pode ser contatado em caso de haver dúvidas quanto aos aspectos éticos da pesquisa, através do telefone (31) 3409-4592 ou endereço completo apresentado a seguir.

Meu nome	
Documento de identidade	
Data de hoje	

Eu declaro que estou em condições de tomar esta decisão e ciente do que foi exposto acima. Autorizo o uso de minhas informações de saúde e as do meu bebê para este projeto de pesquisa, assim como a realização do novo teste. Participo voluntariamente deste estudo e estou ciente que o exame na pele do meu bebê com a luz não traz prejuízo à sua saúde

Assinatura da puérpera:
Assinatura do pai da criança:
Assinatura do pesquisador:

Telefones de contato:
Maternidade Hospital das Clínicas da UFMG – (31) 34099422

Rubrica dos pais: ____________

For peer review only - http://bmjopen.bmj.com/site/about/guidelines.xhtml

Hospital (nome e telefone do hospital colaborador)
Zilma Reis – (31) 985177473 e-mail: skinage.ufmg@gmail.com
Comitê de Ética em Pesquisa da UFMG – Av. Prof. Antônio Carlos, 6627, Unidade Administrativa II, 2º andar, sala 2005, Campus Pampulha, CEP: 31270-901. E-mail:coep@prpq.ufmg.br. Fone (31) 34094592.
Comitê de Ética em Pesquisa do centro colaborador e endereço completo, com e-mail.

For peer review only

For peer review only - http://bmjopen.bmj.com/site/about/guidelines.xhtml

Reporting checklist for protocol of a clinical trial.

Based on the SPIRIT guidelines.

Instructions to authors

Complete this checklist by entering the page numbers from your manuscript where readers will find each of the items listed below.

Your article may not currently address all the items on the checklist. Please modify your text to include the missing information. If you are certain that an item does not apply, please write "n/a" and provide a short explanation.

Upload your completed checklist as an extra file when you submit to a journal.

In your methods section, say that you used the SPIRIT reporting guidelines, and cite them as:

Chan A-W, Tetzlaff JM, Altman DG, Laupacis A, Gøtzsche PC, Krleža-Jerić K, Hróbjartsson A, Mann H, Dickersin K, Berlin J, Doré C, Parulekar W, Summerskill W, Groves T, Schulz K, Sox H, Rockhold FW, Rennie D, Moher D. SPIRIT 2013 Statement: Defining standard protocol items for clinical trials. Ann Intern Med. 2013;158(3):200-207

		Reporting Item	Page Number
Title	#1	Descriptive title identifying the study design, population, interventions, and, if applicable, trial acronym	1
Trial registration	#2a	Trial identifier and registry name. If not yet registered, name of intended registry	2
Trial registration: data set	#2b	All items from the World Health Organization Trial Registration Data Set	2
Protocol version	#3	Date and version identifier	6
Funding	#4	Sources and types of financial, material, and other support	18
Roles and responsibilities: contributorship	#5a	Names, affiliations, and roles of protocol contributors	19
Roles and responsibilities:	#5b	Name and contact information for the trial sponsor	19

For peer review only

sponsor contact information			
Roles and responsibilities: sponsor and funder	#5c	Role of study sponsor and funders, if any, in study design; collection, management, analysis, and interpretation of data; writing of the report; and the decision to submit the report for publication, including whether they will have ultimate authority over any of these activities	18
Roles and responsibilities: committees	#5d	Composition, roles, and responsibilities of the coordinating centre, steering committee, endpoint adjudication committee, data management team, and other individuals or groups overseeing the trial, if applicable (see Item 21a for data monitoring committee)	18
Background and rationale	#6a	Description of research question and justification for undertaking the trial, including summary of relevant studies (published and unpublished) examining benefits and harms for each intervention	4
Background and rationale: choice of comparators	#6b	Explanation for choice of comparators	4
Objectives	#7	Specific objectives or hypotheses	5
Trial design	#8	Description of trial design including type of trial (eg, parallel group, crossover, factorial, single group), allocation ratio, and framework (eg, superiority, equivalence, non-inferiority, exploratory)	6
Study setting	#9	Description of study settings (eg, community clinic, academic hospital) and list of countries where data will be collected. Reference to where list of study sites can be obtained	6
Eligibility criteria	#10	Inclusion and exclusion criteria for participants. If applicable, eligibility criteria for study centres and individuals who will perform the interventions (eg, surgeons, psychotherapists)	7
Interventions: description	#11a	Interventions for each group with sufficient detail to allow replication, including how and when they will be administered	7-8
Interventions: modifications	#11b	Criteria for discontinuing or modifying allocated interventions for a given trial participant (eg, drug dose change in response	9

For peer review only

		to harms, participant request, or improving / worsening disease)	
Interventions: adherance	#11c	Strategies to improve adherence to intervention protocols, and any procedures for monitoring adherence (eg, drug tablet return; laboratory tests)	NA
Interventions: concomitant care	#11d	Relevant concomitant care and interventions that are permitted or prohibited during the trial	9
Outcomes	#12	Primary, secondary, and other outcomes, including the specific measurement variable (eg, systolic blood pressure), analysis metric (eg, change from baseline, final value, time to event), method of aggregation (eg, median, proportion), and time point for each outcome. Explanation of the clinical relevance of chosen efficacy and harm outcomes is strongly recommended	9-10
Participant timeline	#13	Time schedule of enrolment, interventions (including any run-ins and washouts), assessments, and visits for participants. A schematic diagram is highly recommended (see Figure)	11
Sample size	#14	Estimated number of participants needed to achieve study objectives and how it was determined, including clinical and statistical assumptions supporting any sample size calculations	11-12
Recruitment	#15	Strategies for achieving adequate participant enrolment to reach target sample size	7
Allocation: sequence generation	#16a	Method of generating the allocation sequence (eg, computer-generated random numbers), and list of any factors for stratification. To reduce predictability of a random sequence, details of any planned restriction (eg, blocking) should be provided in a separate document that is unavailable to those who enrol participants or assign interventions	NA
Allocation concealment mechanism	#16b	Mechanism of implementing the allocation sequence (eg, central telephone; sequentially numbered, opaque, sealed envelopes), describing any steps to conceal the sequence until interventions are assigned	NA
Allocation: implementation	#16c	Who will generate the allocation sequence, who will enrol participants, and who will assign participants to interventions	NA

For peer review only

Blinding (masking)	#17a	Who will be blinded after assignment to interventions (eg, trial participants, care providers, outcome assessors, data analysts), and how	8
Blinding (masking): emergency unblinding	#17b	If blinded, circumstances under which unblinding is permissible, and procedure for revealing a participant's allocated intervention during the trial	NA
Data collection plan	#18a	Plans for assessment and collection of outcome, baseline, and other trial data, including any related processes to promote data quality (eg, duplicate measurements, training of assessors) and a description of study instruments (eg, questionnaires, laboratory tests) along with their reliability and validity, if known. Reference to where data collection forms can be found, if not in the protocol	12
Data collection plan: retention	#18b	Plans to promote participant retention and complete follow-up, including list of any outcome data to be collected for participants who discontinue or deviate from intervention protocols	12
Data management	#19	Plans for data entry, coding, security, and storage, including any related processes to promote data quality 7(eg, double data entry; range checks for data values). Reference to where details of data management procedures can be found, if not in the protocol	12
Statistics: outcomes	#20a	Statistical methods for analysing primary and secondary outcomes. Reference to where other details of the statistical analysis plan can be found, if not in the protocol	12
Statistics: additional analyses	#20b	Methods for any additional analyses (eg, subgroup and adjusted analyses)	12
Statistics: analysis population and missing data	#20c	Definition of analysis population relating to protocol non-adherence (eg, as randomised analysis), and any statistical methods to handle missing data (eg, multiple imputation)	NA
Data monitoring: formal committee	#21a	Composition of data monitoring committee (DMC); summary of its role and reporting structure; statement of whether it is independent from the sponsor and competing interests; and reference to where further details about its charter can be	18

For peer review only

		found, if not in the protocol. Alternatively, an explanation of why a DMC is not needed	
Data monitoring: interim analysis	#21b	Description of any interim analyses and stopping guidelines, including who will have access to these interim results and make the final decision to terminate the trial	NA
Harms	#22	Plans for collecting, assessing, reporting, and managing solicited and spontaneously reported adverse events and other unintended effects of trial interventions or trial conduct	19
Auditing	#23	Frequency and procedures for auditing trial conduct, if any, and whether the process will be independent from investigators and the sponsor	12
Research ethics approval	#24	Plans for seeking research ethics committee / institutional review board (REC / IRB) approval	6
Protocol amendments	#25	Plans for communicating important protocol modifications (eg, changes to eligibility criteria, outcomes, analyses) to relevant parties (eg, investigators, REC / IRBs, trial participants, trial registries, journals, regulators)	19
Consent or assent	#26a	Who will obtain informed consent or assent from potential trial participants or authorised surrogates, and how (see Item 32)	6
Consent or assent: ancillary studies	#26b	Additional consent provisions for collection and use of participant data and biological specimens in ancillary studies, if applicable	NA
Confidentiality	#27	How personal information about potential and enrolled participants will be collected, shared, and maintained in order to protect confidentiality before, during, and after the trial	6
Declaration of interests	#28	Financial and other competing interests for principal investigators for the overall trial and each study site	18
Data access	#29	Statement of who will have access to the final trial dataset, and disclosure of contractual agreements that limit such access for investigators	7
Ancillary and post trial care	#30	Provisions, if any, for ancillary and post-trial care, and for compensation to those who suffer harm from trial participation	NA

For peer review only

Dissemination policy: trial results	#31a	Plans for investigators and sponsor to communicate trial results to participants, healthcare professionals, the public, and other relevant groups (eg, via publication, reporting in results databases, or other data sharing arrangements), including any publication restrictions	7
Dissemination policy: authorship	#31b	Authorship eligibility guidelines and any intended use of professional writers	NA
Dissemination policy: reproducible research	#31c	Plans, if any, for granting public access to the full protocol, participant-level dataset, and statistical code	7
Informed consent materials	#32	Model consent form and other related documentation given to participants and authorised surrogates	6
Biological specimens	#33	Plans for collection, laboratory evaluation, and storage of biological specimens for genetic or molecular analysis in the current trial and for future use in ancillary studies, if applicable	NA

The SPIRIT checklist is distributed under the terms of the Creative Commons Attribution License CC-BY-ND 3.0. This checklist can be completed online using https://www.goodreports.org/, a tool made by the EQUATOR Network in collaboration with Penelope.ai

For peer review only

Prematurity detection evaluating the interaction between newborn skin and light: The Preemie-Test Multicenter Clinical Trial in Brazilians' hospitals to validate a new medical device

Journal:	*BMJ Open*
Manuscript ID	bmjopen-2018-027442.R1
Article Type:	Protocol
Date Submitted by the Author:	23-Nov-2018
Complete List of Authors:	Reis, Zilma; Universidade Federal de Minas Gerais, Gynecology and Obstetrics<br>Guimarães, Rodney; Faculty of Medicine, Universidade Federal de Minas Gerais, Ginecologia e Obstetrícia<br>Rego, Maria Albertina; Faculty of Medicine, Universidade Federal de Minas Gerais, Pediatrics<br>Maia de Castro Romanelli , Roberta ; Universidade Federal de Minas Gerais Faculdade de Medicina,<br>Gaspar, Juliano; Faculty of Medicine, Universidade Federal de Minas Gerais, Obstetrics and Gynecology<br>Vitral, Gabriela; Faculty of Medicine, Universidade Federal de Minas Gerais<br>dos Reis, Marconi; Faculty of Medicine, Universidade Federal de Minas Gerais, Pediatrics<br>Colósimo, Enrico; Universidade Federal de Minas Gerais, Statistics<br>Neves, Gabriela; Hospital Sofia Feldman<br>Vale, Marynea; Hospital Universitario da Universidade Federal do Maranhao, Pediatrics<br>Nader, Paulo; Universidade Luterana do Brasil. Hospital Universitário de Canoas, Pediatrics<br>de Moura, Martha; Hospital Materno Infantil de Brasília<br>de Aguiar, Regina ; Faculty of Medicine, Universidade Federal de Minas Gerais, Obstetrics and Gynecology
**Primary Subject Heading**:	Obstetrics and gynaecology
Secondary Subject Heading:	Global health, Diagnostics, Paediatrics
Keywords:	Gestational Age, Infant, Premature, Skin Physiological Phenomena, Photomedicine, Equipment and Supplies

Prematurity detection evaluating the interaction between newborn skin and light: The Preemie-Test Multicenter Clinical Trial in Brazilians' hospitals to validate a new medical device

Zilma Silveira Nogueira Reis[1]*; Rodney Nascimento Guimarães[1]; Maria Albertina Santiago Rego[1]; Roberta Maia de Castro Romanelli[1]; Juliano de Souza Gaspar[1]; Gabriela Luíza Nogueira Vitral[1]; Marconi Augusto Aguiar dos Reis[1]; Enrico Antônio Colósimo[2]; Gabriela Silveira Neves[3]; Marynea Silva do Vale[4]; Paulo de Jesus Nader[5]; Martha David Rocha de Moura[6]; Regina Amélia Pessoa Lopes de Aguiar[1]

1 - Faculty of Medicine, Universidade Federal de Minas Gerais, Brazil.

2 - Statistics Department, Universidade Federal de Minas Gerais, Brazil.

3 - Hospital Sofia Feldman, Brazil.

4 - Hospital Universitário da Universidade Federal do Maranhão, Brazil.

5 - Universidade Luterana do Brasil. Hospital Universitário de Canoas, Brazil.

6 - Hospital Materno Infantil de Brasília, Brazil.

*Corresponding author: Zilma Silveira Nogueira Reis. Avenida Professor Alfredo Balena, 190, sala 601. Zip Code 30.130.100 Belo Horizonte, Brazil. Phone: +55 0 31 3409-9467. Email: zilma@medicina.ufmg.br.

Abstract

For peer review only - http://bmjopen.bmj.com/site/about/guidelines.xhtml

For peer review only

Introduction: Recognizing prematurity is critical in order to attend to immediate needs in childbirth settings, guiding the extent of medical care provided for newborns. A new medical device has been developed to carry out the Preemie-Test, an innovative approach to estimate gestational age (GA), based on the photobiological properties of the newborn's skin. This study will validate the Preemie-Test for GA estimation at birth and its accuracy to detect prematurity. Secondarily, the study intends to associate the infant´s skin reflectance with lung maturity, as well as evaluate safety, precision, and usability of a new medical device to offer a suitable product for health professionals during childbirth and in neonatal care settings.

Methods and analysis: Research protocol for diagnosis, single-group, single-blinding, and single-arm multicenter clinical trials with a reference standard. Alive newborns, with 24 weeks or more of pregnancy age, will be enrolled during the first 24 hours of life. Sample size is 787 subjects. The primary outcome is the difference between the GA calculated by the photobiological neonatal skin assessment methodology and the GA calculated by the comparator antenatal ultrasound or reliable last menstrual period. Immediate complications caused by pulmonary immaturity during the first 72 hours of life will be associated with skin reflectance in a nested case-control study.

Ethics and dissemination: Each local independent ethics review board approved the trial protocol. The authors intend to share the minimal anonymized data set necessary to replicate study findings.

Trial registration number: WHO Clinical Trial http://apps.who.int/trialsearch/Trial2.aspx?TrialID=RBR-3f5bm5.

Key-words: Gestational Age, Infant, Premature; Skin Physiological Phenomena; Photomedicine; Equipment and Supplies.

For peer review only - http://bmjopen.bmj.com/site/about/guidelines.xhtml

Article Summary

Strengths and limitations of this study:

- The study has the potential to validate a new approach for pregnancy dating.
- The device will be subjected to high-quality clinical study to demonstrate benefits.
- The gold standard comparator for pregnancy dating does not exist, instead a reference standard will be used with blinded primary outcome.
- The agreement endpoint between methods for gestational age determination precluded randomization of the intervention.

For peer review only

Introduction

In childbirth settings, health professionals continuously need to make timely decisions to provide proper neonatal care. The day of birth is the riskiest for newborns and mothers almost everywhere[1]. Perinatal causes related to prematurity and complications during childbirth, which are generally preventable through qualified health care, are the primary causes of death among newborns[1,2]. Most of these deaths took place in countries with low resources and a scarcity of health facilities[3]. The opportune recognition of prematurity is critical in order to judge the viability of the newborn and to attend to his/her immediate needs, guiding the complexity of the medical care provided for the newborn. Without reliable information on the age of the unborn phase, actions to preserve the potential for survival of the newborn can be neglected[4]. Indeed, the attempted management of the risk of mortality and severe complications are sensitive issues to the gestational age (GA), which involves temperature maintenance, ventilatory support, transport to a neonatal intensive care unit (NICU), and the early treatment of respiratory distress syndrome (RDS), the most severe complication of premature birth[5]. In addition to the GA information or birthweight, the prediction of neonatal respiratory morbidity may be critical in planning immediate medical care [6], since the respiratory system is among the last of the fetal organ systems to mature, which is associated with enhanced morbidity and mortality[6].

Current methods of dating pregnancy remain a worldwide challenge. Early obstetric ultrasound currently offers the best due date[7]. However, access to this type of exam is limited because of high equipment costs, poor training and skills of health professionals, or late prenatal care[8]. Despite a 10-days or more margin of error during the second and third trimester of gestation, ultrasound is still a reasonable methodology for GA determination, when the best opportunity was lost[7]. The calculation, based on the historical information of the last menstrual period (LMP), is impacted by the uncertainty

For peer review only

of both the fertility days and date of conception[9], due to the bias of memory, the use of hormonal contraception, and breastfeeding[10]. After birth, neurological scores, such as the New Ballard[11], show a tendency to overestimate GA in preterm infants and underestimate GA in growth-restricted infants[12]. Efforts to enhance the reliability of pregnancy dating, through more accurate and accessible technologies, seek to improve pregnancy outcomes and neonatal survival[13].

A new medical device has been developed to carry out the Preemie-Test, an innovative approach used to estimate GA, based on the photobiological properties of the newborn's skin. This reflective test is noninvasive, and the device automatically processes the light, scattered by the constituents of the skin layers, when a small optoelectronic light emitter/receiver sensor touches the newborn's skin[14]. the device under test is easy to use and every effort is being made to ensure that it has excellent accuracy, be it safe and low cost. The feasibility study provides a mathematical model to predict GA based on the skin reflectance adjusted to clinical variables (R^2 = 0.828, P <0.001)[15]. However, before the adoption or use of an innovation, an effectiveness trial of intervention is a critical step in the research chain regarding its the social utility when completing the translation from the proof of concept to clinical science [16]. The rationale for the main hypothesis in this study is that the skin maturity of a newborn, obtained by the analysis of its optical properties, is useful in pregnancy dating for clinical use and respiratory prognosis, especially in a scenario with no reliable GA based on current methods. This study aims to validate the photobiological model of the skin, called the "Preemie-test", in order to estimate GA at birth and determine its accuracy in detecting prematurity. Secondarily, it also seeks to associate the infant's skin reflectance with lung maturity. Moreover, this study intends to evaluate the safety, precision, and the usability of a new medical device to offer a suitable product to support health professionals during childbirth and in neonatal care settings.

For peer review only - http://bmjopen.bmj.com/site/about/guidelines.xhtml

Methods

Study design

This study will use a protocol for diagnosis, single-group, single-blinding, and single-arm multicenter clinical trials with a reference standard. This new photobiological approach to the skin, gathered in a medical device, is currently in the pivotal phase of innovation development from the prototype to regulatory approval [17]. This step aims to provide the translation[16] of the scientific model for GA detection based on skin maturity. This Protocol version is 1, July/10th/2018. Faculty of Medicine, Universidade Federal de Minas Gerais is the Coordinator Center.

Study Settings, Ethics and Dissemination

Selected Brazilian referral centers for high-risk pregnancy and neonatal care will participate in the study, according to this protocol: Hospital das Clínicas, Universidade Federal de Minas Gerais, as the Center for Coordination; Hospital Sofia Feldman, Minas Gerais State; Hospital da Universidade Luterana do Brasil, Rio Grande do Sul State; Hospital Materno-infantil de Brasília, Distrito Federal; and Hospital Universitário da Universidade Federal do Maranhão, Maranhão State. Each local independent ethics review board approved the trial protocol, and the Brazilian National Research Council (CONEP) approved all study activities and protocol prior to the commencement of study activities, in accordance with the Declaration of Helsinki (2008), good clinical practice as set forth by the International Organization for Standardization (ISO) 14155:2011, and the Brazilian regulatory health agency's recommendations[18]. This study was logged under both protocol number CAAE 81347817.6.1001.5149 and the International Clinical Trials Registry Platform under Universal Trial Number U1111-1205-0539 is accessible by http://apps.who.int/trialsearch/Trial2.aspx?TrialID=RBR-3f5bm5. Parents will sign an informed consent form on behalf of the newborn before participating in the clinical trial (supplementary file).

For peer review only - http://bmjopen.bmj.com/site/about/guidelines.xhtml

For peer review only

Data Sharing Statement

The authors intend to share the minimal anonymized data set necessary to replicate study findings. Data sharing will include: the reference and comparators GA, GA estimated by the Preemie-test, birth weight, RDS or transient tachypnea of the newborn (TTN) diagnosis, ventilatory support due to pulmonary immaturity, neonatal intensive care unit (NICU) admission due to RDS or TTN, and any adverse events regarding device's safety. Unidentified data and study-related documents as ethical approvals will be accessible by URLs for researchers, regulatory agencies, and sponsors.

Patient and Public Involvement

Patients and the public were not involved in the design of this study. The results will be disseminated to study parents of participants through scientific publications, non-scientific publications, and on the website of the project: http://skinage.medicina.ufmg.br.

Eligibility criteria and participant's timeline

A prospective sequential and concurrent enrollment process will select newborns in referral hospitals centers for neonatal care. Infants are eligible with the following inclusion criteria: (1) alive newborn; (2) enrollment during first 24 hours of life; (3) be 24 weeks or more of gestational age, at birth; (4) fetus underwent an obstetric ultrasound assessment before 14 weeks of pregnancy; (5) fetus also had obstetric ultrasound assessment between 14 and 22 gestational weeks. Exclusion criteria are: (1) malformation with structural skin alterations; (2) skin modifiers: anhydramnios, hydrops, congenital skin diseases or chorioamnionitis. Randomisation was not appropriate to assess the agreement between different methods to assess pregnancy dating.

In a nested case-control study, we will select newborns within the first 72 hours of life, discharge, or death, whichever occurs first, with the following inclusion criteria: (1) RDS

For peer review only - http://bmjopen.bmj.com/site/about/guidelines.xhtml

or (2) TTN diagnosis. Ranges of gestational age will randomly pair controls. Exclusion criteria include: (1) the existence of extra pulmonary conditions with tachypnea not due to prematurity and (2) diagnosis of Clinical or Laboratory-Confirmed Bloodstream Infection.

Intervention: The Preemie-Test

The Preemie-Test assessment occurs as soon as possible after birth, in the first 24 hours, inside incubators, open heating crib, common crib or in the mother's lap, in order to ensure minimum manipulation and stable clinical conditions. The acquisitions of all newborns will be stored in a database for further statistical analysis.

A noninvasive, handheld optoelectronic prototype has been developed to measure the backscattered light signal from the skin[15]. The equipment regulates the emitted light and processes the received light signal in the sensor, resulting in the prediction of GA by a mathematical model, associated or not with clinical variables. According to the Brazilian regulatory health agency (ANVISA), this medical device is categorized as a Class II safety: noninvasive and medium risk. The prototype unit of measurement and the process of GA estimation were patented under number BR1020170235688 (CTIT-PN862)[14]. An updated version of the invention received improvements in order to safeguard reliability and to minimize examiner interferences on the skin's backscattering acquisition. The light emitting-sensor touches the skin over the sole of the foot for a few seconds. The skin reflectance will be sensed once the light has been emitted by a light emitting diode (LED) at wavelengths from 400 nm to 1200 nm. Data acquisitions occur automatically, without operator influence, and are obtained three times per newborn, in the same site and sequentially. Digital recordings will be uploaded to a server for further analysis. The prototype will blind the examiner to the predicted GA value.

For peer review only - http://bmjopen.bmj.com/site/about/guidelines.xhtml

The criterium for discontinuing the interventions for a given trial participant will be in case of parents of the newborns' request.

Training and monitoring

Systematic monitoring of data collection, through an electronic information system, would trigger any adverse event. This medical team is still responsible for the training of healthcare professionals to recruit participants, data collection, a safely performed Preemie-Test during the newborn's assessment, and the monitoring of data quality. The certification of co-participant centers involved the accomplishment of at least 30 simulated examinations by the participant health professionals in the study.

Gestational age methods of calculation and comparators

Reference-GA (R) is calculated upon enrollment, using the embryo measurement assessed by ultrasound exam at <14 weeks of gestation as a reference. Crown-rump-length (CRL) data, recorded from the ultrasound report or prenatal care book-document, will be considered the crude data, when available. Intergrowth's 21st standard curve for ultrasound measurements from 7 weeks and 3 days up to 13 weeks and 6 days will be adjusted to all GA data, according to CRL[19].

GA methods to calculate GA in the childbirth setting, and their comparators are as follows:

- Preemie-Test-GA (T): data statistically determined by analyzing the acquired information stored in the device's processor.
- Comparators-GA (C): calculated using the first ultrasound exam after 13 weeks and 6 days of gestation and before 22 weeks (C1). When available, a second comparator is GA based on a reliable LMP (C2)[13].

For peer review only - http://bmjopen.bmj.com/site/about/guidelines.xhtml

We will take a scanning copy of the prenatal care book or the ultrasound report. After evaluating the data quality, the images will be discarded. To achieve a reliable LMP, we will interview the woman, as suggested by Nguyen et al. (2000)[13].

Primary outcome measures

The primary target is the agreement between the GA offered by the Preemie-Test (T) and the GA calculated by the comparators (C1 and C2), so as to perform the new test in scenarios without the Reference-GA (R). The outcome is the difference between the GA calculated by the photobiological neonatal skin assessment methodology in relation to the age calculated by the comparators.

Another measure for the primary target is the detection of preterm newborns, considering the age before 37 weeks of pregnancy as the threshold between term and preterm births, and analyzing sub-categories of preterm birth, based on GA[4]:

- extremely preterm (less than 28 weeks)
- very preterm (28 to 32 weeks)
- moderate to late preterm (more than 32 to less than 37 weeks).

In this case, the outcome is the proportion of the preterm newborn correctly detected at birth, based on the photobiological test of the skin, within a one-week error.

Secondary outcome measures

1. In a simulated scenario, in which the Reference-GA (R) is unknown, two groups will be randomly assigned from the complete database in order to compare differences among the Reference-GA (R), the GA obtained through the Preemie-Test (T), and the GA calculated by the comparators. Figure 1 presents such subgroups and measures for comparison.

For peer review only - http://bmjopen.bmj.com/site/about/guidelines.xhtml

2. To monitor the device's safety when in regular use by participants over a 72-hour period. Adverse events will be monitored, according to ISO 14155:2011 standards. This means any unexpected medical events, unintended disease or injury, or unfortunate clinical signs in subjects, users, or other people, whether related to the investigational medical device or not.

3. To establish the *ease of use* of the Preemie-Test measurement as a potential method for preterm newborn diagnosis.

The secondary outcome measures in the case-control nested study

Immediate complications, occurring during the first 72 hours of life due to pulmonary immaturity, are the secondary target. The outcome measures are as follows:

- To describe the relationship of the measurement of the newborn's skin reflectance with RDS and with diagnoses based on clinical and radiological findings and respiratory outcomes[6,20].
- To describe the relationship of the measurement of the newborn's skin reflectance with the TTN and with diagnoses based on clinical findings and respiratory outcomes[6].
- To describe the relationship of the measurement of the newborn's skin reflectance with ventilatory support due to pulmonary immaturity.
- To describe the relationship of the measurement of the newborn's skin reflectance with NICU admission due to RDS or TTN.

Time schedule of enrollment, intervention, and outcome measurements are presented in a schematic diagram (see Figure 2). The assessment occurs during the first 24 hours of life, but participants will be followed up for 72 hours or until discharge or death, whichever occurs first, for the monitoring of neonatal outcomes and adverse events.

Sampling and sample size

For peer review only - http://bmjopen.bmj.com/site/about/guidelines.xhtml

The sample size calculation is estimated based on the primary endpoint. To test the hypothesis of equivalence between the Preemie-Test GA and the comparators GA, a sample of 787 subjects is necessary to detect an effect size of 10%. Using the G-Power 3.1 software[21], we assumed an alpha error of 0.05, and a power of test of 0.80 to support a paired t-test.

Sampling intends to arrange three groups of GA enrollment to preserve enough premature newborns with 3:2:1 proportion, similar to Wilson et al. (2017)[22]: 392 term newborns, 263 premature newborns from 32 to 36 weeks and six days of GA, and 132 extremely premature newborns from 24 to 31 weeks and six days of GA.

Usability

The usability assessment will be performed by applying a checklist to participants who use the prototype device to perform the Preemie-Test. The 10 heuristics proposed by Nielsen and Marck |(1994)[23] will be adapted to build a checklist to evaluate the device, namely: (a) system visibility, (b) correspondence with the real world, (c) user control and freedom, (d) consistency of results and standardization, (e) error prevention, (f) visual recognition rather than memorization, (g) flexibility and efficiency of use, (h) esthetic and minimalist design, (i) help for the user to recognize, diagnose, and recover from errors, and (j) user documentation and help.

Data collection

Standard operational procedures set data entries in structured questionaries. In this concurrent clinical trial, an electronic information system was developed to collect data in different hospitals, simultaneously. Entry forms validations were implemented with data values ranges to ensure the quality of the information. An audit of the data will be permanently performed and the data summary available on the project webpage. Double system, paper-based and electronic will permit audit concerning reliability and validity.

For peer review only - http://bmjopen.bmj.com/site/about/guidelines.xhtml

Independent rater over-read all papers files and cross check with the electronic information from all patients.

Data analysis

Demographics and baseline characteristics of the study group, as well the intervention measurements, will be summarized by the frequencies and the mean and standard deviation (SD), the whereas median and interquartile range will be preferred for non-normally distributed continuous variables.

To model the GA prediction, computational randomization will select two subsamples in the database. One of them to train the prediction model of GA based on skin reflectance and clinical variables, such as sex, time in an incubator, phototherapy, birth weight, among others. Another part will be for the analytical validation of the predictive model. Improvements in the existing prediction models for GA (Preemie-Test), will be conducted with conventional statistical and data mining analyses.

Regarding the primary endpoint, the agreement among three methods for GA will be calculated using the Intraclass coefficient correlation and Bland & Altman plots[24], and paired t-testing. The accuracy of the Preemie-Test in identifying the premature newborn, within a one-week margin of error, will be the target of the accuracy analysis.

The relationship between the measurement of the newborn's skin reflectance and complications due to pulmonary distress associated with immaturity will be evaluated by means of association tests and risk. The significance level for hypothesis tests will be 5%, together with 95% confidence intervals.

Results

For peer review only - http://bmjopen.bmj.com/site/about/guidelines.xhtml

The study begun with the training of health professionals in September 2018. It is anticipated that the recruitment will take place from January to December 2019. Data analysis will be finalized, the results of which are expected in May 2020.

Discussion

Strengths and Limitations

Availability of trustworthy GA information is a prerequisite for preterm birth classification and healthcare decisions[25]. In this light, the results of this clinical study have the potential to validate a new device for pregnancy dating. The Preemie-Test was prepared to operate with minimum operator intervention and for use by healthcare professionals anywhere a birth takes place without a reliable GA.

The purpose of medical research involving neonates is intended to improve clinical procedures[26]. In this context, a clinical trial is a research study in which subjects are prospectively assigned to intervention and the effects of those interventions on health-related outcomes are thereby evaluated[27]. However, clinical trials on medical devices face barriers when an effective standard procedure does not exist, as is the case of the comparator procedure[28]. Our challenge in preparing the present protocol was the absence of a gold standard for pregnancy dating, since the fetal age begins upon conception; however, this information is difficult to be accurately determined[7].

References

1. Children St. Surviving the first day. In: *State of the world's mothers 2013.* Vol 1. Connecticut: Save the Children; 2013: http://www.savethechildren.org/atf/cf/%7B9def2ebe-10ae-432c-9bd0-df91d2eba74a%7D/SOWM-FULL-REPORT_2013.PDF.

For peer review only - http://bmjopen.bmj.com/site/about/guidelines.xhtml

2. França EB, Lansky S, Rego MAS, et al. Principais causas da mortalidade na infância no Brasil, em 1990 e 2015: estimativas do estudo de Carga Global de Doença. *Revista Brasileira de Epidemiologia.* 2017;20:46-60.

3. Federation StC. HNN website, Healthy Newborn Network. https://www.healthynewbornnetwork.org/. Published 2018. Accessed 01/17/2018, 2018.

4. Howson CP, Kinney MV, McDougall L, Lawn JE, Group BTSPBA. Born too soon: preterm birth matters. *Reprod Health.* 2013;10 Suppl 1:S1.

5. Bhutta ZA, Giuliani F, Haroon A, et al. Standardisation of neonatal clinical practice. *BJOG.* 2013;120 Suppl 2:56-63, v.

6. Liszewski MC, Stanescu AL, Phillips GS, Lee EY. Respiratory Distress in Neonates: Underlying Causes and Current Imaging Assessment. *Radiol Clin North Am.* 2017;55(4):629-644.

7. Committee on Obstetric Practice tAIoUiM, and the Society for Maternal-Fetal Medicine. Committee Opinion No 700: Methods for Estimating the Due Date. *Obstet Gynecol.* 2017;129(5):e150-e154.

8. Karl S, Li Wai Suen CS, Unger HW, et al. Preterm or not--an evaluation of estimates of gestational age in a cohort of women from Rural Papua New Guinea. *PLoS One.* 2015;10(5):e0124286.

9. Wilcox AJ, Weinberg CR, Baird DD. Timing of Sexual Intercourse in Relation to Ovulation — Effects on the Probability of Conception, Survival of the Pregnancy, and Sex of the Baby. *New England Journal of Medicine.* 1995;333(23):1517-1521.

10. Lynch CD, Zhang J. The research implications of the selection of a gestational age estimation method. *Paediatr Perinat Epidemiol.* 2007;21 Suppl 2:86-96.

For peer review only - http://bmjopen.bmj.com/site/about/guidelines.xhtml

11. Ballard JL, Khoury JC, Wedig K, Wang L, Eilers-Walsman BL, Lipp R. New Ballard Score, expanded to include extremely premature infants. *The Journal of Pediatrics.* 1991;119(3):417-423.

12. Lee AC, Panchal P, Folger L, et al. Diagnostic Accuracy of Neonatal Assessment for Gestational Age Determination: A Systematic Review. *Pediatrics.* 2017;140(6).

13. Nguyen TH, Larsen T, Engholm G, Møller H. Increased adverse pregnancy outcomes with unreliable last menstruation. *Obstet Gynecol.* 2000;95(6 Pt 1):867-873.

14. Reis ZSN, Guimarães RN, Inventors. Dispositivo para determinação da idade gestacional, processos e usos. Nov/1/2016, 2016.

15. Reis ZSN, Simeoni U, Vitral GLN, Souza IMFd, Rego MAS, Guimaraes RN. Newborn skin reflection: Proof of concept for a new approach for predicting gestational age at birth. A cross-sectional study. *Plos One.* 2017.

16. Reis ZSN, Vitral GLN, de Souza IMF, Rego MAS, Guimaraes RN. Newborn skin reflection: Proof of concept for a new approach for predicting gestational age at birth. A cross-sectional study. *PLOS ONE.* 2017;12(9):e0184734.

17. Kaplan AV, Baim DS, Smith JJ, et al. Medical device development: from prototype to regulatory approval. *Circulation.* 2004;109(25):3068-3072.

18. Brasil. Resolução da Diretoria Colegiada - RDC No. 10. In: Sanitária AnNdVn, ed. Vol 10. Brasilia: Diário Oficial da União, de 03 de março de 2015; 2015.

19. Papageorghiou AT, Kennedy SH, Salomon LJ, et al. International standards for early fetal size and pregnancy dating based on ultrasound measurement of crown-rump length in the first trimester of pregnancy. *Ultrasound Obstet Gynecol.* 2014;44(6):641-648.

For peer review only

For peer review only - http://bmjopen.bmj.com/site/about/guidelines.xhtml

20. Reuter S, Moser C, Baack M. Respiratory distress in the newborn. *Pediatr Rev.* 2014;35(10):417-428; quiz 429.

21. Faul F, Erdfelder E, Lang A-G, Buchner A. G* Power 3: A flexible statistical power analysis program for the social, behavioral, and biomedical sciences. *Behavior research methods.* 2007;39(2):175-191.

22. Wilson K, Hawken S, Murphy MS, et al. Postnatal Prediction of Gestational Age Using Newborn Fetal Hemoglobin Levels. *EBioMedicine.* 2017;15:203-209.

23. Nielsen J. Usability inspection methods. Conference Companion on Human Factors in Computing Systems; 1994; Boston, Massachusetts, USA.

24. Altman DG BJ. Measurement in medicine: the analysis of method comparison studies. 1983:307-317

25. Beydoun H, Ugwu B, Oehninger S. Assisted reproduction for the validation of gestational age assessment methods. *Reproductive biomedicine online.* 2011;22(4):321-326.

26. WHO. Clinical Trials in Children. Essential medicines for children. World Health Organisation. http://www.who.int/ictrp/child/en/. Accessed 08/01/2018, 2018.

27. USA. NIH's Definition of clinical trial. In: Services USDoHaH, ed. Bethesda: National Institutes of Health; 2018.

28. Neugebauer EAM, Rath A, Antoine SL, et al. Specific barriers to the conduct of randomised clinical trials on medical devices. *Trials.* 2017;18(1):427.

For peer review only

Author statement:

ZSNR: designed the study, planned data collection, prepared the team for good clinical practices, wrote and revised the paper. RNG, RAPLA, MASR, RMCR and JSG made substantial contributions to study design, planned data collection, prepared the team for good clinical practices, wrote and revised the paper. GLNV, MAAR, GSN, PJN, MDRM, and MSV made contributions to standard procedures in methods, drafted the manuscript, reviewed the paper, and approved the final manuscript. EAC: drafted the work and reviewed it critically for important intellectual content, as statistic consultant.

Funding statement

This research was supported by the Grand Challenges Exploration from the Bill & Melinda Gates Foundation (Grant number OPP1128907, Contract), http://www.gatesfoundation.org/, and Fundação de Amparo a Pesquisa de Minas Gerais, Brazil, http://www.fapemig.br/en/, non-profit sectors. The clinical trial is funded by the Brazilian Ministry of Health, Program of Development of the Industrial Health Complex (PROCIS), project 23072.052747/2017-51, trial sponsor contact: Fotini Toscas, e-mail fotini.toscas@saude.gov.br. The funders played no role in the study design, data collection and analysis, decision to publish, or preparation of the manuscript.

Acknowledgements

The authors would like to thank Centre for Clinical Research of Hospital das Clínicas, Universidade Federal de Minas Gerais for general support and Good clinical practices training; Fotini Toscas from the Brazilian Ministry of Health for the active intermediation as the trial sponsor contact.

For peer review only - http://bmjopen.bmj.com/site/about/guidelines.xhtml

Roles and responsibilities: ZSNR is the Principal Investigator and coordinator of the Directive Committee. JSG is the coordinator of the Data Management Team and will continuously receive report adverse events of trial interventions or trial conduct. RAPLA is the coordinator of the Clinical Trial Quality Committee, responsible for important protocol modifications, if necessary.

Conflict of interests statement

Authors declare a patent deposit on behalf of the Universidade Federal de Minas Gerais and Fundação de Amparo a Pesquisa de Minas Gerais, Brazil, http://www.fapemig.br/en/. The inventors were Reis, Zilma Silveira Nogueira and Guimaraes, Rodney Nascimento: BR1020170235688 (CTIT-PN862).

Figure 1. Secondary outcome comparisons between the reference GA and the Preemie-Test in a simulated scenario without best pregnancy dating

Legends: *Gestational age from crown-rump-length data adjusted to Intergrowth's 21st fetal standard[19]. R: reference. GA: gestational age. T: test. C1: comparator 1 is the gestational age calculated using the first ultrasound exam after 13 weeks and 6 days and before 22 weeks of gestation. C2: comparator 2 is the gestational age based on a reliable last menstrual period.

Figure 2. Participant timeline of the study

Legends: GA: gestational age. R: reference. LMP: last menstrual period.

For peer review only - http://bmjopen.bmj.com/site/about/guidelines.xhtml

For peer review only

Word Count: 3415 words

For peer review only

For peer review only - http://bmjopen.bmj.com/site/about/guidelines.xhtml

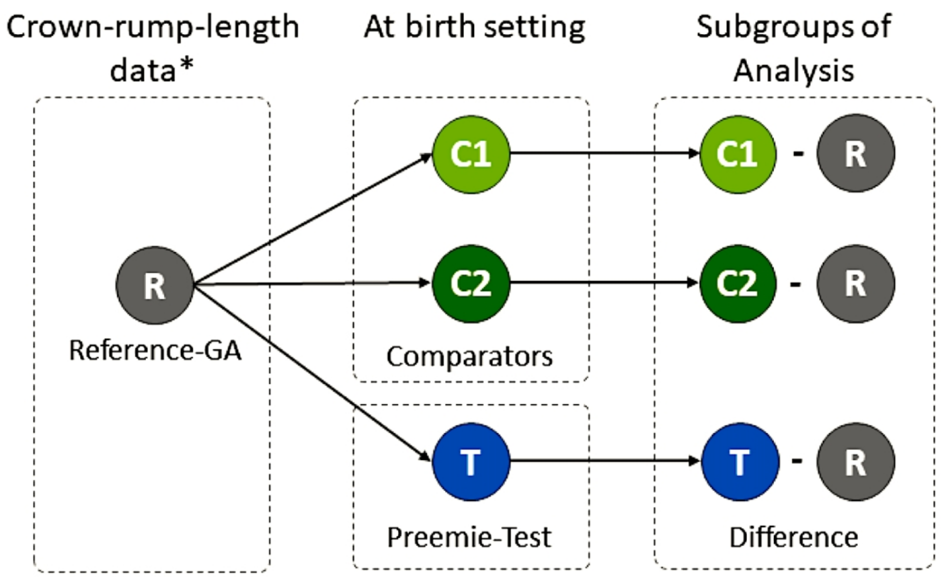

Secondary outcome comparisons between the reference GA and the Preemie-Test in a simulated scenario without best pregnancy dating

Legends: *Gestational age from crown-rump-length data adjusted to Intergrowth's 21st fetal standard19. R: reference. GA: gestational age. T: test. C1: comparator 1 is the gestational age calculated using the first ultrasound exam after 13 weeks and 6 days and before 22 weeks of gestation. C2: comparator 2 is the gestational age based on a reliable last menstrual period.

143x90mm (300 x 300 DPI)

	STUDY PERIOD			
	Enrollment	Assessment	Close-out	Allocation
TIMEPOINT	0	0	72 hours	Analysis
**ENROLLMENT:**				
Eligibility screen	X			
Informed consent	X			
**INTERVENTION:**				
Preemie-Test		X		
**ASSESSMENTS AND ANALYSIS:**				
Preemie-Test: data acquisition		X		
Reference GA: calculated by obstetric ultrasound at <14 weeks of gestation	X			X
Comparator 1: GA calculated by obstetric ultrasound at ≥ 14 and <22 weeks	X			X
Comparator 2: GA calculated by reliable LMP	X			X
Case-control nested study: lung maturity		◆———	———◆	

Participant timeline of the study

Legends: GA: gestational age. R: reference. LMP: last menstrual period.

157x123mm (300 x 300 DPI)

TERMO DE CONSENTIMENTO LIVRE E ESCLARECIDO

Detecção da prematuridade através da interação entre a luz e a pele neonatal: a validação do Preemie-Teste

Sob responsabilidade da pesquisadora Profa Zilma Silveira Nogueira Reis

Cara senhora, você está sendo convidada a participar deste estudo porque acaba de ter um parto no hospital (nome do hospital do centro colaborador) ____.

Apresentação do estudo

O objetivo deste estudo é descobrir novas técnicas para estimar a idade de um bebê ao nascer e identificar aqueles que nasceram antes de nove meses, os prematuros. A idade gestacional desconhecida pode aumentar o risco dos bebês no momento de seu nascimento. As técnicas atuais para se estimar a idade do bebê possuem grande margem de erro.

Acreditamos que a pele possui características que, se bem estudadas, podem refletir a idade das pessoas, e também dos bebês. Por isso, estamos desenvolvendo um novo equipamento médico que se encontra em teste. Ele utiliza a luz para avaliar a composição da pele do bebê e detectar sua idade. Os resultados poderão beneficiar os bebês que nascem sem a informação confiável da idade gestacional.

Instituições envolvidas no estudo

O estudo é desenvolvido pela Faculdade de Medicina da Universidade Federal de Minas Gerais (UFMG), em cooperação com maternidades brasileiras, entre elas a que você se encontra internada. A previsão deste estudo é que 787 crianças recém-nascidas sejam examinadas.

A participação no estudo, riscos e cuidados

Convidamos você e seu bebê para participar deste estudo. Isso incluirá um exame na pele do bebê com a luz, uma breve entrevista com você e a consulta aos registros de saúde sobre a gravidez e os do seu bebê neste hospital. Na entrevista serão tomados todos os cuidados a fim de minimizar os constrangimentos para você. A consulta ao prontuário médico será realizada resguardando o direito de sigilo da informação. Pedimos sua permissão para fotografar a caderneta da gestante ou outro documento equivalente, para conferir a idade gestacional calculada pelos ciclos menstruais e pelos exames de ultrassom. As partes da fotografia que contenham sua identificação serão retiradas da imagem e a manteremos até o final do estudo, quando o arquivo será apagado dos registros da pesquisa.

Pedimos sua permissão para fazer um exame na pele de seu bebê, na região da sola do pé, usando um equipamento em teste. O exame é indolor e externo ao corpo, considerado não-invasivo. A parte que encosta no bebê é pequena e não apresenta pontas que possam ferir a sua pele. Outros equipamentos parecidos, que emitem luz, já são usados nos bebês de forma segura. Por exemplo o oxímetro que faz teste do coraçãozinho. Assim como esse, não se espera que ocorram efeitos imediatos ou futuros na saúde do bebê. Os riscos do teste que faremos incluem a exposição do pé do bebê com perda temporária de calor do corpo e estresse. Cuidados serão tomados a fim de minimizar estes desconfortos. Esclarecemos que o teste dura alguns segundos reduzindo ao mínimo chance de causar marcas ou irritação no local. Caso seu bebê apresente sinais de desconforto durante o exame, o mesmo será interrompido. Você ou familiares poderão permanecer junto ao seu filho durante o exame. Nas crianças que estiverem na Unidade Neonatal, o exame será realizado onde ela já está sendo cuidada, acompanhado pelo profissional de saúde que já está cuidando dela. Caso o seu bebê seja prematuro, todos os devidos cuidados serão tomados antes de cada exame para reduzir a chance de perda de calor, seguindo todas as recomendações de um bebê que fica em incubadora.

Esclarecemos que este estudo não trará benefícios diretos a você ou seu filho, entretanto auxiliará na validação de um novo teste que poderá no futuro identificar o bebê prematuro. Os resultados poderão também gerar informações que ajudem a melhorar os cuidados com outros bebês, quando a idade gestacional é desconhecida. Informamos que os resultados da pesquisa serão publicados em revistas científicas e apresentados em congressos, sem contudo revelar sua identidade ou a do bebê.

Rubrica dos pais: ________________

For peer review only - http://bmjopen.bmj.com/site/about/guidelines.xhtml

As informações obtidas durante a pesquisa serão confidenciais, guardadas em computadores, protegidos por senha e não serão usadas para outros fins. O roubo das informações que coletaremos no estudo é um risco remoto. Para isso, as melhores práticas em segurança de dados serão empregadas. Também poderão ter acesso aos dados da pesquisa o comitê que coordena o estudo, assim como a agência reguladora ANVISA, sem jamais violar a confidencialidade e privacidade dos dados, para que seja possível monitorar se os procedimentos de qualidade e segurança da pesquisa estão sendo seguidos.

Seus direitos como participante

Informamos que a sua participação deve ser voluntária, ou seja, não é obrigatória e caso não concorde ou resolva desistir a qualquer momento isto não trará nenhum constrangimento para você ou para a forma como você será tratada neste hospital. Também não está previsto nenhum tipo de pagamento por sua participação na pesquisa. Este estudo não implica em gastos para você, pois não terá que se deslocar para outro local, permanecer mais tempo no hospital, uma vez que o exame é feito durante sua internação e de seu bebê na maternidade. Caso seja de seu interesse, os resultados do exame que estarão guardados com o pesquisador e lhe serão entregues assim que você solicitar.

Os pesquisadores garantem que acompanharão gratuitamente seu bebê durante a realização do exame e a qualquer momento que se fizer necessário, em qualquer problema que por ventura esteja associado ao estudo ou efeito do teste com a luz.

Este Termo de Consentimento está elaborado em duas vias iguais. Ambas devem ser assinadas por você, pelo pai da criança e pelo pesquisador. Uma via ficará com o participante e a outra com o pesquisador.

O Comitê de Ética em Pesquisa da UFMG pode ser contatado em caso de haver dúvidas quanto aos aspectos éticos da pesquisa, através do telefone (31) 3409-4592 ou endereço completo apresentado a seguir.

Meu nome	
Documento de identidade	
Data de hoje	

Eu declaro que estou em condições de tomar esta decisão e ciente do que foi exposto acima. Autorizo o uso de minhas informações de saúde e as do meu bebê para este projeto de pesquisa, assim como a realização do novo teste. Participo voluntariamente deste estudo e estou ciente que o exame na pele do meu bebê com a luz não traz prejuízo à sua saúde

Assinatura da puérpera:
Assinatura do pai da criança:
Assinatura do pesquisador:

Telefones de contato:
Maternidade Hospital das Clínicas da UFMG – (31) 34099422
Hospital (nome e telefone do hospital colaborador)
Zilma Reis – (31) 985177473 e-mail: skinage.ufmg@gmail.com
Comitê de Ética em Pesquisa da UFMG – Av. Prof. Antônio Carlos, 6627, Unidade Administrativa II, 2° andar, sala 2005, Campus Pampulha, CEP: 31270-901. E-mail:coep@prpq.ufmg.br. Fone (31) 34094592.
Comitê de Ética em Pesquisa do centro colaborador e endereço completo, com e-mail.

For peer review only - http://bmjopen.bmj.com/site/about/guidelines.xhtml

Reporting checklist for protocol of a clinical trial.

Based on the SPIRIT guidelines.

Instructions to authors

Complete this checklist by entering the page numbers from your manuscript where readers will find each of the items listed below.

Your article may not currently address all the items on the checklist. Please modify your text to include the missing information. If you are certain that an item does not apply, please write "n/a" and provide a short explanation.

Upload your completed checklist as an extra file when you submit to a journal.

In your methods section, say that you used the SPIRIT reporting guidelines, and cite them as:

Chan A-W, Tetzlaff JM, Altman DG, Laupacis A, Gøtzsche PC, Krleža-Jerić K, Hróbjartsson A, Mann H, Dickersin K, Berlin J, Doré C, Parulekar W, Summerskill W, Groves T, Schulz K, Sox H, Rockhold FW, Rennie D, Moher D. SPIRIT 2013 Statement: Defining standard protocol items for clinical trials. Ann Intern Med. 2013;158(3):200-207

		Reporting Item	Page Number
Title	#1	Descriptive title identifying the study design, population, interventions, and, if applicable, trial acronym	1
Trial registration	#2a	Trial identifier and registry name. If not yet registered, name of intended registry	2
Trial registration: data set	#2b	All items from the World Health Organization Trial Registration Data Set	2
Protocol version	#3	Date and version identifier	6
Funding	#4	Sources and types of financial, material, and other support	18
Roles and responsibilities: contributorship	#5a	Names, affiliations, and roles of protocol contributors	19

For peer review only

Roles and responsibilities: sponsor contact information	#5b	Name and contact information for the trial sponsor	19
Roles and responsibilities: sponsor and funder	#5c	Role of study sponsor and funders, if any, in study design; collection, management, analysis, and interpretation of data; writing of the report; and the decision to submit the report for publication, including whether they will have ultimate authority over any of these activities	18
Roles and responsibilities: committees	#5d	Composition, roles, and responsibilities of the coordinating centre, steering committee, endpoint adjudication committee, data management team, and other individuals or groups overseeing the trial, if applicable (see Item 21a for data monitoring committee)	18
Background and rationale	#6a	Description of research question and justification for undertaking the trial, including summary of relevant studies (published and unpublished) examining benefits and harms for each intervention	4
Background and rationale: choice of comparators	#6b	Explanation for choice of comparators	4
Objectives	#7	Specific objectives or hypotheses	5
Trial design	#8	Description of trial design including type of trial (eg, parallel group, crossover, factorial, single group), allocation ratio, and framework (eg, superiority, equivalence, non-inferiority, exploratory)	6
Study setting	#9	Description of study settings (eg, community clinic, academic hospital) and list of countries where data will be collected. Reference to where list of study sites can be obtained	6
Eligibility criteria	#10	Inclusion and exclusion criteria for participants. If applicable, eligibility criteria for study centres and	7

For peer review only

		individuals who will perform the interventions (eg, surgeons, psychotherapists)	
Interventions: description	#11a	Interventions for each group with sufficient detail to allow replication, including how and when they will be administered	7-8
Interventions: modifications	#11b	Criteria for discontinuing or modifying allocated interventions for a given trial participant (eg, drug dose change in response to harms, participant request, or improving / worsening disease)	9
Interventions: adherance	#11c	Strategies to improve adherence to intervention protocols, and any procedures for monitoring adherence (eg, drug tablet return; laboratory tests)	NA
Interventions: concomitant care	#11d	Relevant concomitant care and interventions that are permitted or prohibited during the trial	9
Outcomes	#12	Primary, secondary, and other outcomes, including the specific measurement variable (eg, systolic blood pressure), analysis metric (eg, change from baseline, final value, time to event), method of aggregation (eg, median, proportion), and time point for each outcome. Explanation of the clinical relevance of chosen efficacy and harm outcomes is strongly recommended	9-10
Participant timeline	#13	Time schedule of enrolment, interventions (including any run-ins and washouts), assessments, and visits for participants. A schematic diagram is highly recommended (see Figure)	11
Sample size	#14	Estimated number of participants needed to achieve study objectives and how it was determined, including clinical and statistical assumptions supporting any sample size calculations	11-12
Recruitment	#15	Strategies for achieving adequate participant enrolment to reach target sample size	7

For peer review only

Allocation: sequence generation	#16a	Method of generating the allocation sequence (eg, computer-generated random numbers), and list of any factors for stratification. To reduce predictability of a random sequence, details of any planned restriction (eg, blocking) should be provided in a separate document that is unavailable to those who enrol participants or assign interventions	NA
Allocation concealment mechanism	#16b	Mechanism of implementing the allocation sequence (eg, central telephone; sequentially numbered, opaque, sealed envelopes), describing any steps to conceal the sequence until interventions are assigned	NA
Allocation: implementation	#16c	Who will generate the allocation sequence, who will enrol participants, and who will assign participants to interventions	NA
Blinding (masking)	#17a	Who will be blinded after assignment to interventions (eg, trial participants, care providers, outcome assessors, data analysts), and how	8
Blinding (masking): emergency unblinding	#17b	If blinded, circumstances under which unblinding is permissible, and procedure for revealing a participant's allocated intervention during the trial	NA
Data collection plan	#18a	Plans for assessment and collection of outcome, baseline, and other trial data, including any related processes to promote data quality (eg, duplicate measurements, training of assessors) and a description of study instruments (eg, questionnaires, laboratory tests) along with their reliability and validity, if known. Reference to where data collection forms can be found, if not in the protocol	12
Data collection plan: retention	#18b	Plans to promote participant retention and complete follow-up, including list of any outcome data to be collected for participants who discontinue or deviate from intervention protocols	12
Data management	#19	Plans for data entry, coding, security, and storage, including any related processes to promote data quality	12

For peer review only

		7(eg, double data entry; range checks for data values). Reference to where details of data management procedures can be found, if not in the protocol	
Statistics: outcomes	#20a	Statistical methods for analysing primary and secondary outcomes. Reference to where other details of the statistical analysis plan can be found, if not in the protocol	12
Statistics: additional analyses	#20b	Methods for any additional analyses (eg, subgroup and adjusted analyses)	12
Statistics: analysis population and missing data	#20c	Definition of analysis population relating to protocol non-adherence (eg, as randomised analysis), and any statistical methods to handle missing data (eg, multiple imputation)	NA
Data monitoring: formal committee	#21a	Composition of data monitoring committee (DMC); summary of its role and reporting structure; statement of whether it is independent from the sponsor and competing interests; and reference to where further details about its charter can be found, if not in the protocol. Alternatively, an explanation of why a DMC is not needed	18
Data monitoring: interim analysis	#21b	Description of any interim analyses and stopping guidelines, including who will have access to these interim results and make the final decision to terminate the trial	NA
Harms	#22	Plans for collecting, assessing, reporting, and managing solicited and spontaneously reported adverse events and other unintended effects of trial interventions or trial conduct	19
Auditing	#23	Frequency and procedures for auditing trial conduct, if any, and whether the process will be independent from investigators and the sponsor	12
Research ethics approval	#24	Plans for seeking research ethics committee / institutional review board (REC / IRB) approval	6

For peer review only

Protocol amendments	#25	Plans for communicating important protocol modifications (eg, changes to eligibility criteria, outcomes, analyses) to relevant parties (eg, investigators, REC / IRBs, trial participants, trial registries, journals, regulators)	19
Consent or assent	#26a	Who will obtain informed consent or assent from potential trial participants or authorised surrogates, and how (see Item 32)	6
Consent or assent: ancillary studies	#26b	Additional consent provisions for collection and use of participant data and biological specimens in ancillary studies, if applicable	NA
Confidentiality	#27	How personal information about potential and enrolled participants will be collected, shared, and maintained in order to protect confidentiality before, during, and after the trial	6
Declaration of interests	#28	Financial and other competing interests for principal investigators for the overall trial and each study site	18
Data access	#29	Statement of who will have access to the final trial dataset, and disclosure of contractual agreements that limit such access for investigators	7
Ancillary and post trial care	#30	Provisions, if any, for ancillary and post-trial care, and for compensation to those who suffer harm from trial participation	NA
Dissemination policy: trial results	#31a	Plans for investigators and sponsor to communicate trial results to participants, healthcare professionals, the public, and other relevant groups (eg, via publication, reporting in results databases, or other data sharing arrangements), including any publication restrictions	7
Dissemination policy: authorship	#31b	Authorship eligibility guidelines and any intended use of professional writers	NA
Dissemination policy: reproducible research	#31c	Plans, if any, for granting public access to the full protocol, participant-level dataset, and statistical code	7

For peer review only

Informed consent materials	#32	Model consent form and other related documentation given to participants and authorised surrogates	6
Biological specimens	#33	Plans for collection, laboratory evaluation, and storage of biological specimens for genetic or molecular analysis in the current trial and for future use in ancillary studies, if applicable	NA

The SPIRIT checklist is distributed under the terms of the Creative Commons Attribution License CC-BY-ND 3.0. This checklist can be completed online using https://www.goodreports.org/, a tool made by the EQUATOR Network in collaboration with Penelope.ai

For peer review only

BMJ Open

Prematurity detection evaluating interaction between the skin of the newborn and light: Protocol for the Preemie-Test multicenter clinical trial in Brazilians hospitals to validate a new medical device

Journal:	*BMJ Open*
Manuscript ID	bmjopen-2018-027442.R2
Article Type:	Protocol
Date Submitted by the Author:	03-Dec-2018

Complete List of Authors:	Reis, Zilma; Universidade Federal de Minas Gerais, Gynecology and Obstetrics<br>Guimarães, Rodney; Faculty of Medicine, Universidade Federal de Minas Gerais, Ginecologia e Obstetrícia<br>Rego, Maria Albertina; Faculty of Medicine, Universidade Federal de Minas Gerais, Pediatrics<br>Maia de Castro Romanelli , Roberta ; Universidade Federal de Minas Gerais Faculdade de Medicina,<br>Gaspar, Juliano; Faculty of Medicine, Universidade Federal de Minas Gerais, Obstetrics and Gynecology<br>Vitral, Gabriela; Faculty of Medicine, Universidade Federal de Minas Gerais<br>dos Reis, Marconi; Faculty of Medicine, Universidade Federal de Minas Gerais, Pediatrics<br>Colósimo, Enrico; Universidade Federal de Minas Gerais, Statistics<br>Neves, Gabriela; Hospital Sofia Feldman<br>Vale, Marynea; Hospital Universitario da Universidade Federal do Maranhao, Pediatrics<br>Nader, Paulo; Universidade Luterana do Brasil. Hospital Universitário de Canoas, Pediatrics<br>de Moura, Martha; Hospital Materno Infantil de Brasília<br>de Aguiar, Regina ; Faculty of Medicine, Universidade Federal de Minas Gerais, Obstetrics and Gynecology

**Primary Subject Heading**:	Obstetrics and gynaecology
Secondary Subject Heading:	Global health, Diagnostics, Paediatrics
Keywords:	Gestational Age, Infant, Premature, Skin Physiological Phenomena, Photomedicine, Equipment and Supplies

SCHOLARONE™ Manuscripts

Prematurity detection evaluating interaction between the skin of the newborn and light: Protocol for the Preemie-Test multicenter clinical trial in Brazilians hospitals to validate a new medical device

Zilma Silveira Nogueira Reis[1]*; Rodney Nascimento Guimarães[1]; Maria Albertina Santiago Rego[1]; Roberta Maia de Castro Romanelli[1]; Juliano de Souza Gaspar[1]; Gabriela Luíza Nogueira Vitral[1]; Marconi Augusto Aguiar dos Reis[1]; Enrico Antônio Colósimo[2]; Gabriela Silveira Neves[3]; Marynea Silva do Vale[4]; Paulo de Jesus Nader[5]; Martha David Rocha de Moura[6]; Regina Amélia Pessoa Lopes de Aguiar[1]

1 - Faculty of Medicine, Universidade Federal de Minas Gerais, Brazil.

2 - Statistics Department, Universidade Federal de Minas Gerais, Brazil.

3 - Hospital Sofia Feldman, Brazil.

4 - Hospital Universitário da Universidade Federal do Maranhão, Brazil.

5 - Universidade Luterana do Brasil. Hospital Universitário de Canoas, Brazil.

6 - Hospital Materno Infantil de Brasília, Brazil.

*Corresponding author: Zilma Silveira Nogueira Reis. Avenida Professor Alfredo Balena, 190, sala 601. Zip Code 30.130.100 Belo Horizonte, Brazil. Phone: +55 0 31 3409-9467. Email: zilma.medicina@gmail.com

Abstract

For peer review only - http://bmjopen.bmj.com/site/about/guidelines.xhtml

Introduction: Recognizing prematurity is critical in order to attend to immediate needs in childbirth settings, guiding the extent of medical care provided for newborns. A new medical device has been developed to carry out the Preemie-Test, an innovative approach to estimate gestational age (GA), based on the photobiological properties of the newborn's skin. This study will validate the Preemie-Test for GA estimation at birth and its accuracy to detect prematurity. Secondarily, the study intends to associate the infant´s skin reflectance with lung maturity, as well as evaluate safety, precision, and usability of a new medical device to offer a suitable product for health professionals during childbirth and in neonatal care settings.

Methods and analysis: Research protocol for diagnosis, single-group, single-blinding, and single-arm multicenter clinical trials with a reference standard. Alive newborns, with 24 weeks or more of pregnancy age, will be enrolled during the first 24 hours of life. Sample size is 787 subjects. The primary outcome is the difference between the GA calculated by the photobiological neonatal skin assessment methodology and the GA calculated by the comparator antenatal ultrasound or reliable last menstrual period. Immediate complications caused by pulmonary immaturity during the first 72 hours of life will be associated with skin reflectance in a nested case-control study.

Ethics and dissemination: Each local independent ethics review board approved the trial protocol. The authors intend to share the minimal anonymized data set necessary to replicate study findings.

Trial registration number: WHO Clinical Trial RBR-3f5bm5.

Key-words: Gestational Age, Infant, Premature; Skin Physiological Phenomena; Photomedicine; Equipment and Supplies.

For peer review only - http://bmjopen.bmj.com/site/about/guidelines.xhtml

Article Summary

Strengths and limitations of this study:

- The study will provide high-quality data on prematurity detection, based on the newborn's skin assessment, using a photometer device.
- The gold standard comparator for pregnancy dating does not exist; instead a reference standard will be used with blinded primary outcome.
- The agreement endpoint between methods for gestational age determination precludes randomization of the intervention.

For peer review only

Introduction

In childbirth settings, health professionals continuously need to make timely decisions to provide proper neonatal care. The day of birth is the riskiest for newborns and mothers almost everywhere[1]. Perinatal causes related to prematurity and complications during childbirth, which are generally preventable through qualified health care, are the primary causes of death among newborns[1,2]. Most of these deaths took place in countries with low resources and a scarcity of health facilities[3]. The opportune recognition of prematurity is critical in order to judge the viability of the newborn and to attend to his/her immediate needs, guiding the complexity of the medical care provided for the newborn. Without reliable information on the age of the unborn phase, actions to preserve the potential for survival of the newborn can be neglected[4]. Indeed, the attempted management of the risk of mortality and severe complications are sensitive issues to the gestational age (GA), which involves temperature maintenance, ventilatory support, transport to a neonatal intensive care unit (NICU), and the early treatment of respiratory distress syndrome (RDS), the most severe complication of premature birth[5]. In addition to the GA information or birthweight, the prediction of neonatal respiratory morbidity may be critical in planning immediate medical care[6], since the respiratory system is among the last of the fetal organ systems to mature, which is associated with enhanced morbidity and mortality[6].

Current methods of dating pregnancy remain a worldwide challenge. Early obstetric ultrasound currently offers the best due date[7]. However, access to this type of exam is limited because of high equipment costs, poor training and skills of health professionals, or late prenatal care[8]. Despite a 10-days or more margin of error during the second and third trimester of gestation, ultrasound is still a reasonable methodology for GA determination, when the best opportunity was lost[7]. The calculation, based on the historical information of the last menstrual period (LMP), is

For peer review only - http://bmjopen.bmj.com/site/about/guidelines.xhtml

impacted by the uncertainty of both the fertility days and date of conception[9], due to the bias of memory, the use of hormonal contraception, and breastfeeding[10]. After birth, neurological scores, such as the New Ballard[11], show a tendency to overestimate GA in preterm infants and underestimate GA in growth-restricted infants[12]. Efforts to enhance the reliability of pregnancy dating, through more accurate and accessible technologies, seek to improve pregnancy outcomes and neonatal survival[13].

A new medical device has been developed to carry out the Preemie-Test, an innovative approach used to estimate GA, based on the photobiological properties of the newborn's skin. This reflective test is noninvasive, and the device automatically processes the light, scattered by the constituents of the skin layers, when a small optoelectronic light emitter/receiver sensor touches the newborn’s skin[14]. The device under test is easy to use and every effort is being made to ensure that it has excellent accuracy, be it safe and low cost. The feasibility study provided a mathematical model to predict GA based on the skin reflectance adjusted to clinical variables (R^2 = 0.828, P <0.001)[15]. However, before the adoption or use of an innovation, an effectiveness trial of intervention is a critical step in the research chain regarding its the social utility when completing the translation from the proof of concept to clinical science[16]. The rationale for the main hypothesis in this study is that the skin maturity of a newborn, obtained by the analysis of its optical properties, is useful in pregnancy dating for clinical use and respiratory prognosis, especially in a scenario with no reliable GA based on current methods. This study aims to validate the photobiological model of the skin, called the “Preemie-test”, in order to estimate GA at birth and determine its accuracy in detecting prematurity. Secondarily, it also seeks to associate the infant’s skin reflectance with lung maturity. Moreover, this study intends to evaluate the safety, precision, and the usability of a new medical device to offer a suitable product to support health professionals during childbirth and in neonatal care settings.

For peer review only - http://bmjopen.bmj.com/site/about/guidelines.xhtml

Methods

Study design

This study will use a protocol for diagnosis, single-group, single-blinding, and single-arm multicenter clinical trials with a reference standard. This new photobiological approach to the skin, gathered in a medical device, is currently in the pivotal phase of innovation development from the prototype to regulatory approval[17]. This step aims to provide the translation[16] of the scientific model for GA detection based on skin maturity. This Protocol version is 1, July/10th/2018. Faculty of Medicine, Universidade Federal de Minas Gerais is the Coordinator Center.

Study Settings, Ethics and Dissemination

Selected Brazilian referral centers for high-risk pregnancy and neonatal care will participate in the study, according to this protocol: Hospital das Clínicas, Universidade Federal de Minas Gerais, as the Center for Coordination; Hospital Sofia Feldman, Minas Gerais State; Hospital da Universidade Luterana do Brasil, Rio Grande do Sul State; Hospital Materno-infantil de Brasília, Distrito Federal; and Hospital Universitário da Universidade Federal do Maranhão, Maranhão State. Each local independent ethics review board approved the trial protocol, and the Brazilian National Research Council (CONEP) approved all study activities and protocol prior to the commencement of study activities, in accordance with the Declaration of Helsinki (2008), good clinical practice as set forth by the International Organization for Standardization (ISO) 14155:2011, and the Brazilian regulatory health agency's recommendations[18]. This study was logged under both protocol number CAAE 81347817.6.1001.5149 and the International Clinical Trials Registry Platform under number RBR-3f5bm5. Parents will sign an informed consent form on behalf of the newborn before participating in the clinical trial (supplementary file).

For peer review only - http://bmjopen.bmj.com/site/about/guidelines.xhtml

Data Sharing Statement

The authors intend to share the minimal anonymized data set necessary to replicate study findings. Data sharing will include: the reference and comparators GA, GA estimated by the Preemie-test, birth weight, RDS or transient tachypnea of the newborn (TTN) diagnosis, ventilatory support due to pulmonary immaturity, neonatal intensive care unit (NICU) admission due to RDS or TTN, and any adverse events regarding device's safety. Unidentified data and study-related documents as ethical approvals will be accessible by URLs for researchers, regulatory agencies, and sponsors.

Patient and Public Involvement

Patients and the public were not involved in the design of this study. The results will be disseminated to study parents of participants through scientific publications, non-scientific publications, and on the website of the project: http://skinage.medicina.ufmg.br.

Eligibility criteria and participant's timeline

A prospective sequential and concurrent enrollment process will select newborns in referral hospitals centers for neonatal care. Infants are eligible with the following inclusion criteria: (1) alive newborn; (2) enrollment during first 24 hours of life; (3) be 24 weeks or more of gestational age, at birth; (4) fetus underwent an obstetric ultrasound assessment before 14 weeks of pregnancy; (5) fetus also had obstetric ultrasound assessment between 14 and 22 gestational weeks. Exclusion criteria are: (1) malformation with structural skin alterations; (2) skin modifiers: anhydramnios, hydrops, congenital skin diseases or chorioamnionitis. Randomisation was not appropriate to assess the agreement between different methods to assess pregnancy dating.

In a nested case-control study, we will select newborns within the first 72 hours of life,

For peer review only - http://bmjopen.bmj.com/site/about/guidelines.xhtml

discharge, or death, whichever occurs first, with the following inclusion criteria: (1) RDS or (2) TTN diagnosis. Ranges of gestational age will randomly pair controls. Exclusion criteria include: (1) the existence of extra pulmonary conditions with tachypnea not due to prematurity and (2) diagnosis of Clinical or Laboratory-Confirmed Bloodstream Infection.

Intervention: The Preemie-Test

The Preemie-Test assessment occurs as soon as possible after birth, in the first 24 hours, inside incubators, open heating crib, common crib or in the mother's lap, in order to ensure minimum manipulation and stable clinical conditions. The acquisitions of all newborns will be stored in a database for further statistical analysis.

A noninvasive, handheld optoelectronic prototype has been developed to measure the backscattered light signal from the skin[15]. The equipment regulates the emitted light and processes the received light signal in the sensor, resulting in the prediction of GA by a mathematical model, associated or not with clinical variables. According to the Brazilian regulatory health agency (ANVISA), this medical device is categorized as a Class II safety: noninvasive and medium risk. The prototype unit of measurement and the process of GA estimation were patented under number BR1020170235688 (CTIT-PN862)[14]. An updated version of the invention received improvements in order to safeguard reliability and to minimize examiner interferences on the skin's backscattering acquisition. The light emitting-sensor touches the skin over the sole of the foot for a few seconds. The skin reflectance will be sensed once the light has been emitted by a light emitting diode (LED) at wavelengths from 400 nm to 1200 nm. Data acquisitions occur automatically, without operator influence, and are obtained three times per newborn, in the same site and sequentially. Digital recordings will be uploaded to a server for further analysis. The prototype will blind the examiner to the predicted GA value.

For peer review only - http://bmjopen.bmj.com/site/about/guidelines.xhtml

The criterium for discontinuing the interventions for a given trial participant will be in case of parents of the newborns' request.

Training and monitoring

Systematic monitoring of data collection, through an electronic information system, would trigger any adverse event. This medical team is still responsible for the training of healthcare professionals to recruit participants, data collection, a safely performed Preemie-Test during the newborn's assessment, and the monitoring of data quality. The certification of co-participant centers involved the accomplishment of at least 30 simulated examinations by the participant health professionals in the study.

Gestational age methods of calculation and comparators

Reference-GA (R) is calculated upon enrollment, using the embryo measurement assessed by ultrasound exam at <14 weeks of gestation as a reference. Crown-rump-length (CRL) data, recorded from the ultrasound report or prenatal care book-document, will be considered the crude data, when available. Intergrowth's 21st standard curve for ultrasound measurements from 7 weeks and 3 days up to 13 weeks and 6 days will be adjusted to all GA data, according to CRL[19].

GA methods to calculate GA in the childbirth setting, and their comparators are as follows:

- Preemie-Test-GA (T): data statistically determined by analyzing the acquired information stored in the device's processor.
- Comparators-GA (C): calculated using the first ultrasound exam after 13 weeks and 6 days of gestation and before 22 weeks (C1). When available, a second comparator is GA based on a reliable LMP (C2)[13].

For peer review only - http://bmjopen.bmj.com/site/about/guidelines.xhtml

We will take a scanning copy of the prenatal care book or the ultrasound report. After evaluating the data quality, the images will be discarded. To achieve a reliable LMP, we will interview the woman, as suggested by Nguyen et al. (2000)[13].

Primary outcome measures

The primary target is the agreement between the GA offered by the Preemie-Test (T) and the GA calculated by the comparators (C1 and C2), so as to perform the new test in scenarios without the Reference-GA (R). The outcome is the difference between the GA calculated by the photobiological neonatal skin assessment methodology in relation to the age calculated by the comparators.

Another measure for the primary target is the detection of preterm newborns, considering the age before 37 weeks of pregnancy as the threshold between term and preterm births, and analyzing sub-categories of preterm birth, based on GA[4]:

- extremely preterm (less than 28 weeks)
- very preterm (28 to 32 weeks)
- moderate to late preterm (more than 32 to less than 37 weeks).

In this case, the outcome is the proportion of the preterm newborn correctly detected at birth, based on the photobiological test of the skin, within a one-week error.

Secondary outcome measures

1. In a simulated scenario, in which the Reference-GA (R) is unknown, two groups will be randomly assigned from the complete database in order to compare differences among the Reference-GA (R), the GA obtained through the Preemie-Test (T), and the GA calculated by the comparators. Figure 1 presents such subgroups and measures for comparison.

For peer review only - http://bmjopen.bmj.com/site/about/guidelines.xhtml

2. To monitor the device's safety when in regular use by participants over a 72-hour period. Adverse events will be monitored, according to ISO 14155:2011 standards. This means any unexpected medical events, unintended disease or injury, or unfortunate clinical signs in subjects, users, or other people, whether related to the investigational medical device or not.
3. To establish the *ease of use* of the Preemie-Test measurement as a potential method for preterm newborn diagnosis.

The secondary outcome measures in the case-control nested study

Immediate complications, occurring during the first 72 hours of life due to pulmonary immaturity, are the secondary target. The outcome measures are as follows:

- To describe the relationship of the measurement of the newborn's skin reflectance with RDS and with diagnoses based on clinical and radiological findings and respiratory outcomes[6,20].
- To describe the relationship of the measurement of the newborn's skin reflectance with the TTN and with diagnoses based on clinical findings and respiratory outcomes[6].
- To describe the relationship of the measurement of the newborn's skin reflectance with ventilatory support due to pulmonary immaturity.
- To describe the relationship of the measurement of the newborn's skin reflectance with NICU admission due to RDS or TTN.

Time schedule of enrollment, intervention, and outcome measurements are presented in a schematic diagram (see Figure 2). The assessment occurs during the first 24 hours of life, but participants will be followed up for 72 hours or until discharge or death, whichever occurs first, for the monitoring of neonatal outcomes and adverse events.

Sampling and sample size

For peer review only - http://bmjopen.bmj.com/site/about/guidelines.xhtml

The sample size calculation is estimated based on the primary endpoint. To test the hypothesis of equivalence between the Preemie-Test GA and the comparators GA, a sample of 787 subjects is necessary to detect an effect size of 10%. Using the G-Power 3.1 software[21], we assumed an alpha error of 0.05, and a power of test of 0.80 to support a paired t-test.

Sampling intends to arrange three groups of GA enrollment to preserve enough premature newborns with 3:2:1 proportion, similar to Wilson et al. (2017)[22]: 392 term newborns, 263 premature newborns from 32 to 36 weeks and six days of GA, and 132 extremely premature newborns from 24 to 31 weeks and six days of GA.

Usability

The usability assessment will be performed by applying a checklist to participants who use the prototype device to perform the Preemie-Test. The 10 heuristics proposed by Nielsen and Marck (1994)[23] will be adapted to build a checklist to evaluate the device, namely: (a) system visibility, (b) correspondence with the real world, (c) user control and freedom, (d) consistency of results and standardization, (e) error prevention, (f) visual recognition rather than memorization, (g) flexibility and efficiency of use, (h) esthetic and minimalist design, (i) help for the user to recognize, diagnose, and recover from errors, and (j) user documentation and help.

Data collection

Standard operational procedures set data entries in structured questionaries. In this concurrent clinical trial, an electronic information system was developed to collect data in different hospitals, simultaneously. Entry forms validations were implemented with data values ranges to ensure the quality of the information. An audit of the data will be permanently performed and the data summary available on the project webpage. Double system, paper-based and electronic will permit audit concerning reliability and

For peer review only

validity. Independent rater over-read all papers files and cross check with the electronic information from all patients.

Data analysis

Demographics and baseline characteristics of the study group, as well the intervention measurements, will be summarized by the frequencies and the mean and standard deviation (SD), the whereas median and interquartile range will be preferred for non-normally distributed continuous variables.

To model the GA prediction, computational randomization will select two subsamples in the database. One of them to train the prediction model of GA based on skin reflectance and clinical variables, such as sex, time in an incubator, phototherapy, birth weight, among others. Another part will be for the analytical validation of the predictive model. Improvements in the existing prediction models for GA (Preemie-Test), will be conducted with conventional statistical and data mining analyses.

Regarding the primary endpoint, the agreement among three methods for GA will be calculated using the Intraclass coefficient correlation and Bland & Altman plots[24], and paired t-testing. The accuracy of the Preemie-Test in identifying the premature newborn, within a one-week margin of error, will be the target of the accuracy analysis.

The relationship between the measurement of the newborn's skin reflectance and complications due to pulmonary distress associated with immaturity will be evaluated by means of association tests and risk. The significance level for hypothesis tests will be 5%, together with 95% confidence intervals.

Results

For peer review only - http://bmjopen.bmj.com/site/about/guidelines.xhtml

The study begun with the training of health professionals in September 2018. It is anticipated that the recruitment will take place from January to December 2019. Data analysis will be finalized, the results of which are expected in May 2020.

Discussion

Strengths and Limitations

Availability of trustworthy GA information is a prerequisite for preterm birth classification and healthcare decisions[25]. In this light, the results of this clinical study have the potential to validate a new device for pregnancy dating. The Preemie-Test was prepared to operate with minimum operator intervention and for use by healthcare professionals anywhere a birth takes place without a reliable GA.

The purpose of medical research involving neonates is intended to improve clinical procedures[26]. In this context, a clinical trial is a research study in which subjects are prospectively assigned to intervention and the effects of those interventions on health-related outcomes are thereby evaluated[27]. However, clinical trials on medical devices face barriers when an effective standard procedure does not exist, as is the case of the comparator procedure[28]. Our challenge in preparing the present protocol was the absence of a gold standard for pregnancy dating, since the fetal age begins upon conception; however, this information is difficult to be accurately determined[7].

References

1. Children St. Surviving the first day. In: *State of the world's mothers 2013.* Vol 1. Connecticut: Save the Children; 2013: http://www.savethechildren.org/atf/cf/%7B9def2ebe-10ae-432c-9bd0-df91d2eba74a%7D/SOWM-FULL-REPORT_2013.PDF.

For peer review only - http://bmjopen.bmj.com/site/about/guidelines.xhtml

2. França EB, Lansky S, Rego MAS, et al. Principais causas da mortalidade na infância no Brasil, em 1990 e 2015: estimativas do estudo de Carga Global de Doença. *Revista Brasileira de Epidemiologia.* 2017;20:46-60.

3. Federation StC. HNN website, Healthy Newborn Network. https://www.healthynewbornnetwork.org/. Published 2018. Accessed 01/17/2018, 2018.

4. Howson CP, Kinney MV, McDougall L, Lawn JE, Group BTSPBA. Born too soon: preterm birth matters. *Reprod Health.* 2013;10 Suppl 1:S1.

5. Bhutta ZA, Giuliani F, Haroon A, et al. Standardisation of neonatal clinical practice. *BJOG.* 2013;120 Suppl 2:56-63, v.

6. Liszewski MC, Stanescu AL, Phillips GS, Lee EY. Respiratory Distress in Neonates: Underlying Causes and Current Imaging Assessment. *Radiol Clin North Am.* 2017;55(4):629-644.

7. Committee on Obstetric Practice tAIoUiM, and the Society for Maternal-Fetal Medicine. Committee Opinion No 700: Methods for Estimating the Due Date. *Obstet Gynecol.* 2017;129(5):e150-e154.

8. Karl S, Li Wai Suen CS, Unger HW, et al. Preterm or not--an evaluation of estimates of gestational age in a cohort of women from Rural Papua New Guinea. *PLoS One.* 2015;10(5):e0124286.

9. Wilcox AJ, Weinberg CR, Baird DD. Timing of Sexual Intercourse in Relation to Ovulation — Effects on the Probability of Conception, Survival of the Pregnancy, and Sex of the Baby. *New England Journal of Medicine.* 1995;333(23):1517-1521.

For peer review only

10. Lynch CD, Zhang J. The research implications of the selection of a gestational age estimation method. *Paediatr Perinat Epidemiol.* 2007;21 Suppl 2:86-96.

11. Ballard JL, Khoury JC, Wedig K, Wang L, Eilers-Walsman BL, Lipp R. New Ballard Score, expanded to include extremely premature infants. *The Journal of Pediatrics.* 1991;119(3):417-423.

12. Lee AC, Panchal P, Folger L, et al. Diagnostic Accuracy of Neonatal Assessment for Gestational Age Determination: A Systematic Review. *Pediatrics.* 2017;140(6).

13. Nguyen TH, Larsen T, Engholm G, Møller H. Increased adverse pregnancy outcomes with unreliable last menstruation. *Obstet Gynecol.* 2000;95(6 Pt 1):867-873.

14. Reis ZSN, Guimarães RN, Inventors. Dispositivo para determinação da idade gestacional, processos e usos. Nov/1/2016, 2016.

15. Reis ZSN, Simeoni U, Vitral GLN, Souza IMFd, Rego MAS, Guimaraes RN. Newborn skin reflection: Proof of concept for a new approach for predicting gestational age at birth. A cross-sectional study. *Plos One.* 2017.

16. Reis ZSN, Vitral GLN, de Souza IMF, Rego MAS, Guimaraes RN. Newborn skin reflection: Proof of concept for a new approach for predicting gestational age at birth. A cross-sectional study. *PLOS ONE.* 2017;12(9):e0184734.

17. Kaplan AV, Baim DS, Smith JJ, et al. Medical device development: from prototype to regulatory approval. *Circulation.* 2004;109(25):3068-3072.

18. Brasil. Resolução da Diretoria Colegiada - RDC No. 10. In: Sanitária AnNdVn, ed. Vol 10. Brasilia: Diário Oficial da União, de 03 de março de 2015; 2015.

For peer review only - http://bmjopen.bmj.com/site/about/guidelines.xhtml

19. Papageorghiou AT, Kennedy SH, Salomon LJ, et al. International standards for early fetal size and pregnancy dating based on ultrasound measurement of crown-rump length in the first trimester of pregnancy. *Ultrasound Obstet Gynecol.* 2014;44(6):641-648.

20. Reuter S, Moser C, Baack M. Respiratory distress in the newborn. *Pediatr Rev.* 2014;35(10):417-428; quiz 429.

21. Faul F, Erdfelder E, Lang A-G, Buchner A. G* Power 3: A flexible statistical power analysis program for the social, behavioral, and biomedical sciences. *Behavior research methods.* 2007;39(2):175-191.

22. Wilson K, Hawken S, Murphy MS, et al. Postnatal Prediction of Gestational Age Using Newborn Fetal Hemoglobin Levels. *EBioMedicine.* 2017;15:203-209.

23. Nielsen J. Usability inspection methods. Conference Companion on Human Factors in Computing Systems; 1994; Boston, Massachusetts, USA.

24. Altman DG BJ. Measurement in medicine: the analysis of method comparison studies. 1983:307-317

25. Beydoun H, Ugwu B, Oehninger S. Assisted reproduction for the validation of gestational age assessment methods. *Reproductive biomedicine online.* 2011;22(4):321-326.

26. WHO. Clinical Trials in Children. Essential medicines for children. World Health Organisation. http://www.who.int/ictrp/child/en/. Accessed 08/01/2018, 2018.

27. USA. NIH's Definition of clinical trial. In: Services USDoHaH, ed. Bethesda: National Institutes of Health; 2018.

For peer review only - http://bmjopen.bmj.com/site/about/guidelines.xhtml

28. Neugebauer EAM, Rath A, Antoine SL, et al. Specific barriers to the conduct of randomised clinical trials on medical devices. *Trials.* 2017;18(1):427.

Author statement:

ZSNR: designed the study, planned data collection, prepared the team for good clinical practices, wrote and revised the paper. RNG, RAPLA, MASR, RMCR and JSG made substantial contributions to study design, planned data collection, prepared the team for good clinical practices, wrote and revised the paper. GLNV, MAAR, GSN, PJN, MDRM, and MSV made contributions to standard procedures in methods, drafted the manuscript, reviewed the paper, and approved the final manuscript. EAC: drafted the work and reviewed it critically for important intellectual content, as statistic consultant.

Funding statement

This research was supported by the Grand Challenges Exploration from the Bill & Melinda Gates Foundation (Grant number OPP1128907, Contract), http://www.gatesfoundation.org/, and Fundação de Amparo a Pesquisa de Minas Gerais, Brazil, http://www.fapemig.br/en/, non-profit sectors. The clinical trial is funded by the Brazilian Ministry of Health, Program of Development of the Industrial Health Complex (PROCIS), project 23072.052747/2017-51, trial sponsor contact: Fotini Toscas, e-mail fotini.toscas@saude.gov.br. The funders played no role in the study design, data collection and analysis, decision to publish, or preparation of the manuscript.

For peer review only - http://bmjopen.bmj.com/site/about/guidelines.xhtml

Acknowledgements

The authors would like to thank Centre for Clinical Research of Hospital das Clínicas, Universidade Federal de Minas Gerais for general support and Good clinical practices training; Fotini Toscas from the Brazilian Ministry of Health for the active intermediation as the trial sponsor contact.

Roles and responsibilities: ZSNR is the Principal Investigator and coordinator of the Directive Committee. JSG is the coordinator of the Data Management Team and will continuously receive report adverse events of trial interventions or trial conduct. RAPLA is the coordinator of the Clinical Trial Quality Committee, responsible for important protocol modifications, if necessary.

Conflict of interests statement

Authors declare a patent deposit on behalf of the Universidade Federal de Minas Gerais and Fundação de Amparo a Pesquisa de Minas Gerais, Brazil, http://www.fapemig.br/en/. The inventors were Reis, Zilma Silveira Nogueira and Guimaraes, Rodney Nascimento: BR1020170235688 (CTIT-PN862).

Figure 1. Secondary outcome comparisons between the reference GA and the Preemie-Test in a simulated scenario without best pregnancy dating

Legends: *Gestational age from crown-rump-length data adjusted to Intergrowth's 21st fetal standard[19]. R: reference. GA: gestational age. T: test. C1: comparator 1 is the gestational age calculated using the first ultrasound

For peer review only

exam after 13 weeks and 6 days and before 22 weeks of gestation. C2: comparator 2 is the gestational age based on a reliable last menstrual period.

Figure 2. Participant timeline of the study

Legends: GA: gestational age. R: reference. LMP: last menstrual period.

For peer review only

Word Count: 3415 words

For peer review only - http://bmjopen.bmj.com/site/about/guidelines.xhtml

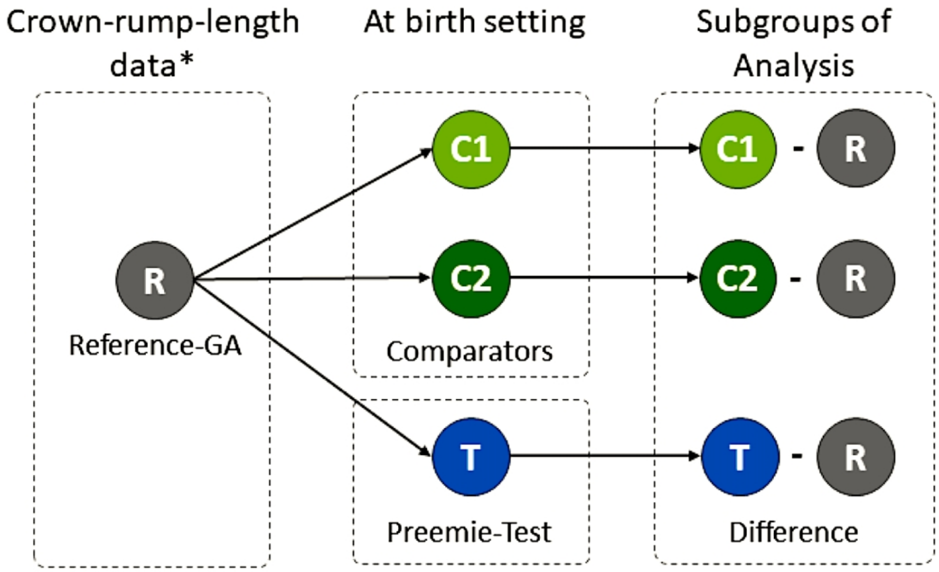

Secondary outcome comparisons between the reference GA and the Preemie-Test in a simulated scenario without best pregnancy dating

Legends: *Gestational age from crown-rump-length data adjusted to Intergrowth's 21st fetal standard19. R: reference. GA: gestational age. T: test. C1: comparator 1 is the gestational age calculated using the first ultrasound exam after 13 weeks and 6 days and before 22 weeks of gestation. C2: comparator 2 is the gestational age based on a reliable last menstrual period.

143x90mm (300 x 300 DPI)

	STUDY PERIOD			
	Enrollment	Assessment	Close-out	Allocation
TIMEPOINT	0	0	72 hours	Analysis
**ENROLLMENT:** Eligibility screen	X			
Informed consent	X			
**INTERVENTION:** Preemie-Test		X		
**ASSESSMENTS AND ANALYSIS:** Preemie-Test: data acquisition		X		
Reference GA: calculated by obstetric ultrasound at <14 weeks of gestation	X			X
Comparator 1: GA calculated by obstetric ultrasound at ≥ 14 and <22 weeks	X			X
Comparator 2: GA calculated by reliable LMP	X			X
Case-control nested study: lung maturity		⟷	⟷	

Participant timeline of the study

Legends: GA: gestational age. R: reference. LMP: last menstrual period.

157x123mm (300 x 300 DPI)

TERMO DE CONSENTIMENTO LIVRE E ESCLARECIDO

Detecção da prematuridade através da interação entre a luz e a pele neonatal: a validação do Preemie-Teste

Sob responsabilidade da pesquisadora Profa Zilma Silveira Nogueira Reis

Cara senhora, você está sendo convidada a participar deste estudo porque acaba de ter um parto no hospital (nome do hospital do centro colaborador) ____.

Apresentação do estudo

O objetivo deste estudo é descobrir novas técnicas para estimar a idade de um bebê ao nascer e identificar aqueles que nasceram antes de nove meses, os prematuros. A idade gestacional desconhecida pode aumentar o risco dos bebês no momento de seu nascimento. As técnicas atuais para se estimar a idade do bebê possuem grande margem de erro.

Acreditamos que a pele possui características que, se bem estudadas, podem refletir a idade das pessoas, e também dos bebês. Por isso, estamos desenvolvendo um novo equipamento médico que se encontra em teste. Ele utiliza a luz para avaliar a composição da pele do bebê e detectar sua idade. Os resultados poderão beneficiar os bebês que nascem sem a informação confiável da idade gestacional.

Instituições envolvidas no estudo

O estudo é desenvolvido pela Faculdade de Medicina da Universidade Federal de Minas Gerais (UFMG), em cooperação com maternidades brasileiras, entre elas a que você se encontra internada. A previsão deste estudo é que 787 crianças recém-nascidas sejam examinadas.

A participação no estudo, riscos e cuidados

Convidamos você e seu bebê para participar deste estudo. Isso incluirá um exame na pele do bebê com a luz, uma breve entrevista com você e a consulta aos registros de saúde sobre a gravidez e os do seu bebê neste hospital. Na entrevista serão tomados todos os cuidados a fim de minimizar os constrangimentos para você. A consulta ao prontuário médico será realizada resguardando o direito de sigilo da informação. Pedimos sua permissão para fotografar a caderneta da gestante ou outro documento equivalente, para conferir a idade gestacional calculada pelos ciclos menstruais e pelos exames de ultrassom. As partes da fotografia que contenham sua identificação serão retiradas da imagem e a manteremos até o final do estudo, quando o arquivo será apagado dos registros da pesquisa.

Pedimos sua permissão para fazer um exame na pele de seu bebê, na região da sola do pé, usando um equipamento em teste. O exame é indolor e externo ao corpo, considerado não-invasivo. A parte que encosta no bebê é pequena e não apresenta pontas que possam ferir a sua pele. Outros equipamentos parecidos, que emitem luz, já são usados nos bebês de forma segura. Por exemplo o oxímetro que faz teste do coraçãozinho. Assim como esse, não se espera que ocorram efeitos imediatos ou futuros na saúde do bebê. Os riscos do teste que faremos incluem a exposição do pé do bebê com perda temporária de calor do corpo e estresse. Cuidados serão tomados a fim de minimizar estes desconfortos. Esclarecemos que o teste dura alguns segundos reduzindo ao mínimo chance de causar marcas ou irritação no local. Caso seu bebê apresente sinais de desconforto durante o exame, o mesmo será interrompido. Você ou familiares poderão permanecer junto ao seu filho durante o exame. Nas crianças que estiverem na Unidade Neonatal, o exame será realizado onde ela já está sendo cuidada, acompanhado pelo profissional de saúde que já está cuidando dela. Caso o seu bebê seja prematuro, todos os devidos cuidados serão tomados antes de cada exame para reduzir a chance de perda de calor, seguindo todas as recomendações de um bebê que fica em incubadora.

Esclarecemos que este estudo não trará benefícios diretos a você ou seu filho, entretanto auxiliará na validação de um novo teste que poderá no futuro identificar o bebê prematuro. Os resultados poderão também gerar informações que ajudem a melhorar os cuidados com outros bebês, quando a idade gestacional é desconhecida. Informamos que os resultados da pesquisa serão publicados em revistas científicas e apresentados em congressos, sem contudo revelar sua identidade ou a do bebê.

For peer review only - http://bmjopen.bmj.com/site/about/guidelines.xhtml

As informações obtidas durante a pesquisa serão confidenciais, guardadas em computadores, protegidos por senha e não serão usadas para outros fins. O roubo das informações que coletaremos no estudo é um risco remoto. Para isso, as melhores práticas em segurança de dados serão empregadas. Também poderão ter acesso aos dados da pesquisa o comitê que coordena o estudo, assim como a agência reguladora ANVISA, sem jamais violar a confidencialidade e privacidade dos dados, para que seja possível monitorar se os procedimentos de qualidade e segurança da pesquisa estão sendo seguidos.

Seus direitos como participante

Informamos que a sua participação deve ser voluntária, ou seja, não é obrigatória e caso não concorde ou resolva desistir a qualquer momento isto não trará nenhum constrangimento para você ou para a forma como você será tratada neste hospital. Também não está previsto nenhum tipo de pagamento por sua participação na pesquisa. Este estudo não implica em gastos para você, pois não terá que se deslocar para outro local, permanecer mais tempo no hospital, uma vez que o exame é feito durante sua internação e de seu bebê na maternidade. Caso seja de seu interesse, os resultados do exame que estarão guardados com o pesquisador e lhe serão entregues assim que você solicitar.

Os pesquisadores garantem que acompanharão gratuitamente seu bebê durante a realização do exame e a qualquer momento que se fizer necessário, em qualquer problema que por ventura esteja associado ao estudo ou efeito do teste com a luz.

Este Termo de Consentimento está elaborado em duas vias iguais. Ambas devem ser assinadas por você, pelo pai da criança e pelo pesquisador. Uma via ficará com o participante e a outra com o pesquisador.

O Comitê de Ética em Pesquisa da UFMG pode ser contatado em caso de haver dúvidas quanto aos aspectos éticos da pesquisa, através do telefone (31) 3409-4592 ou endereço completo apresentado a seguir.

Meu nome	
Documento de identidade	
Data de hoje	

Eu declaro que estou em condições de tomar esta decisão e ciente do que foi exposto acima. Autorizo o uso de minhas informações de saúde e as do meu bebê para este projeto de pesquisa, assim como a realização do novo teste. Participo voluntariamente deste estudo e estou ciente que o exame na pele do meu bebê com a luz não traz prejuízo à sua saúde

Assinatura da puérpera:
Assinatura do pai da criança:
Assinatura do pesquisador:

Telefones de contato:
Maternidade Hospital das Clínicas da UFMG – (31) 34099422
Hospital (nome e telefone do hospital colaborador)
Zilma Reis – (31) 985177473 e-mail: skinage.ufmg@gmail.com
Comitê de Ética em Pesquisa da UFMG – Av. Prof. Antônio Carlos, 6627, Unidade Administrativa II, 2º andar, sala 2005, Campus Pampulha, CEP: 31270-901. E-mail:coep@prpq.ufmg.br. Fone (31) 34094592.
Comitê de Ética em Pesquisa do centro colaborador e endereço completo, com e-mail.

For peer review only - http://bmjopen.bmj.com/site/about/guidelines.xhtml

Reporting checklist for protocol of a clinical trial.

Based on the SPIRIT guidelines.

Instructions to authors

Complete this checklist by entering the page numbers from your manuscript where readers will find each of the items listed below.

Your article may not currently address all the items on the checklist. Please modify your text to include the missing information. If you are certain that an item does not apply, please write "n/a" and provide a short explanation.

Upload your completed checklist as an extra file when you submit to a journal.

In your methods section, say that you used the SPIRIT reporting guidelines, and cite them as:

Chan A-W, Tetzlaff JM, Altman DG, Laupacis A, Gøtzsche PC, Krleža-Jerić K, Hróbjartsson A, Mann H, Dickersin K, Berlin J, Doré C, Parulekar W, Summerskill W, Groves T, Schulz K, Sox H, Rockhold FW, Rennie D, Moher D. SPIRIT 2013 Statement: Defining standard protocol items for clinical trials. Ann Intern Med. 2013;158(3):200-207

		Reporting Item	Page Number
Title	#1	Descriptive title identifying the study design, population, interventions, and, if applicable, trial acronym	1
Trial registration	#2a	Trial identifier and registry name. If not yet registered, name of intended registry	2
Trial registration: data set	#2b	All items from the World Health Organization Trial Registration Data Set	2
Protocol version	#3	Date and version identifier	6
Funding	#4	Sources and types of financial, material, and other support	18
Roles and responsibilities: contributorship	#5a	Names, affiliations, and roles of protocol contributors	19

For peer review only

Roles and responsibilities: sponsor contact information	#5b	Name and contact information for the trial sponsor	19
Roles and responsibilities: sponsor and funder	#5c	Role of study sponsor and funders, if any, in study design; collection, management, analysis, and interpretation of data; writing of the report; and the decision to submit the report for publication, including whether they will have ultimate authority over any of these activities	18
Roles and responsibilities: committees	#5d	Composition, roles, and responsibilities of the coordinating centre, steering committee, endpoint adjudication committee, data management team, and other individuals or groups overseeing the trial, if applicable (see Item 21a for data monitoring committee)	18
Background and rationale	#6a	Description of research question and justification for undertaking the trial, including summary of relevant studies (published and unpublished) examining benefits and harms for each intervention	4
Background and rationale: choice of comparators	#6b	Explanation for choice of comparators	4
Objectives	#7	Specific objectives or hypotheses	5
Trial design	#8	Description of trial design including type of trial (eg, parallel group, crossover, factorial, single group), allocation ratio, and framework (eg, superiority, equivalence, non-inferiority, exploratory)	6
Study setting	#9	Description of study settings (eg, community clinic, academic hospital) and list of countries where data will be collected. Reference to where list of study sites can be obtained	6
Eligibility criteria	#10	Inclusion and exclusion criteria for participants. If applicable, eligibility criteria for study centres and	7

For peer review only

		individuals who will perform the interventions (eg, surgeons, psychotherapists)	
Interventions: description	#11a	Interventions for each group with sufficient detail to allow replication, including how and when they will be administered	7-8
Interventions: modifications	#11b	Criteria for discontinuing or modifying allocated interventions for a given trial participant (eg, drug dose change in response to harms, participant request, or improving / worsening disease)	9
Interventions: adherance	#11c	Strategies to improve adherence to intervention protocols, and any procedures for monitoring adherence (eg, drug tablet return; laboratory tests)	NA
Interventions: concomitant care	#11d	Relevant concomitant care and interventions that are permitted or prohibited during the trial	9
Outcomes	#12	Primary, secondary, and other outcomes, including the specific measurement variable (eg, systolic blood pressure), analysis metric (eg, change from baseline, final value, time to event), method of aggregation (eg, median, proportion), and time point for each outcome. Explanation of the clinical relevance of chosen efficacy and harm outcomes is strongly recommended	9-10
Participant timeline	#13	Time schedule of enrolment, interventions (including any run-ins and washouts), assessments, and visits for participants. A schematic diagram is highly recommended (see Figure)	11
Sample size	#14	Estimated number of participants needed to achieve study objectives and how it was determined, including clinical and statistical assumptions supporting any sample size calculations	11-12
Recruitment	#15	Strategies for achieving adequate participant enrolment to reach target sample size	7

For peer review only

Allocation: sequence generation	#16a	Method of generating the allocation sequence (eg, computer-generated random numbers), and list of any factors for stratification. To reduce predictability of a random sequence, details of any planned restriction (eg, blocking) should be provided in a separate document that is unavailable to those who enrol participants or assign interventions	NA
Allocation concealment mechanism	#16b	Mechanism of implementing the allocation sequence (eg, central telephone; sequentially numbered, opaque, sealed envelopes), describing any steps to conceal the sequence until interventions are assigned	NA
Allocation: implementation	#16c	Who will generate the allocation sequence, who will enrol participants, and who will assign participants to interventions	NA
Blinding (masking)	#17a	Who will be blinded after assignment to interventions (eg, trial participants, care providers, outcome assessors, data analysts), and how	8
Blinding (masking): emergency unblinding	#17b	If blinded, circumstances under which unblinding is permissible, and procedure for revealing a participant's allocated intervention during the trial	NA
Data collection plan	#18a	Plans for assessment and collection of outcome, baseline, and other trial data, including any related processes to promote data quality (eg, duplicate measurements, training of assessors) and a description of study instruments (eg, questionnaires, laboratory tests) along with their reliability and validity, if known. Reference to where data collection forms can be found, if not in the protocol	12
Data collection plan: retention	#18b	Plans to promote participant retention and complete follow-up, including list of any outcome data to be collected for participants who discontinue or deviate from intervention protocols	12
Data management	#19	Plans for data entry, coding, security, and storage, including any related processes to promote data quality	12

For peer review only

		7(eg, double data entry; range checks for data values). Reference to where details of data management procedures can be found, if not in the protocol	
Statistics: outcomes	#20a	Statistical methods for analysing primary and secondary outcomes. Reference to where other details of the statistical analysis plan can be found, if not in the protocol	12
Statistics: additional analyses	#20b	Methods for any additional analyses (eg, subgroup and adjusted analyses)	12
Statistics: analysis population and missing data	#20c	Definition of analysis population relating to protocol non-adherence (eg, as randomised analysis), and any statistical methods to handle missing data (eg, multiple imputation)	NA
Data monitoring: formal committee	#21a	Composition of data monitoring committee (DMC); summary of its role and reporting structure; statement of whether it is independent from the sponsor and competing interests; and reference to where further details about its charter can be found, if not in the protocol. Alternatively, an explanation of why a DMC is not needed	18
Data monitoring: interim analysis	#21b	Description of any interim analyses and stopping guidelines, including who will have access to these interim results and make the final decision to terminate the trial	NA
Harms	#22	Plans for collecting, assessing, reporting, and managing solicited and spontaneously reported adverse events and other unintended effects of trial interventions or trial conduct	19
Auditing	#23	Frequency and procedures for auditing trial conduct, if any, and whether the process will be independent from investigators and the sponsor	12
Research ethics approval	#24	Plans for seeking research ethics committee / institutional review board (REC / IRB) approval	6

For peer review only

Protocol amendments	#25	Plans for communicating important protocol modifications (eg, changes to eligibility criteria, outcomes, analyses) to relevant parties (eg, investigators, REC / IRBs, trial participants, trial registries, journals, regulators)	19
Consent or assent	#26a	Who will obtain informed consent or assent from potential trial participants or authorised surrogates, and how (see Item 32)	6
Consent or assent: ancillary studies	#26b	Additional consent provisions for collection and use of participant data and biological specimens in ancillary studies, if applicable	NA
Confidentiality	#27	How personal information about potential and enrolled participants will be collected, shared, and maintained in order to protect confidentiality before, during, and after the trial	6
Declaration of interests	#28	Financial and other competing interests for principal investigators for the overall trial and each study site	18
Data access	#29	Statement of who will have access to the final trial dataset, and disclosure of contractual agreements that limit such access for investigators	7
Ancillary and post trial care	#30	Provisions, if any, for ancillary and post-trial care, and for compensation to those who suffer harm from trial participation	NA
Dissemination policy: trial results	#31a	Plans for investigators and sponsor to communicate trial results to participants, healthcare professionals, the public, and other relevant groups (eg, via publication, reporting in results databases, or other data sharing arrangements), including any publication restrictions	7
Dissemination policy: authorship	#31b	Authorship eligibility guidelines and any intended use of professional writers	NA
Dissemination policy: reproducible research	#31c	Plans, if any, for granting public access to the full protocol, participant-level dataset, and statistical code	7

For peer review only

Informed consent materials	#32	Model consent form and other related documentation given to participants and authorised surrogates	6
Biological specimens	#33	Plans for collection, laboratory evaluation, and storage of biological specimens for genetic or molecular analysis in the current trial and for future use in ancillary studies, if applicable	NA

The SPIRIT checklist is distributed under the terms of the Creative Commons Attribution License CC-BY-ND 3.0. This checklist can be completed online using https://www.goodreports.org/, a tool made by the EQUATOR Network in collaboration with Penelope.ai

For peer review only

BMJ Open

Prematurity detection evaluating interaction between the skin of the newborn and light: Protocol for the Preemie-Test multicenter clinical trial in Brazilian hospitals to validate a new medical device

Journal:	*BMJ Open*
Manuscript ID	bmjopen-2018-027442.R3
Article Type:	Protocol
Date Submitted by the Author:	08-Jan-2019
Complete List of Authors:	Reis, Zilma; Universidade Federal de Minas Gerais, Gynecology and Obstetrics<br>Guimarães, Rodney; Faculty of Medicine, Universidade Federal de Minas Gerais, Ginecologia e Obstetrícia<br>Rego, Maria Albertina; Faculty of Medicine, Universidade Federal de Minas Gerais, Pediatrics<br>Maia de Castro Romanelli , Roberta ; Universidade Federal de Minas Gerais Faculdade de Medicina,<br>Gaspar, Juliano; Faculty of Medicine, Universidade Federal de Minas Gerais, Obstetrics and Gynecology<br>Vitral, Gabriela; Faculty of Medicine, Universidade Federal de Minas Gerais<br>dos Reis, Marconi; Faculty of Medicine, Universidade Federal de Minas Gerais, Pediatrics<br>Colósimo, Enrico; Universidade Federal de Minas Gerais, Statistics<br>Neves, Gabriela; Hospital Sofia Feldman<br>Vale, Marynea; Hospital Universitario da Universidade Federal do Maranhao, Pediatrics<br>Nader, Paulo; Universidade Luterana do Brasil. Hospital Universitário de Canoas, Pediatrics<br>de Moura, Martha; Hospital Materno Infantil de Brasília<br>de Aguiar, Regina ; Faculty of Medicine, Universidade Federal de Minas Gerais, Obstetrics and Gynecology
**Primary Subject Heading**:	Obstetrics and gynaecology
Secondary Subject Heading:	Global health, Diagnostics, Paediatrics
Keywords:	Gestational Age, Infant, Premature, Skin Physiological Phenomena, Photomedicine, Equipment and Supplies

SCHOLARONE™
Manuscripts

Prematurity detection evaluating interaction between the skin of the newborn and light: Protocol for the Preemie-Test multicenter clinical trial in Brazilian hospitals to validate a new medical device

Zilma Silveira Nogueira Reis[1]*; Rodney Nascimento Guimarães[1]; Maria Albertina Santiago Rego[1]; Roberta Maia de Castro Romanelli[1]; Juliano de Souza Gaspar[1]; Gabriela Luíza Nogueira Vitral[1]; Marconi Augusto Aguiar dos Reis[1]; Enrico Antônio Colósimo[2]; Gabriela Silveira Neves[3]; Marynea Silva do Vale[4]; Paulo de Jesus Nader[5]; Martha David Rocha de Moura[6]; Regina Amélia Pessoa Lopes de Aguiar[1]

1 - Faculty of Medicine, Universidade Federal de Minas Gerais, Brazil.

2 - Statistics Department, Universidade Federal de Minas Gerais, Brazil.

3 - Hospital Sofia Feldman, Brazil.

4 - Hospital Universitário da Universidade Federal do Maranhão, Brazil.

5 - Universidade Luterana do Brasil. Hospital Universitário de Canoas, Brazil.

6 - Hospital Materno Infantil de Brasília, Brazil.

*Corresponding author: Zilma Silveira Nogueira Reis. Avenida Professor Alfredo Balena, 190, sala 601. Zip Code 30.130.100 Belo Horizonte, Brazil. Phone: +55 0 31 3409-9467. Email: zilma.medicina@gmail.com

For peer review only

Abstract

For peer review only - http://bmjopen.bmj.com/site/about/guidelines.xhtml

Introduction: Recognizing prematurity is critical in order to attend to immediate needs in childbirth settings, guiding the extent of medical care provided for newborns. A new medical device has been developed to carry out the Preemie-Test, an innovative approach to estimate gestational age (GA), based on the photobiological properties of the newborn's skin. This study will validate the Preemie-Test for GA estimation at birth and its accuracy to detect prematurity. Secondarily, the study intends to associate the infant´s skin reflectance with lung maturity, as well as evaluate safety, precision, and usability of a new medical device to offer a suitable product for health professionals during childbirth and in neonatal care settings.

Methods and analysis: Research protocol for diagnosis, single-group, single-blinding, and single-arm multicenter clinical trials with a reference standard. Alive newborns, with 24 weeks or more of pregnancy age, will be enrolled during the first 24 hours of life. Sample size is 787 subjects. The primary outcome is the difference between the GA calculated by the photobiological neonatal skin assessment methodology and the GA calculated by the comparator antenatal ultrasound or reliable last menstrual period. Immediate complications caused by pulmonary immaturity during the first 72 hours of life will be associated with skin reflectance in a nested case-control study.

Ethics and dissemination: Each local independent ethics review board approved the trial protocol. The authors intend to share the minimal anonymized data set necessary to replicate study findings.

Trial registration number: Brazilian Clinical Trials Registry (ReBec) RBR-3f5bm5.

Key-words: Gestational Age, Infant, Premature; Skin Physiological Phenomena; Photomedicine; Equipment and Supplies.

For peer review only - http://bmjopen.bmj.com/site/about/guidelines.xhtml

Article Summary

Strengths and limitations of this study:

- Prospective multicenter evaluation of a new medical device with training, and certification of collaborative centers.
- The gold standard comparator for pregnancy dating does not exist; instead a reference standard will be used with blinded primary outcome.
- The agreement endpoint between methods for gestational age determination precludes randomization of the intervention.

For peer review only

For peer review only - http://bmjopen.bmj.com/site/about/guidelines.xhtml

Introduction

In childbirth settings, health professionals continuously need to make timely decisions to provide proper neonatal care. The day of birth is the riskiest for newborns and mothers almost everywhere[1]. Perinatal causes related to prematurity and complications during childbirth, which are generally preventable through qualified health care, are the primary causes of death among newborns[1,2]. Most of these deaths took place in countries with low resources and a scarcity of health facilities[3]. The opportune recognition of prematurity is critical in order to judge the viability of the newborn and to attend to his/her immediate needs, guiding the complexity of the medical care provided for the newborn. Without reliable information on the age of the unborn phase, actions to preserve the potential for survival of the newborn can be neglected[4]. Indeed, the attempted management of the risk of mortality and severe complications are sensitive issues to the gestational age (GA), which involves temperature maintenance, ventilatory support, transport to a neonatal intensive care unit (NICU), and the early treatment of respiratory distress syndrome (RDS), the most severe complication of premature birth[5]. In addition to the GA information or birthweight, the prediction of neonatal respiratory morbidity may be critical in planning immediate medical care[6], since the respiratory system is among the last of the fetal organ systems to mature, which is associated with enhanced morbidity and mortality[6].

Current methods of dating pregnancy remain a worldwide challenge. Early obstetric ultrasound currently offers the best due date[7]. However, access to this type of exam is limited because of high equipment costs, poor training and skills of health professionals, or late prenatal care[8]. Despite a 10-days or more margin of error during the second and third trimester of gestation, ultrasound is still a reasonable methodology for GA determination, when the best opportunity was lost[7]. The calculation, based on the historical information of the last menstrual period (LMP), is

For peer review only - http://bmjopen.bmj.com/site/about/guidelines.xhtml

impacted by the uncertainty of both the fertility days and date of conception[9], due to the bias of memory, the use of hormonal contraception, and breastfeeding[10]. After birth, neurological scores, such as the New Ballard[11], show a tendency to overestimate GA in preterm infants and underestimate GA in growth-restricted infants[12]. Efforts to enhance the reliability of pregnancy dating, through more accurate and accessible technologies, seek to improve pregnancy outcomes and neonatal survival[13].

A new medical device has been developed to carry out the Preemie-Test, an innovative approach used to estimate GA, based on the photobiological properties of the newborn's skin. This reflective test is noninvasive, and the device automatically processes the light, scattered by the constituents of the skin layers, when a small optoelectronic light emitter/receiver sensor touches the newborn's skin[14]. The device under test is easy to use and every effort is being made to ensure that it has excellent accuracy, be it safe and low cost. The feasibility study provided a mathematical model to predict GA based on the skin reflectance adjusted to clinical variables (R^2 = 0.828, P <0.001)[15]. However, before the adoption or use of an innovation, an effectiveness trial of intervention is a critical step in the research chain regarding its the social utility when completing the translation from the proof of concept to clinical science[16]. The rationale for the main hypothesis in this study is that the skin maturity of a newborn, obtained by the analysis of its optical properties, is useful in pregnancy dating for clinical use and respiratory prognosis, especially in a scenario with no reliable GA based on current methods. This study aims to validate the photobiological model of the skin, called the "Preemie-test", in order to estimate GA at birth and determine its accuracy in detecting prematurity. Secondarily, it also seeks to associate the infant's skin reflectance with lung maturity. Moreover, this study intends to evaluate the safety, precision, and the usability of a new medical device to offer a suitable product to support health professionals during childbirth and in neonatal care settings.

For peer review only - http://bmjopen.bmj.com/site/about/guidelines.xhtml

Methods

Study design

This study will use a protocol for diagnosis, single-group, single-blinding, and single-arm multicenter clinical trials with a reference standard. This new photobiological approach to the skin, gathered in a medical device, is currently in the pivotal phase of innovation development from the prototype to regulatory approval[17]. This step aims to provide the translation[16] of the scientific model for GA detection based on skin maturity. This Protocol version is 1, July/10th/2018. Faculty of Medicine, Universidade Federal de Minas Gerais is the Coordinator Center.

Study Settings, Ethics and Dissemination

Selected Brazilian referral centers for high-risk pregnancy and neonatal care will participate in the study, according to this protocol: Hospital das Clínicas, Universidade Federal de Minas Gerais, as the Center for Coordination; Hospital Sofia Feldman, Minas Gerais State; Hospital da Universidade Luterana do Brasil, Rio Grande do Sul State; Hospital Materno-infantil de Brasília, Distrito Federal; and Hospital Universitário da Universidade Federal do Maranhão, Maranhão State. Each local independent ethics review board approved the trial protocol, and the Brazilian National Research Council (CONEP) approved all study activities and protocol prior to the commencement of study activities, in accordance with the Declaration of Helsinki (2008), good clinical practice as set forth by the International Organization for Standardization (ISO) 14155:2011, and the Brazilian regulatory health agency's recommendations[18]. This study was logged under both protocol number CAAE 81347817.6.1001.5149 and the International Clinical Trials Registry Platform under number RBR-3f5bm5. Parents will sign an informed consent form on behalf of the newborn before participating in the clinical trial (supplementary file).

For peer review only - http://bmjopen.bmj.com/site/about/guidelines.xhtml

Data Sharing Statement

The authors intend to share the minimal anonymized data set necessary to replicate study findings. Data sharing will include: the reference and comparators GA, GA estimated by the Preemie-test, birth weight, RDS or transient tachypnea of the newborn (TTN) diagnosis, ventilatory support due to pulmonary immaturity, neonatal intensive care unit (NICU) admission due to RDS or TTN, and any adverse events regarding device's safety. Unidentified data and study-related documents as ethical approvals will be accessible by URLs for researchers, regulatory agencies, and sponsors.

Patient and Public Involvement

Patients and the public were not involved in the design of this study. The results will be disseminated to study parents of participants through scientific publications, non-scientific publications, and on the website of the project: http://skinage.medicina.ufmg.br.

Eligibility criteria and participant's timeline

A prospective sequential and concurrent enrollment process will select newborns in referral hospitals centers for neonatal care. Infants are eligible with the following inclusion criteria: (1) alive newborn; (2) enrollment during first 24 hours of life; (3) be 24 weeks or more of gestational age, at birth; (4) fetus underwent an obstetric ultrasound assessment before 14 weeks of pregnancy; (5) fetus also had obstetric ultrasound assessment between 14 and 22 gestational weeks. Exclusion criteria are: (1) malformation with structural skin alterations; (2) skin modifiers: anhydramnios, hydrops, congenital skin diseases or chorioamnionitis. Randomisation was not appropriate to assess the agreement between different methods to assess pregnancy dating.

In a nested case-control study, we will select newborns within the first 72 hours of life,

For peer review only - http://bmjopen.bmj.com/site/about/guidelines.xhtml

discharge, or death, whichever occurs first, with the following inclusion criteria: (1) RDS or (2) TTN diagnosis. Ranges of gestational age will randomly pair controls. Exclusion criteria include: (1) the existence of extra pulmonary conditions with tachypnea not due to prematurity and (2) diagnosis of Clinical or Laboratory-Confirmed Bloodstream Infection.

Intervention: The Preemie-Test

The Preemie-Test assessment occurs as soon as possible after birth, in the first 24 hours, inside incubators, open heating crib, common crib or in the mother's lap, in order to ensure minimum manipulation and stable clinical conditions. The acquisitions of all newborns will be stored in a database for further statistical analysis.

A noninvasive, handheld optoelectronic prototype has been developed to measure the backscattered light signal from the skin[15]. The equipment regulates the emitted light and processes the received light signal in the sensor, resulting in the prediction of GA by a mathematical model, associated or not with clinical variables. According to the Brazilian regulatory health agency (ANVISA), this medical device is categorized as a Class II safety: noninvasive and medium risk. The prototype unit of measurement and the process of GA estimation were patented under number BR1020170235688 (CTIT-PN862)[14]. An updated version of the invention received improvements in order to safeguard reliability and to minimize examiner interferences on the skin's backscattering acquisition. The light emitting-sensor touches the skin over the sole of the foot for a few seconds. The skin reflectance will be sensed once the light has been emitted by a light emitting diode (LED) at wavelengths from 400 nm to 1200 nm. Data acquisitions occur automatically, without operator influence, and are obtained three times per newborn, in the same site and sequentially. Digital recordings will be uploaded to a server for further analysis. The prototype will blind the examiner to the predicted GA value.

For peer review only - http://bmjopen.bmj.com/site/about/guidelines.xhtml

The criterium for discontinuing the interventions for a given trial participant will be in case of parents of the newborns' request.

Training and monitoring

Systematic monitoring of data collection, through an electronic information system, would trigger any adverse event. This medical team is still responsible for the training of healthcare professionals to recruit participants, data collection, a safely performed Preemie-Test during the newborn's assessment, and the monitoring of data quality. The certification of co-participant centers involved the accomplishment of at least 30 simulated examinations by the participant health professionals in the study.

Gestational age methods of calculation and comparators

Reference-GA (R) is calculated upon enrollment, using the embryo measurement assessed by ultrasound exam at <14 weeks of gestation as a reference. Crown-rump-length (CRL) data, recorded from the ultrasound report or prenatal care book-document, will be considered the crude data, when available. Intergrowth's 21st standard curve for ultrasound measurements from 7 weeks and 3 days up to 13 weeks and 6 days will be adjusted to all GA data, according to CRL[19].

GA methods to calculate GA in the childbirth setting, and their comparators are as follows:

- Preemie-Test-GA (T): data statistically determined by analyzing the acquired information stored in the device's processor.
- Comparators-GA (C): calculated using the first ultrasound exam after 13 weeks and 6 days of gestation and before 22 weeks (C1). When available, a second comparator is GA based on a reliable LMP (C2)[13].

For peer review only - http://bmjopen.bmj.com/site/about/guidelines.xhtml

We will take a scanning copy of the prenatal care book or the ultrasound report. After evaluating the data quality, the images will be discarded. To achieve a reliable LMP, we will interview the woman, as suggested by Nguyen et al. (2000)[13].

Primary outcome measures

The primary target is the agreement between the GA offered by the Preemie-Test (T) and the GA calculated by the comparators (C1 and C2), so as to perform the new test in scenarios without the Reference-GA (R). The outcome is the difference between the GA calculated by the photobiological neonatal skin assessment methodology in relation to the age calculated by the comparators.

Another measure for the primary target is the detection of preterm newborns, considering the age before 37 weeks of pregnancy as the threshold between term and preterm births, and analyzing sub-categories of preterm birth, based on GA[4]:

- extremely preterm (less than 28 weeks)
- very preterm (28 to 32 weeks)
- moderate to late preterm (more than 32 to less than 37 weeks).

In this case, the outcome is the proportion of the preterm newborn correctly detected at birth, based on the photobiological test of the skin, within a one-week error.

Secondary outcome measures

1. In a simulated scenario, in which the Reference-GA (R) is unknown, two groups will be randomly assigned from the complete database in order to compare differences among the Reference-GA (R), the GA obtained through the Preemie-Test (T), and the GA calculated by the comparators. Figure 1 presents such subgroups and measures for comparison.

For peer review only - http://bmjopen.bmj.com/site/about/guidelines.xhtml

2. To monitor the device's safety when in regular use by participants over a 72-hour period. Adverse events will be monitored, according to ISO 14155:2011 standards. This means any unexpected medical events, unintended disease or injury, or unfortunate clinical signs in subjects, users, or other people, whether related to the investigational medical device or not.
3. To establish the *ease of use* of the Preemie-Test measurement as a potential method for preterm newborn diagnosis.

The secondary outcome measures in the case-control nested study

Immediate complications, occurring during the first 72 hours of life due to pulmonary immaturity, are the secondary target. The outcome measures are as follows:

- To describe the relationship of the measurement of the newborn's skin reflectance with RDS and with diagnoses based on clinical and radiological findings and respiratory outcomes[6,20].
- To describe the relationship of the measurement of the newborn's skin reflectance with the TTN and with diagnoses based on clinical findings and respiratory outcomes[6].
- To describe the relationship of the measurement of the newborn's skin reflectance with ventilatory support due to pulmonary immaturity.
- To describe the relationship of the measurement of the newborn's skin reflectance with NICU admission due to RDS or TTN.

Time schedule of enrollment, intervention, and outcome measurements are presented in a schematic diagram (see Figure 2). The assessment occurs during the first 24 hours of life, but participants will be followed up for 72 hours or until discharge or death, whichever occurs first, for the monitoring of neonatal outcomes and adverse events.

Sampling and sample size

For peer review only - http://bmjopen.bmj.com/site/about/guidelines.xhtml

The sample size calculation is estimated based on the primary endpoint. To test the hypothesis of equivalence between the Preemie-Test GA and the comparators GA, a sample of 787 subjects is necessary to detect an effect size of 10%. Using the G-Power 3.1 software[21], we assumed an alpha error of 0.05, and a power of test of 0.80 to support a paired t-test.

Sampling intends to arrange three groups of GA enrollment to preserve enough premature newborns with 3:2:1 proportion, similar to Wilson et al. (2017)[22]: 392 term newborns, 263 premature newborns from 32 to 36 weeks and six days of GA, and 132 extremely premature newborns from 24 to 31 weeks and six days of GA.

Usability

The usability assessment will be performed by applying a checklist to participants who use the prototype device to perform the Preemie-Test. The 10 heuristics proposed by Nielsen and Marck (1994)[23] will be adapted to build a checklist to evaluate the device, namely: (a) system visibility, (b) correspondence with the real world, (c) user control and freedom, (d) consistency of results and standardization, (e) error prevention, (f) visual recognition rather than memorization, (g) flexibility and efficiency of use, (h) esthetic and minimalist design, (i) help for the user to recognize, diagnose, and recover from errors, and (j) user documentation and help.

Data collection

Standard operational procedures set data entries in structured questionaries. In this concurrent clinical trial, an electronic information system was developed to collect data in different hospitals, simultaneously. Entry forms validations were implemented with data values ranges to ensure the quality of the information. An audit of the data will be permanently performed and the data summary available on the project webpage. Double system, paper-based and electronic will permit audit concerning reliability and

For peer review only - http://bmjopen.bmj.com/site/about/guidelines.xhtml

validity. Independent rater over-read all papers files and cross check with the electronic information from all patients.

Data analysis

Demographics and baseline characteristics of the study group, as well the intervention measurements, will be summarized by the frequencies and the mean and standard deviation (SD), the whereas median and interquartile range will be preferred for non-normally distributed continuous variables.

To model the GA prediction, computational randomization will select two subsamples in the database. One of them to train the prediction model of GA based on skin reflectance and clinical variables, such as sex, time in an incubator, phototherapy, birth weight, among others. Another part will be for the analytical validation of the predictive model. Improvements in the existing prediction models for GA (Preemie-Test), will be conducted with conventional statistical and data mining analyses.

Regarding the primary endpoint, the agreement among three methods for GA will be calculated using the Intraclass coefficient correlation and Bland & Altman plots[24], and paired t-testing. The accuracy of the Preemie-Test in identifying the premature newborn, within a one-week margin of error, will be the target of the accuracy analysis.

The relationship between the measurement of the newborn's skin reflectance and complications due to pulmonary distress associated with immaturity will be evaluated by means of association tests and risk. The significance level for hypothesis tests will be 5%, together with 95% confidence intervals.

Discussion

Strengths and Limitations

For peer review only - http://bmjopen.bmj.com/site/about/guidelines.xhtml

Availability of trustworthy GA information is a prerequisite for preterm birth classification and healthcare decisions[25]. In this light, the results of this clinical study have the potential to validate a new device for pregnancy dating. The Preemie-Test was prepared to operate with minimum operator intervention and for use by healthcare professionals anywhere a birth takes place without a reliable GA.

The purpose of medical research involving neonates is intended to improve clinical procedures[26]. In this context, a clinical trial is a research study in which subjects are prospectively assigned to intervention and the effects of those interventions on health-related outcomes are thereby evaluated[27]. However, clinical trials on medical devices face barriers when an effective standard procedure does not exist, as is the case of the comparator procedure[28]. Our challenge in preparing the present protocol was the absence of a gold standard for pregnancy dating, since the fetal age begins upon conception; however, this information is difficult to be accurately determined[7].

The study begun with the training of health professionals in September 2018. It is anticipated that the recruitment will take place from January to December 2019. Data analysis will be finalized, the results of which are expected in May 2020.

References

1. Children St. Surviving the first day. In: *State of the world's mothers 2013.* Vol 1. Connecticut: Save the Children; 2013: http://www.savethechildren.org/atf/cf/%7B9def2ebe-10ae-432c-9bd0-df91d2eba74a%7D/SOWM-FULL-REPORT_2013.PDF.

2. França EB, Lansky S, Rego MAS, et al. Principais causas da mortalidade na infância no Brasil, em 1990 e 2015: estimativas do estudo de Carga Global de Doença. *Revista Brasileira de Epidemiologia.* 2017;20:46-60.

For peer review only - http://bmjopen.bmj.com/site/about/guidelines.xhtml

For peer review only

3. Federation StC. HNN website, Healthy Newborn Network. https://www.healthynewbornnetwork.org/. Published 2018. Accessed 01/17/2018, 2018.

4. Howson CP, Kinney MV, McDougall L, Lawn JE, Group BTSPBA. Born too soon: preterm birth matters. *Reprod Health.* 2013;10 Suppl 1:S1.

5. Bhutta ZA, Giuliani F, Haroon A, et al. Standardisation of neonatal clinical practice. *BJOG.* 2013;120 Suppl 2:56-63, v.

6. Liszewski MC, Stanescu AL, Phillips GS, Lee EY. Respiratory Distress in Neonates: Underlying Causes and Current Imaging Assessment. *Radiol Clin North Am.* 2017;55(4):629-644.

7. Committee on Obstetric Practice tAIoUiM, and the Society for Maternal-Fetal Medicine. Committee Opinion No 700: Methods for Estimating the Due Date. *Obstet Gynecol.* 2017;129(5):e150-e154.

8. Karl S, Li Wai Suen CS, Unger HW, et al. Preterm or not--an evaluation of estimates of gestational age in a cohort of women from Rural Papua New Guinea. *PLoS One.* 2015;10(5):e0124286.

9. Wilcox AJ, Weinberg CR, Baird DD. Timing of Sexual Intercourse in Relation to Ovulation — Effects on the Probability of Conception, Survival of the Pregnancy, and Sex of the Baby. *New England Journal of Medicine.* 1995;333(23):1517-1521.

10. Lynch CD, Zhang J. The research implications of the selection of a gestational age estimation method. *Paediatr Perinat Epidemiol.* 2007;21 Suppl 2:86-96.

For peer review only - http://bmjopen.bmj.com/site/about/guidelines.xhtml

11. Ballard JL, Khoury JC, Wedig K, Wang L, Eilers-Walsman BL, Lipp R. New Ballard Score, expanded to include extremely premature infants. *The Journal of Pediatrics.* 1991;119(3):417-423.

12. Lee AC, Panchal P, Folger L, et al. Diagnostic Accuracy of Neonatal Assessment for Gestational Age Determination: A Systematic Review. *Pediatrics.* 2017;140(6).

13. Nguyen TH, Larsen T, Engholm G, Møller H. Increased adverse pregnancy outcomes with unreliable last menstruation. *Obstet Gynecol.* 2000;95(6 Pt 1):867-873.

14. Reis ZSN, Guimarães RN, Inventors. Dispositivo para determinação da idade gestacional, processos e usos. Nov/1/2016, 2016.

15. Reis ZSN, Simeoni U, Vitral GLN, Souza IMFd, Rego MAS, Guimaraes RN. Newborn skin reflection: Proof of concept for a new approach for predicting gestational age at birth. A cross-sectional study. *Plos One.* 2017.

16. Reis ZSN, Vitral GLN, de Souza IMF, Rego MAS, Guimaraes RN. Newborn skin reflection: Proof of concept for a new approach for predicting gestational age at birth. A cross-sectional study. *PLOS ONE.* 2017;12(9):e0184734.

17. Kaplan AV, Baim DS, Smith JJ, et al. Medical device development: from prototype to regulatory approval. *Circulation.* 2004;109(25):3068-3072.

18. Brasil. Resolução da Diretoria Colegiada - RDC No. 10. In: Sanitária AnNdVn, ed. Vol 10. Brasilia: Diário Oficial da União, de 03 de março de 2015; 2015.

19. Papageorghiou AT, Kennedy SH, Salomon LJ, et al. International standards for early fetal size and pregnancy dating based on ultrasound measurement of crown-

For peer review only

For peer review only - http://bmjopen.bmj.com/site/about/guidelines.xhtml

rump length in the first trimester of pregnancy. *Ultrasound Obstet Gynecol.* 2014;44(6):641-648.

20. Reuter S, Moser C, Baack M. Respiratory distress in the newborn. *Pediatr Rev.* 2014;35(10):417-428; quiz 429.

21. Faul F, Erdfelder E, Lang A-G, Buchner A. G* Power 3: A flexible statistical power analysis program for the social, behavioral, and biomedical sciences. *Behavior research methods.* 2007;39(2):175-191.

22. Wilson K, Hawken S, Murphy MS, et al. Postnatal Prediction of Gestational Age Using Newborn Fetal Hemoglobin Levels. *EBioMedicine.* 2017;15:203-209.

23. Nielsen J. Usability inspection methods. Conference Companion on Human Factors in Computing Systems; 1994; Boston, Massachusetts, USA.

24. Altman DG BJ. Measurement in medicine: the analysis of method comparison studies. 1983:307-317

25. Beydoun H, Ugwu B, Oehninger S. Assisted reproduction for the validation of gestational age assessment methods. *Reproductive biomedicine online.* 2011;22(4):321-326.

26. WHO. Clinical Trials in Children. Essential medicines for children. World Health Organisation. http://www.who.int/ictrp/child/en/. Accessed 08/01/2018, 2018.

27. USA. NIH's Definition of clinical trial. In: Services USDoHaH, ed. Bethesda: National Institutes of Health; 2018.

28. Neugebauer EAM, Rath A, Antoine SL, et al. Specific barriers to the conduct of randomised clinical trials on medical devices. *Trials.* 2017;18(1):427.

For peer review only - http://bmjopen.bmj.com/site/about/guidelines.xhtml

Author statement:

ZSNR: designed the study, planned data collection, prepared the team for good clinical practices, wrote and revised the paper. RNG, RAPLA, MASR, RMCR and JSG made substantial contributions to study design, planned data collection, prepared the team for good clinical practices, wrote and revised the paper. GLNV, MAAR, GSN, PJN, MDRM, and MSV made contributions to standard procedures in methods, drafted the manuscript, reviewed the paper, and approved the final manuscript. EAC: drafted the work and reviewed it critically for important intellectual content, as statistic consultant.

Funding statement

This research was supported by the Grand Challenges Exploration from the Bill & Melinda Gates Foundation (Grant number OPP1128907, Contract), http://www.gatesfoundation.org/, and Fundação de Amparo a Pesquisa de Minas Gerais, Brazil, http://www.fapemig.br/en/, non-profit sectors. The clinical trial is funded by the Brazilian Ministry of Health, Program of Development of the Industrial Health Complex (PROCIS), project 23072.052747/2017-51, trial sponsor contact: Fotini Toscas, e-mail fotini.toscas@saude.gov.br. The funders played no role in the study design, data collection and analysis, decision to publish, or preparation of the manuscript.

Acknowledgements

The authors would like to thank Center for Clinical Research of Hospital das Clínicas, Universidade Federal de Minas Gerais for general support and Good clinical practices

For peer review only - http://bmjopen.bmj.com/site/about/guidelines.xhtml

training; Fotini Toscas from the Brazilian Ministry of Health for the active intermediation as the trial sponsor contact.

Roles and responsibilities: ZSNR is the Principal Investigator and coordinator of the Directive Committee. JSG is the coordinator of the Data Management Team and will continuously receive report adverse events of trial interventions or trial conduct. RAPLA is the coordinator of the Clinical Trial Quality Committee, responsible for important protocol modifications, if necessary.

Conflict of interests statement

Authors declare a patent deposit on behalf of the Universidade Federal de Minas Gerais and Fundação de Amparo a Pesquisa de Minas Gerais, Brazil, http://www.fapemig.br/en/. The inventors were Reis, Zilma Silveira Nogueira and Guimaraes, Rodney Nascimento: BR1020170235688 (CTIT-PN862).

Figure 1. Secondary outcome comparisons between the reference GA and the Preemie-Test in a simulated scenario without best pregnancy dating

Legends: *Gestational age from crown-rump-length data adjusted to Intergrowth's 21st fetal standard[19]. R: reference. GA: gestational age. T: test. C1: comparator 1 is the gestational age calculated using the first ultrasound exam after 13 weeks and 6 days and before 22 weeks of gestation. C2: comparator 2 is the gestational age based on a reliable last menstrual period.

For peer review only - http://bmjopen.bmj.com/site/about/guidelines.xhtml

Figure 2. Participant timeline of the study

Legends: GA: gestational age. R: reference. LMP: last menstrual period.

Word Count: 3415 words

For peer review only

For peer review only - http://bmjopen.bmj.com/site/about/guidelines.xhtml

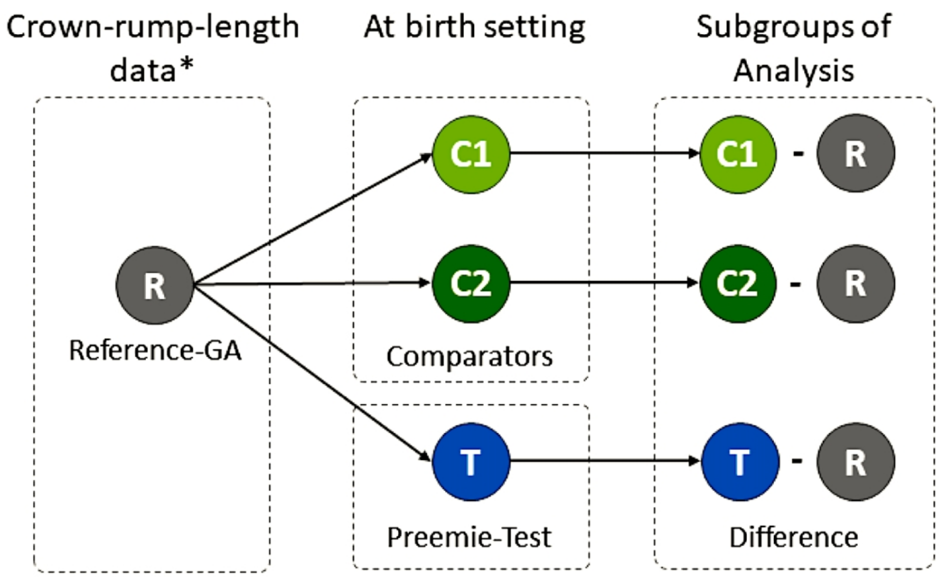

Secondary outcome comparisons between the reference GA and the Preemie-Test in a simulated scenario without best pregnancy dating

Legends: *Gestational age from crown-rump-length data adjusted to Intergrowth's 21st fetal standard19. R: reference. GA: gestational age. T: test. C1: comparator 1 is the gestational age calculated using the first ultrasound exam after 13 weeks and 6 days and before 22 weeks of gestation. C2: comparator 2 is the gestational age based on a reliable last menstrual period.

143x90mm (300 x 300 DPI)

	STUDY PERIOD			
	Enrollment	Assessment	Close-out	Allocation
TIMEPOINT	0	0	72 hours	Analysis
**ENROLLMENT:**				
Eligibility screen	X			
Informed consent	X			
**INTERVENTION:**				
Preemie-Test		X		
**ASSESSMENTS AND ANALYSIS:**				
Preemie-Test: data acquisition		X		
Reference GA: calculated by obstetric ultrasound at <14 weeks of gestation	X			X
Comparator 1: GA calculated by obstetric ultrasound at ≥ 14 and <22 weeks	X			X
Comparator 2: GA calculated by reliable LMP	X			X
Case-control nested study: lung maturity		◆——	——◆	

Participant timeline of the study

Legends: GA: gestational age. R: reference. LMP: last menstrual period.

157x123mm (300 x 300 DPI)

TERMO DE CONSENTIMENTO LIVRE E ESCLARECIDO

Detecção da prematuridade através da interação entre a luz e a pele neonatal: a validação do Preemie-Teste

Sob responsabilidade da pesquisadora Profa Zilma Silveira Nogueira Reis

Cara senhora, você está sendo convidada a participar deste estudo porque acaba de ter um parto no hospital (nome do hospital do centro colaborador) ____.

Apresentação do estudo

O objetivo deste estudo é descobrir novas técnicas para estimar a idade de um bebê ao nascer e identificar aqueles que nasceram antes de nove meses, os prematuros. A idade gestacional desconhecida pode aumentar o risco dos bebês no momento de seu nascimento. As técnicas atuais para se estimar a idade do bebê possuem grande margem de erro.

Acreditamos que a pele possui características que, se bem estudadas, podem refletir a idade das pessoas, e também dos bebês. Por isso, estamos desenvolvendo um novo equipamento médico que se encontra em teste. Ele utiliza a luz para avaliar a composição da pele do bebê e detectar sua idade. Os resultados poderão beneficiar os bebês que nascem sem a informação confiável da idade gestacional.

Instituições envolvidas no estudo

O estudo é desenvolvido pela Faculdade de Medicina da Universidade Federal de Minas Gerais (UFMG), em cooperação com maternidades brasileiras, entre elas a que você se encontra internada. A previsão deste estudo é que 787 crianças recém-nascidas sejam examinadas.

A participação no estudo, riscos e cuidados

Convidamos você e seu bebê para participar deste estudo. Isso incluirá um exame na pele do bebê com a luz, uma breve entrevista com você e a consulta aos registros de saúde sobre a gravidez e os do seu bebê neste hospital. Na entrevista serão tomados todos os cuidados a fim de minimizar os constrangimentos para você. A consulta ao prontuário médico será realizada resguardando o direito de sigilo da informação. Pedimos sua permissão para fotografar a caderneta da gestante ou outro documento equivalente, para conferir a idade gestacional calculada pelos ciclos menstruais e pelos exames de ultrassom. As partes da fotografia que contenham sua identificação serão retiradas da imagem e a manteremos até o final do estudo, quando o arquivo será apagado dos registros da pesquisa.

Pedimos sua permissão para fazer um exame na pele de seu bebê, na região da sola do pé, usando um equipamento em teste. O exame é indolor e externo ao corpo, considerado não-invasivo. A parte que encosta no bebê é pequena e não apresenta pontas que possam ferir a sua pele. Outros equipamentos parecidos, que emitem luz, já são usados nos bebês de forma segura. Por exemplo o oxímetro que faz teste do coraçãozinho. Assim como esse, não se espera que ocorram efeitos imediatos ou futuros na saúde do bebê. Os riscos do teste que faremos incluem a exposição do pé do bebê com perda temporária de calor do corpo e estresse. Cuidados serão tomados a fim de minimizar estes desconfortos. Esclarecemos que o teste dura alguns segundos reduzindo ao mínimo chance de causar marcas ou irritação no local. Caso seu bebê apresente sinais de desconforto durante o exame, o mesmo será interrompido. Você ou familiares poderão permanecer junto ao seu filho durante o exame. Nas crianças que estiverem na Unidade Neonatal, o exame será realizado onde ela já está sendo cuidada, acompanhado pelo profissional de saúde que já está cuidando dela. Caso o seu bebê seja prematuro, todos os devidos cuidados serão tomados antes de cada exame para reduzir a chance de perda de calor, seguindo todas as recomendações de um bebê que fica em incubadora.

Esclarecemos que este estudo não trará benefícios diretos a você ou seu filho, entretanto auxiliará na validação de um novo teste que poderá no futuro identificar o bebê prematuro. Os resultados poderão também gerar informações que ajudem a melhorar os cuidados com outros bebês, quando a idade gestacional é desconhecida. Informamos que os resultados da pesquisa serão publicados em revistas científicas e apresentados em congressos, sem contudo revelar sua identidade ou a do bebê.

For peer review only - http://bmjopen.bmj.com/site/about/guidelines.xhtml

As informações obtidas durante a pesquisa serão confidenciais, guardadas em computadores, protegidos por senha e não serão usadas para outros fins. O roubo das informações que coletaremos no estudo é um risco remoto. Para isso, as melhores práticas em segurança de dados serão empregadas. Também poderão ter acesso aos dados da pesquisa o comitê que coordena o estudo, assim como a agência reguladora ANVISA, sem jamais violar a confidencialidade e privacidade dos dados, para que seja possível monitorar se os procedimentos de qualidade e segurança da pesquisa estão sendo seguidos.

Seus direitos como participante

Informamos que a sua participação deve ser voluntária, ou seja, não é obrigatória e caso não concorde ou resolva desistir a qualquer momento isto não trará nenhum constrangimento para você ou para a forma como você será tratada neste hospital. Também não está previsto nenhum tipo de pagamento por sua participação na pesquisa. Este estudo não implica em gastos para você, pois não terá que se deslocar para outro local, permanecer mais tempo no hospital, uma vez que o exame é feito durante sua internação e de seu bebê na maternidade. Caso seja de seu interesse, os resultados do exame que estarão guardados com o pesquisador e lhe serão entregues assim que você solicitar.

Os pesquisadores garantem que acompanharão gratuitamente seu bebê durante a realização do exame e a qualquer momento que se fizer necessário, em qualquer problema que por ventura esteja associado ao estudo ou efeito do teste com a luz.

Este Termo de Consentimento está elaborado em duas vias iguais. Ambas devem ser assinadas por você, pelo pai da criança e pelo pesquisador. Uma via ficará com o participante e a outra com o pesquisador.

O Comitê de Ética em Pesquisa da UFMG pode ser contatado em caso de haver dúvidas quanto aos aspectos éticos da pesquisa, através do telefone (31) 3409-4592 ou endereço completo apresentado a seguir.

Meu nome	
Documento de identidade	
Data de hoje	

Eu declaro que estou em condições de tomar esta decisão e ciente do que foi exposto acima. Autorizo o uso de minhas informações de saúde e as do meu bebê para este projeto de pesquisa, assim como a realização do novo teste. Participo voluntariamente deste estudo e estou ciente que o exame na pele do meu bebê com a luz não traz prejuízo à sua saúde

Assinatura da puérpera:
Assinatura do pai da criança:
Assinatura do pesquisador:

Telefones de contato:
Maternidade Hospital das Clínicas da UFMG – (31) 34099422
Hospital (nome e telefone do hospital colaborador)
Zilma Reis – (31) 985177473 e-mail: skinage.ufmg@gmail.com
Comitê de Ética em Pesquisa da UFMG – Av. Prof. Antônio Carlos, 6627, Unidade Administrativa II, 2° andar, sala 2005, Campus Pampulha, CEP: 31270-901. E-mail:coep@prpq.ufmg.br. Fone (31) 34094592.
Comitê de Ética em Pesquisa do centro colaborador e endereço completo, com e-mail.

Rubrica dos pais: ____________

For peer review only - http://bmjopen.bmj.com/site/about/guidelines.xhtml

Reporting checklist for protocol of a clinical trial.

Based on the SPIRIT guidelines.

Instructions to authors

Complete this checklist by entering the page numbers from your manuscript where readers will find each of the items listed below.

Your article may not currently address all the items on the checklist. Please modify your text to include the missing information. If you are certain that an item does not apply, please write "n/a" and provide a short explanation.

Upload your completed checklist as an extra file when you submit to a journal.

In your methods section, say that you used the SPIRIT reporting guidelines, and cite them as:

Chan A-W, Tetzlaff JM, Altman DG, Laupacis A, Gøtzsche PC, Krleža-Jerić K, Hróbjartsson A, Mann H, Dickersin K, Berlin J, Doré C, Parulekar W, Summerskill W, Groves T, Schulz K, Sox H, Rockhold FW, Rennie D, Moher D. SPIRIT 2013 Statement: Defining standard protocol items for clinical trials. Ann Intern Med. 2013;158(3):200-207

		Reporting Item	Page Number
Title	#1	Descriptive title identifying the study design, population, interventions, and, if applicable, trial acronym	1
Trial registration	#2a	Trial identifier and registry name. If not yet registered, name of intended registry	2
Trial registration: data set	#2b	All items from the World Health Organization Trial Registration Data Set	2
Protocol version	#3	Date and version identifier	6
Funding	#4	Sources and types of financial, material, and other support	18
Roles and responsibilities: contributorship	#5a	Names, affiliations, and roles of protocol contributors	19

For peer review only

Roles and responsibilities: sponsor contact information	#5b	Name and contact information for the trial sponsor	19
Roles and responsibilities: sponsor and funder	#5c	Role of study sponsor and funders, if any, in study design; collection, management, analysis, and interpretation of data; writing of the report; and the decision to submit the report for publication, including whether they will have ultimate authority over any of these activities	18
Roles and responsibilities: committees	#5d	Composition, roles, and responsibilities of the coordinating centre, steering committee, endpoint adjudication committee, data management team, and other individuals or groups overseeing the trial, if applicable (see Item 21a for data monitoring committee)	18
Background and rationale	#6a	Description of research question and justification for undertaking the trial, including summary of relevant studies (published and unpublished) examining benefits and harms for each intervention	4
Background and rationale: choice of comparators	#6b	Explanation for choice of comparators	4
Objectives	#7	Specific objectives or hypotheses	5
Trial design	#8	Description of trial design including type of trial (eg, parallel group, crossover, factorial, single group), allocation ratio, and framework (eg, superiority, equivalence, non-inferiority, exploratory)	6
Study setting	#9	Description of study settings (eg, community clinic, academic hospital) and list of countries where data will be collected. Reference to where list of study sites can be obtained	6
Eligibility criteria	#10	Inclusion and exclusion criteria for participants. If applicable, eligibility criteria for study centres and	7

For peer review only

		individuals who will perform the interventions (eg, surgeons, psychotherapists)	
Interventions: description	#11a	Interventions for each group with sufficient detail to allow replication, including how and when they will be administered	7-8
Interventions: modifications	#11b	Criteria for discontinuing or modifying allocated interventions for a given trial participant (eg, drug dose change in response to harms, participant request, or improving / worsening disease)	9
Interventions: adherance	#11c	Strategies to improve adherence to intervention protocols, and any procedures for monitoring adherence (eg, drug tablet return; laboratory tests)	NA
Interventions: concomitant care	#11d	Relevant concomitant care and interventions that are permitted or prohibited during the trial	9
Outcomes	#12	Primary, secondary, and other outcomes, including the specific measurement variable (eg, systolic blood pressure), analysis metric (eg, change from baseline, final value, time to event), method of aggregation (eg, median, proportion), and time point for each outcome. Explanation of the clinical relevance of chosen efficacy and harm outcomes is strongly recommended	9-10
Participant timeline	#13	Time schedule of enrolment, interventions (including any run-ins and washouts), assessments, and visits for participants. A schematic diagram is highly recommended (see Figure)	11
Sample size	#14	Estimated number of participants needed to achieve study objectives and how it was determined, including clinical and statistical assumptions supporting any sample size calculations	11-12
Recruitment	#15	Strategies for achieving adequate participant enrolment to reach target sample size	7

For peer review only

Allocation: sequence generation	#16a	Method of generating the allocation sequence (eg, computer-generated random numbers), and list of any factors for stratification. To reduce predictability of a random sequence, details of any planned restriction (eg, blocking) should be provided in a separate document that is unavailable to those who enrol participants or assign interventions	NA
Allocation concealment mechanism	#16b	Mechanism of implementing the allocation sequence (eg, central telephone; sequentially numbered, opaque, sealed envelopes), describing any steps to conceal the sequence until interventions are assigned	NA
Allocation: implementation	#16c	Who will generate the allocation sequence, who will enrol participants, and who will assign participants to interventions	NA
Blinding (masking)	#17a	Who will be blinded after assignment to interventions (eg, trial participants, care providers, outcome assessors, data analysts), and how	8
Blinding (masking): emergency unblinding	#17b	If blinded, circumstances under which unblinding is permissible, and procedure for revealing a participant's allocated intervention during the trial	NA
Data collection plan	#18a	Plans for assessment and collection of outcome, baseline, and other trial data, including any related processes to promote data quality (eg, duplicate measurements, training of assessors) and a description of study instruments (eg, questionnaires, laboratory tests) along with their reliability and validity, if known. Reference to where data collection forms can be found, if not in the protocol	12
Data collection plan: retention	#18b	Plans to promote participant retention and complete follow-up, including list of any outcome data to be collected for participants who discontinue or deviate from intervention protocols	12
Data management	#19	Plans for data entry, coding, security, and storage, including any related processes to promote data quality	12

For peer review only

		7(eg, double data entry; range checks for data values). Reference to where details of data management procedures can be found, if not in the protocol	
Statistics: outcomes	#20a	Statistical methods for analysing primary and secondary outcomes. Reference to where other details of the statistical analysis plan can be found, if not in the protocol	12
Statistics: additional analyses	#20b	Methods for any additional analyses (eg, subgroup and adjusted analyses)	12
Statistics: analysis population and missing data	#20c	Definition of analysis population relating to protocol non-adherence (eg, as randomised analysis), and any statistical methods to handle missing data (eg, multiple imputation)	NA
Data monitoring: formal committee	#21a	Composition of data monitoring committee (DMC); summary of its role and reporting structure; statement of whether it is independent from the sponsor and competing interests; and reference to where further details about its charter can be found, if not in the protocol. Alternatively, an explanation of why a DMC is not needed	18
Data monitoring: interim analysis	#21b	Description of any interim analyses and stopping guidelines, including who will have access to these interim results and make the final decision to terminate the trial	NA
Harms	#22	Plans for collecting, assessing, reporting, and managing solicited and spontaneously reported adverse events and other unintended effects of trial interventions or trial conduct	19
Auditing	#23	Frequency and procedures for auditing trial conduct, if any, and whether the process will be independent from investigators and the sponsor	12
Research ethics approval	#24	Plans for seeking research ethics committee / institutional review board (REC / IRB) approval	6

For peer review only

Protocol amendments	#25	Plans for communicating important protocol modifications (eg, changes to eligibility criteria, outcomes, analyses) to relevant parties (eg, investigators, REC / IRBs, trial participants, trial registries, journals, regulators)	19
Consent or assent	#26a	Who will obtain informed consent or assent from potential trial participants or authorised surrogates, and how (see Item 32)	6
Consent or assent: ancillary studies	#26b	Additional consent provisions for collection and use of participant data and biological specimens in ancillary studies, if applicable	NA
Confidentiality	#27	How personal information about potential and enrolled participants will be collected, shared, and maintained in order to protect confidentiality before, during, and after the trial	6
Declaration of interests	#28	Financial and other competing interests for principal investigators for the overall trial and each study site	18
Data access	#29	Statement of who will have access to the final trial dataset, and disclosure of contractual agreements that limit such access for investigators	7
Ancillary and post trial care	#30	Provisions, if any, for ancillary and post-trial care, and for compensation to those who suffer harm from trial participation	NA
Dissemination policy: trial results	#31a	Plans for investigators and sponsor to communicate trial results to participants, healthcare professionals, the public, and other relevant groups (eg, via publication, reporting in results databases, or other data sharing arrangements), including any publication restrictions	7
Dissemination policy: authorship	#31b	Authorship eligibility guidelines and any intended use of professional writers	NA
Dissemination policy: reproducible research	#31c	Plans, if any, for granting public access to the full protocol, participant-level dataset, and statistical code	7

For peer review only

Informed consent materials	#32	Model consent form and other related documentation given to participants and authorised surrogates	6
Biological specimens	#33	Plans for collection, laboratory evaluation, and storage of biological specimens for genetic or molecular analysis in the current trial and for future use in ancillary studies, if applicable	NA

The SPIRIT checklist is distributed under the terms of the Creative Commons Attribution License CC-BY-ND 3.0. This checklist can be completed online using https://www.goodreports.org/, a tool made by the EQUATOR Network in collaboration with Penelope.ai

For peer review only

Prematurity detection evaluating interaction between the skin of the newborn and light: Protocol for the Preemie-Test multicenter clinical trial in Brazilian hospitals to validate a new medical device

Journal:	*BMJ Open*
Manuscript ID	bmjopen-2018-027442.R4
Article Type:	Protocol
Date Submitted by the Author:	16-Jan-2019
Complete List of Authors:	Reis, Zilma; Universidade Federal de Minas Gerais, Gynecology and Obstetrics<br>Guimarães, Rodney; Faculty of Medicine, Universidade Federal de Minas Gerais, Ginecologia e Obstetrícia<br>Rego, Maria Albertina; Faculty of Medicine, Universidade Federal de Minas Gerais, Pediatrics<br>Maia de Castro Romanelli , Roberta ; Universidade Federal de Minas Gerais Faculdade de Medicina,<br>Gaspar, Juliano; Faculty of Medicine, Universidade Federal de Minas Gerais, Obstetrics and Gynecology<br>Vitral, Gabriela; Faculty of Medicine, Universidade Federal de Minas Gerais<br>dos Reis, Marconi; Faculty of Medicine, Universidade Federal de Minas Gerais, Pediatrics<br>Colósimo, Enrico; Universidade Federal de Minas Gerais, Statistics<br>Neves, Gabriela; Hospital Sofia Feldman<br>Vale, Marynea; Hospital Universitario da Universidade Federal do Maranhao, Pediatrics<br>Nader, Paulo; Universidade Luterana do Brasil. Hospital Universitário de Canoas, Pediatrics<br>de Moura, Martha; Hospital Materno Infantil de Brasília<br>de Aguiar, Regina ; Faculty of Medicine, Universidade Federal de Minas Gerais, Obstetrics and Gynecology
**Primary Subject Heading**:	Obstetrics and gynaecology
Secondary Subject Heading:	Global health, Diagnostics, Paediatrics
Keywords:	Gestational Age, Infant, Premature, Skin Physiological Phenomena, Photomedicine, Equipment and Supplies

Prematurity detection evaluating interaction between the skin of the newborn and light: Protocol for the Preemie-Test multicenter clinical trial in Brazilian hospitals to validate a new medical device

Zilma Silveira Nogueira Reis[1]*; Rodney Nascimento Guimarães[1]; Maria Albertina Santiago Rego[1]; Roberta Maia de Castro Romanelli[1]; Juliano de Souza Gaspar[1]; Gabriela Luíza Nogueira Vitral[1]; Marconi Augusto Aguiar dos Reis[1]; Enrico Antônio Colósimo[2]; Gabriela Silveira Neves[3]; Marynea Silva do Vale[4]; Paulo de Jesus Nader[5]; Martha David Rocha de Moura[6]; Regina Amélia Pessoa Lopes de Aguiar[1]

1 - Faculty of Medicine, Universidade Federal de Minas Gerais, Brazil.

2 - Statistics Department, Universidade Federal de Minas Gerais, Brazil.

3 - Hospital Sofia Feldman, Brazil.

4 - Hospital Universitário da Universidade Federal do Maranhão, Brazil.

5 - Universidade Luterana do Brasil. Hospital Universitário de Canoas, Brazil.

6 - Hospital Materno Infantil de Brasília, Brazil.

*Corresponding author: Zilma Silveira Nogueira Reis. Avenida Professor Alfredo Balena, 190, sala 601. Zip Code 30.130.100 Belo Horizonte, Brazil. Phone: +55 0 31 3409-9467. Email: zilma.medicina@gmail.com

For peer review only - http://bmjopen.bmj.com/site/about/guidelines.xhtml

Abstract

Introduction: Recognizing prematurity is critical in order to attend to immediate needs in childbirth settings, guiding the extent of medical care provided for newborns. A new medical device has been developed to carry out the Preemie-Test, an innovative approach to estimate gestational age (GA), based on the photobiological properties of the newborn's skin. This study will validate the Preemie-Test for GA estimation at birth and its accuracy to detect prematurity. Secondarily, the study intends to associate the infant´s skin reflectance with lung maturity, as well as evaluate safety, precision, and usability of a new medical device to offer a suitable product for health professionals during childbirth and in neonatal care settings.

Methods and analysis: Research protocol for diagnosis, single-group, single-blinding, and single-arm multicenter clinical trials with a reference standard. Alive newborns, with 24 weeks or more of pregnancy age, will be enrolled during the first 24 hours of life. Sample size is 787 subjects. The primary outcome is the difference between the GA calculated by the photobiological neonatal skin assessment methodology and the GA calculated by the comparator antenatal ultrasound or reliable last menstrual period. Immediate complications caused by pulmonary immaturity during the first 72 hours of life will be associated with skin reflectance in a nested case-control study.

Ethics and dissemination: Each local independent ethics review board approved the trial protocol. The authors intend to share the minimal anonymized data set necessary to replicate study findings.

Trial registration number: Brazilian Clinical Trials Registry (ReBec) RBR-3f5bm5.

Key-words: Gestational Age, Infant, Premature; Skin Physiological Phenomena; Photomedicine; Equipment and Supplies.

For peer review only - http://bmjopen.bmj.com/site/about/guidelines.xhtml

Article Summary

Strengths and limitations of this study:

- Prospective multicenter evaluation of a new medical device with training, and certification of collaborative centers.
- The gold standard comparator for pregnancy dating does not exist; instead a reference standard will be used with blinded primary outcome.
- The agreement endpoint between methods for gestational age determination precludes randomization of the intervention.

For peer review only

Introduction

In childbirth settings, health professionals continuously need to make timely decisions to provide proper neonatal care. The day of birth is the riskiest for newborns and mothers almost everywhere[1]. Perinatal causes related to prematurity and complications during childbirth, which are generally preventable through qualified health care, are the primary causes of death among newborns[1,2]. Most of these deaths took place in countries with low resources and a scarcity of health facilities[3]. The opportune recognition of prematurity is critical in order to judge the viability of the newborn and to attend to his/her immediate needs, guiding the complexity of the medical care provided for the newborn. Without reliable information on the age of the unborn phase, actions to preserve the potential for survival of the newborn can be neglected[4]. Indeed, the attempted management of the risk of mortality and severe complications are sensitive issues to the gestational age (GA), which involves temperature maintenance, ventilatory support, transport to a neonatal intensive care unit (NICU), and the early treatment of respiratory distress syndrome (RDS), the most severe complication of premature birth[5]. In addition to the GA information or birthweight, the prediction of neonatal respiratory morbidity may be critical in planning immediate medical care[6], since the respiratory system is among the last of the fetal organ systems to mature, which is associated with enhanced morbidity and mortality[6].

Current methods of dating pregnancy remain a worldwide challenge. Early obstetric ultrasound currently offers the best due date[7]. However, access to this type of exam is limited because of high equipment costs, poor training and skills of health professionals, or late prenatal care[8]. Despite a 10-days or more margin of error during the second and third trimester of gestation, ultrasound is still a reasonable methodology for GA determination, when the best opportunity was lost[7]. The calculation, based on the historical information of the last menstrual period (LMP), is

For peer review only - http://bmjopen.bmj.com/site/about/guidelines.xhtml

impacted by the uncertainty of both the fertility days and date of conception[9], due to the bias of memory, the use of hormonal contraception, and breastfeeding[10]. After birth, neurological scores, such as the New Ballard[11], show a tendency to overestimate GA in preterm infants and underestimate GA in growth-restricted infants[12]. Efforts to enhance the reliability of pregnancy dating, through more accurate and accessible technologies, seek to improve pregnancy outcomes and neonatal survival[13].

A new medical device has been developed to carry out the Preemie-Test, an innovative approach used to estimate GA, based on the photobiological properties of the newborn's skin. This reflective test is noninvasive, and the device automatically processes the light, scattered by the constituents of the skin layers, when a small optoelectronic light emitter/receiver sensor touches the newborn’s skin[14]. The device under test is easy to use and every effort is being made to ensure that it has excellent accuracy, be it safe and low cost. The feasibility study provided a mathematical model to predict GA based on the skin reflectance adjusted to clinical variables ($R^2 = 0.828$, $P < 0.001$)[15]. However, before the adoption or use of an innovation, an effectiveness trial of intervention is a critical step in the research chain regarding its the social utility when completing the translation from the proof of concept to clinical science[16]. The rationale for the main hypothesis in this study is that the skin maturity of a newborn, obtained by the analysis of its optical properties, is useful in pregnancy dating for clinical use and respiratory prognosis, especially in a scenario with no reliable GA based on current methods. This study aims to validate the photobiological model of the skin, called the “Preemie-test”, in order to estimate GA at birth and determine its accuracy in detecting prematurity. Secondarily, it also seeks to associate the infant’s skin reflectance with lung maturity. Moreover, this study intends to evaluate the safety, precision, and the usability of a new medical device to offer a suitable product to support health professionals during childbirth and in neonatal care settings.

For peer review only - http://bmjopen.bmj.com/site/about/guidelines.xhtml

Methods

Study design

This study will use a protocol for diagnosis, single-group, single-blinding, and single-arm multicenter clinical trials with a reference standard. This new photobiological approach to the skin, gathered in a medical device, is currently in the pivotal phase of innovation development from the prototype to regulatory approval[17]. This step aims to provide the translation[16] of the scientific model for GA detection based on skin maturity. This Protocol version is 2, January/15th/2019. Faculty of Medicine, Universidade Federal de Minas Gerais is the Coordinator Center.

Study Settings, Ethics and Dissemination

Selected Brazilian referral centers for high-risk pregnancy and neonatal care will participate in the study, according to this protocol: Hospital das Clínicas, Universidade Federal de Minas Gerais, as the Center for Coordination; Hospital Sofia Feldman, Minas Gerais State; Hospital da Universidade Luterana do Brasil, Rio Grande do Sul State; Hospital Materno-infantil de Brasília, Distrito Federal; and Hospital Universitário da Universidade Federal do Maranhão, Maranhão State. Each local independent ethics review board approved the trial protocol, and the Brazilian National Research Council (CONEP) approved all study activities and protocol prior to the commencement of study activities, in accordance with the Declaration of Helsinki (2008), good clinical practice as set forth by the International Organization for Standardization (ISO) 14155:2011, and the Brazilian regulatory health agency's recommendations[18]. This study was logged under both protocol number CAAE 81347817.6.1001.5149 and the International Clinical Trials Registry Platform under number RBR-3f5bm5. Parents will sign an informed consent form on behalf of the newborn before participating in the clinical trial (supplementary file).

For peer review only - http://bmjopen.bmj.com/site/about/guidelines.xhtml

Data Sharing Statement

The authors intend to share the minimal deidentified data set necessary to replicate study findings. Data sharing will include: the reference and comparators GA, GA estimated by the Preemie-test, birth weight, RDS or transient tachypnea of the newborn (TTN) diagnosis, ventilatory support due to pulmonary immaturity, neonatal intensive care unit (NICU) admission due to RDS or TTN, and any adverse events regarding device's safety. Unidentified data and study-related documents as ethical approvals will be accessible by URLs for researchers, regulatory agencies, and sponsors. The correspondent author, orcid.org/0000-0001-6374-9295, will provide data access under reasonable request since the original study citation is warranted.

Patient and Public Involvement

Patients and the public were not involved in the design of this study. The results will be disseminated to study parents of participants through scientific publications, non-scientific publications, and on the website of the project: http://skinage.medicina.ufmg.br.

Eligibility criteria and participant's timeline

A prospective sequential and concurrent enrollment process will select newborns in referral hospitals centers for neonatal care. Infants are eligible with the following inclusion criteria: (1) alive newborn; (2) enrollment during first 24 hours of life; (3) be 24 weeks or more of gestational age, at birth; (4) fetus underwent an obstetric ultrasound assessment before 14 weeks of pregnancy; (5) fetus also had obstetric ultrasound assessment between 14 and 22 gestational weeks. Exclusion criteria are: (1) malformation with structural skin alterations; (2) skin modifiers: anhydramnios, hydrops, congenital skin diseases or chorioamnionitis. Randomisation was not appropriate to assess the agreement between different methods to assess pregnancy dating.

For peer review only - http://bmjopen.bmj.com/site/about/guidelines.xhtml

In a nested case-control study, we will select newborns within the first 72 hours of life, discharge, or death, whichever occurs first, with the following inclusion criteria: (1) RDS or (2) TTN diagnosis. Ranges of gestational age will randomly pair controls. Exclusion criteria include: (1) the existence of extra pulmonary conditions with tachypnea not due to prematurity and (2) diagnosis of Clinical or Laboratory-Confirmed Bloodstream Infection.

Intervention: The Preemie-Test

The Preemie-Test assessment occurs as soon as possible after birth, in the first 24 hours, inside incubators, open heating crib, common crib or in the mother's lap, in order to ensure minimum manipulation and stable clinical conditions. The acquisitions of all newborns will be stored in a database for further statistical analysis.

A noninvasive, handheld optoelectronic prototype has been developed to measure the backscattered light signal from the skin[15]. The equipment regulates the emitted light and processes the received light signal in the sensor, resulting in the prediction of GA by a mathematical model, associated or not with clinical variables. According to the Brazilian regulatory health agency (ANVISA), this medical device is categorized as a Class II safety: noninvasive and medium risk. The prototype unit of measurement and the process of GA estimation were patented under number BR1020170235688 (CTIT-PN862)[14]. An updated version of the invention received improvements in order to safeguard reliability and to minimize examiner interferences on the skin's backscattering acquisition. The light emitting-sensor touches the skin over the sole of the foot for a few seconds. The skin reflectance will be sensed once the light has been emitted by a light emitting diode (LED) at wavelengths from 400 nm to 1200 nm. Data acquisitions occur automatically, without operator influence, and are obtained three times per newborn, in the same site and sequentially. Digital recordings will be

For peer review only - http://bmjopen.bmj.com/site/about/guidelines.xhtml

uploaded to a server for further analysis. The prototype will blind the examiner to the predicted GA value.

The criterium for discontinuing the interventions for a given trial participant will be in case of parents of the newborns' request.

Training and monitoring

Systematic monitoring of data collection, through an electronic information system, would trigger any adverse event. This medical team is still responsible for the training of healthcare professionals to recruit participants, data collection, a safely performed Preemie-Test during the newborn's assessment, and the monitoring of data quality. The certification of co-participant centers involved the accomplishment of at least 30 simulated examinations by the participant health professionals in the study.

Gestational age methods of calculation and comparators

Reference-GA (R) is calculated upon enrollment, using the embryo measurement assessed by ultrasound exam at <14 weeks of gestation as a reference. Crown-rump-length (CRL) data, recorded from the ultrasound report or prenatal care book-document, will be considered the crude data, when available. Intergrowth's 21st standard curve for ultrasound measurements from 7 weeks and 3 days up to 13 weeks and 6 days will be adjusted to all GA data, according to CRL[19].

GA methods to calculate GA in the childbirth setting, and their comparators are as follows:

- Preemie-Test-GA (T): data statistically determined by analyzing the acquired information stored in the device's processor.
- Comparators-GA (C): calculated using the first ultrasound exam after 13 weeks and 6 days of gestation and before 22 weeks (C1). When available, a second

For peer review only - http://bmjopen.bmj.com/site/about/guidelines.xhtml

comparator is GA based on a reliable LMP (C2)[13].

We will take a scanning copy of the prenatal care book or the ultrasound report. After evaluating the data quality, the images will be discarded. To achieve a reliable LMP, we will interview the woman, as suggested by Nguyen et al. (2000)[13].

Primary outcome measures

The primary target is the agreement between the GA offered by the Preemie-Test (T) and the GA calculated by the comparators (C1 and C2), so as to perform the new test in scenarios without the Reference-GA (R). The outcome is the difference between the GA calculated by the photobiological neonatal skin assessment methodology in relation to the age calculated by the comparators.

Another measure for the primary target is the detection of preterm newborns, considering the age before 37 weeks of pregnancy as the threshold between term and preterm births, and analyzing sub-categories of preterm birth, based on GA[4]:

- extremely preterm (less than 28 weeks)
- very preterm (28 to 32 weeks)
- moderate to late preterm (more than 32 to less than 37 weeks).

In this case, the outcome is the proportion of the preterm newborn correctly detected at birth, based on the photobiological test of the skin, within a one-week error.

Secondary outcome measures

1. In a simulated scenario, in which the Reference-GA (R) is unknown, two groups will be randomly assigned from the complete database in order to compare differences among the Reference-GA (R), the GA obtained through the Preemie-Test (T), and the GA calculated by the comparators. Figure 1 presents such subgroups and measures for comparison.

For peer review only - http://bmjopen.bmj.com/site/about/guidelines.xhtml

2. To monitor the device's safety when in regular use by participants over a 72-hour period. Adverse events will be monitored, according to ISO 14155:2011 standards. This means any unexpected medical events, unintended disease or injury, or unfortunate clinical signs in subjects, users, or other people, whether related to the investigational medical device or not.
3. To establish the *ease of use* of the Preemie-Test measurement as a potential method for preterm newborn diagnosis.

The secondary outcome measures in the case-control nested study

Immediate complications, occurring during the first 72 hours of life due to pulmonary immaturity, are the secondary target. The outcome measures are as follows:

- To describe the relationship of the measurement of the newborn's skin reflectance with RDS and with diagnoses based on clinical and radiological findings and respiratory outcomes[6,20].
- To describe the relationship of the measurement of the newborn's skin reflectance with the TTN and with diagnoses based on clinical findings and respiratory outcomes[6].
- To describe the relationship of the measurement of the newborn's skin reflectance with ventilatory support due to pulmonary immaturity.
- To describe the relationship of the measurement of the newborn's skin reflectance with NICU admission due to RDS or TTN.

Time schedule of enrollment, intervention, and outcome measurements are presented in a schematic diagram (see Figure 2). The assessment occurs during the first 24 hours of life, but participants will be followed up for 72 hours or until discharge or death, whichever occurs first, for the monitoring of neonatal outcomes and adverse events.

Sampling and sample size

For peer review only - http://bmjopen.bmj.com/site/about/guidelines.xhtml

The sample size calculation is estimated based on the primary endpoint. To test the hypothesis of equivalence between the Preemie-Test GA and the comparators GA, a sample of 787 subjects is necessary to detect an effect size of 10%. Using the G-Power 3.1 software[21], we assumed an alpha error of 0.05, and a power of test of 0.80 to support a paired t-test.

Sampling intends to arrange three groups of GA enrollment to preserve enough premature newborns with 3:2:1 proportion, similar to Wilson et al. (2017)[22]: 392 term newborns, 263 premature newborns from 32 to 36 weeks and six days of GA, and 132 extremely premature newborns from 24 to 31 weeks and six days of GA.

Usability

The usability assessment will be performed by applying a checklist to participants who use the prototype device to perform the Preemie-Test. The 10 heuristics proposed by Nielsen and Marck (1994)[23] will be adapted to build a checklist to evaluate the device, namely: (a) system visibility, (b) correspondence with the real world, (c) user control and freedom, (d) consistency of results and standardization, (e) error prevention, (f) visual recognition rather than memorization, (g) flexibility and efficiency of use, (h) esthetic and minimalist design, (i) help for the user to recognize, diagnose, and recover from errors, and (j) user documentation and help.

Data collection

Standard operational procedures set data entries in structured questionaries. In this concurrent clinical trial, an electronic information system was developed to collect data in different hospitals, simultaneously. Entry forms validations were implemented with data values ranges to ensure the quality of the information. An audit of the data will be permanently performed and the data summary available on the project webpage. Double system, paper-based and electronic will permit audit concerning reliability and

For peer review only - http://bmjopen.bmj.com/site/about/guidelines.xhtml

validity. Independent rater over-read all papers files and cross check with the electronic information from all patients.

Data analysis

Demographics and baseline characteristics of the study group, as well the intervention measurements, will be summarized by the frequencies and the mean and standard deviation (SD), the whereas median and interquartile range will be preferred for non-normally distributed continuous variables.

To model the GA prediction, computational randomization will select two subsamples in the database. One of them to train the prediction model of GA based on skin reflectance and clinical variables, such as sex, time in an incubator, phototherapy, birth weight, among others. Another part will be for the analytical validation of the predictive model. Improvements in the existing prediction models for GA (Preemie-Test), will be conducted with conventional statistical and data mining analyses.

Regarding the primary endpoint, the agreement among three methods for GA will be calculated using the Intraclass coefficient correlation and Bland & Altman plots[24], and paired t-testing. The accuracy of the Preemie-Test in identifying the premature newborn, within a one-week margin of error, will be the target of the accuracy analysis.

The relationship between the measurement of the newborn's skin reflectance and complications due to pulmonary distress associated with immaturity will be evaluated by means of association tests and risk. The significance level for hypothesis tests will be 5%, together with 95% confidence intervals.

Discussion

Strengths and Limitations

For peer review only - http://bmjopen.bmj.com/site/about/guidelines.xhtml

Availability of trustworthy GA information is a prerequisite for preterm birth classification and healthcare decisions[25]. In this light, the results of this clinical study have the potential to validate a new device for pregnancy dating. The Preemie-Test was prepared to operate with minimum operator intervention and for use by healthcare professionals anywhere a birth takes place without a reliable GA.

The purpose of medical research involving neonates is intended to improve clinical procedures[26]. In this context, a clinical trial is a research study in which subjects are prospectively assigned to intervention and the effects of those interventions on health-related outcomes are thereby evaluated[27]. However, clinical trials on medical devices face barriers when an effective standard procedure does not exist, as is the case of the comparator procedure[28]. Our challenge in preparing the present protocol was the absence of a gold standard for pregnancy dating, since the fetal age begins upon conception; however, this information is difficult to be accurately determined[7].

The study begun with the training of health professionals in September 2018.

Planned Date of First Enrollment: 2019-01-02.

Planned Date of Last Enrollment: 2019-12-31.

Data analysis will be finalized, the results of which are expected in May 2020.

References

1. Children St. Surviving the first day. In: *State of the world's mothers 2013.* Vol 1. Connecticut: Save the Children; 2013: http://www.savethechildren.org/atf/cf/%7B9def2ebe-10ae-432c-9bd0-df91d2eba74a%7D/SOWM-FULL-REPORT_2013.PDF.

For peer review only - http://bmjopen.bmj.com/site/about/guidelines.xhtml

2. França EB, Lansky S, Rego MAS, et al. Principais causas da mortalidade na infância no Brasil, em 1990 e 2015: estimativas do estudo de Carga Global de Doença. *Revista Brasileira de Epidemiologia.* 2017;20:46-60.

3. Federation StC. HNN website, Healthy Newborn Network. https://www.healthynewbornnetwork.org/. Published 2018. Accessed 01/17/2018, 2018.

4. Howson CP, Kinney MV, McDougall L, Lawn JE, Group BTSPBA. Born too soon: preterm birth matters. *Reprod Health.* 2013;10 Suppl 1:S1.

5. Bhutta ZA, Giuliani F, Haroon A, et al. Standardisation of neonatal clinical practice. *BJOG.* 2013;120 Suppl 2:56-63, v.

6. Liszewski MC, Stanescu AL, Phillips GS, Lee EY. Respiratory Distress in Neonates: Underlying Causes and Current Imaging Assessment. *Radiol Clin North Am.* 2017;55(4):629-644.

7. Committee on Obstetric Practice tAIoUiM, and the Society for Maternal-Fetal Medicine. Committee Opinion No 700: Methods for Estimating the Due Date. *Obstet Gynecol.* 2017;129(5):e150-e154.

8. Karl S, Li Wai Suen CS, Unger HW, et al. Preterm or not--an evaluation of estimates of gestational age in a cohort of women from Rural Papua New Guinea. *PLoS One.* 2015;10(5):e0124286.

9. Wilcox AJ, Weinberg CR, Baird DD. Timing of Sexual Intercourse in Relation to Ovulation — Effects on the Probability of Conception, Survival of the Pregnancy, and Sex of the Baby. *New England Journal of Medicine.* 1995;333(23):1517-1521.

For peer review only - http://bmjopen.bmj.com/site/about/guidelines.xhtml

For peer review only

10. Lynch CD, Zhang J. The research implications of the selection of a gestational age estimation method. *Paediatr Perinat Epidemiol.* 2007;21 Suppl 2:86-96.

11. Ballard JL, Khoury JC, Wedig K, Wang L, Eilers-Walsman BL, Lipp R. New Ballard Score, expanded to include extremely premature infants. *The Journal of Pediatrics.* 1991;119(3):417-423.

12. Lee AC, Panchal P, Folger L, et al. Diagnostic Accuracy of Neonatal Assessment for Gestational Age Determination: A Systematic Review. *Pediatrics.* 2017;140(6).

13. Nguyen TH, Larsen T, Engholm G, Møller H. Increased adverse pregnancy outcomes with unreliable last menstruation. *Obstet Gynecol.* 2000;95(6 Pt 1):867-873.

14. Reis ZSN, Guimarães RN, Inventors. Dispositivo para determinação da idade gestacional, processos e usos. Nov/1/2016, 2016.

15. Reis ZSN, Simeoni U, Vitral GLN, Souza IMFd, Rego MAS, Guimaraes RN. Newborn skin reflection: Proof of concept for a new approach for predicting gestational age at birth. A cross-sectional study. *Plos One.* 2017.

16. Reis ZSN, Vitral GLN, de Souza IMF, Rego MAS, Guimaraes RN. Newborn skin reflection: Proof of concept for a new approach for predicting gestational age at birth. A cross-sectional study. *PLOS ONE.* 2017;12(9):e0184734.

17. Kaplan AV, Baim DS, Smith JJ, et al. Medical device development: from prototype to regulatory approval. *Circulation.* 2004;109(25):3068-3072.

18. Brasil. Resolução da Diretoria Colegiada - RDC No. 10. In: Sanitária AnNdVn, ed. Vol 10. Brasilia: Diário Oficial da União, de 03 de março de 2015; 2015.

For peer review only

For peer review only - http://bmjopen.bmj.com/site/about/guidelines.xhtml

19. Papageorghiou AT, Kennedy SH, Salomon LJ, et al. International standards for early fetal size and pregnancy dating based on ultrasound measurement of crown-rump length in the first trimester of pregnancy. *Ultrasound Obstet Gynecol.* 2014;44(6):641-648.

20. Reuter S, Moser C, Baack M. Respiratory distress in the newborn. *Pediatr Rev.* 2014;35(10):417-428; quiz 429.

21. Faul F, Erdfelder E, Lang A-G, Buchner A. G* Power 3: A flexible statistical power analysis program for the social, behavioral, and biomedical sciences. *Behavior research methods.* 2007;39(2):175-191.

22. Wilson K, Hawken S, Murphy MS, et al. Postnatal Prediction of Gestational Age Using Newborn Fetal Hemoglobin Levels. *EBioMedicine.* 2017;15:203-209.

23. Nielsen J. Usability inspection methods. Conference Companion on Human Factors in Computing Systems; 1994; Boston, Massachusetts, USA.

24. Altman DG BJ. Measurement in medicine: the analysis of method comparison studies. 1983:307-317

25. Beydoun H, Ugwu B, Oehninger S. Assisted reproduction for the validation of gestational age assessment methods. *Reproductive biomedicine online.* 2011;22(4):321-326.

26. WHO. Clinical Trials in Children. Essential medicines for children. World Health Organisation. http://www.who.int/ictrp/child/en/. Accessed 08/01/2018, 2018.

27. USA. NIH's Definition of clinical trial. In: Services USDoHaH, ed. Bethesda: National Institutes of Health; 2018.

For peer review only - http://bmjopen.bmj.com/site/about/guidelines.xhtml

28. Neugebauer EAM, Rath A, Antoine SL, et al. Specific barriers to the conduct of randomised clinical trials on medical devices. *Trials.* 2017;18(1):427.

Author statement:

ZSNR: designed the study, planned data collection, prepared the team for good clinical practices, wrote and revised the paper. RNG, RAPLA, MASR, RMCR and JSG made substantial contributions to study design, planned data collection, prepared the team for good clinical practices, wrote and revised the paper. GLNV, MAAR, GSN, PJN, MDRM, and MSV made contributions to standard procedures in methods, drafted the manuscript, reviewed the paper, and approved the final manuscript. EAC: drafted the work and reviewed it critically for important intellectual content, as statistic consultant.

Funding statement

This research was supported by the Grand Challenges Exploration from the Bill & Melinda Gates Foundation (Grant number OPP1128907, Contract), http://www.gatesfoundation.org/, and Fundação de Amparo a Pesquisa de Minas Gerais, Brazil, http://www.fapemig.br/en/, non-profit sectors. The clinical trial is funded by the Brazilian Ministry of Health, Program of Development of the Industrial Health Complex (PROCIS), project 23072.052747/2017-51, trial sponsor contact: Fotini Toscas, e-mail fotini.toscas@saude.gov.br. The funders played no role in the study design, data collection and analysis, decision to publish, or preparation of the manuscript.

For peer review only - http://bmjopen.bmj.com/site/about/guidelines.xhtml

Acknowledgements

The authors would like to thank Center for Clinical Research of Hospital das Clínicas, Universidade Federal de Minas Gerais for general support and Good clinical practices training; Fotini Toscas from the Brazilian Ministry of Health for the active intermediation as the trial sponsor contact.

Roles and responsibilities: ZSNR is the Principal Investigator and coordinator of the Directive Committee. JSG is the coordinator of the Data Management Team and will continuously receive report adverse events of trial interventions or trial conduct. RAPLA is the coordinator of the Clinical Trial Quality Committee, responsible for important protocol modifications, if necessary.

Conflict of interests statement

Authors declare a patent deposit on behalf of the Universidade Federal de Minas Gerais and Fundação de Amparo a Pesquisa de Minas Gerais, Brazil, http://www.fapemig.br/en/. The inventors were Reis, Zilma Silveira Nogueira and Guimaraes, Rodney Nascimento: BR1020170235688 (CTIT-PN862).

For peer review only

Figure 1. Secondary outcome comparisons between the reference GA and the Preemie-Test in a simulated scenario without best pregnancy dating

Legends: *Gestational age from crown-rump-length data adjusted to Intergrowth's 21st fetal standard[19]. R: reference. GA: gestational age. T: test. C1: comparator 1 is the gestational age calculated using the first ultrasound

For peer review only - http://bmjopen.bmj.com/site/about/guidelines.xhtml

exam after 13 weeks and 6 days and before 22 weeks of gestation. C2: comparator 2 is the gestational age based on a reliable last menstrual period.

Figure 2. Participant timeline of the study

Legends: GA: gestational age. R: reference. LMP: last menstrual period.

For peer review only

Word Count: 3415 words

For peer review only - http://bmjopen.bmj.com/site/about/guidelines.xhtml

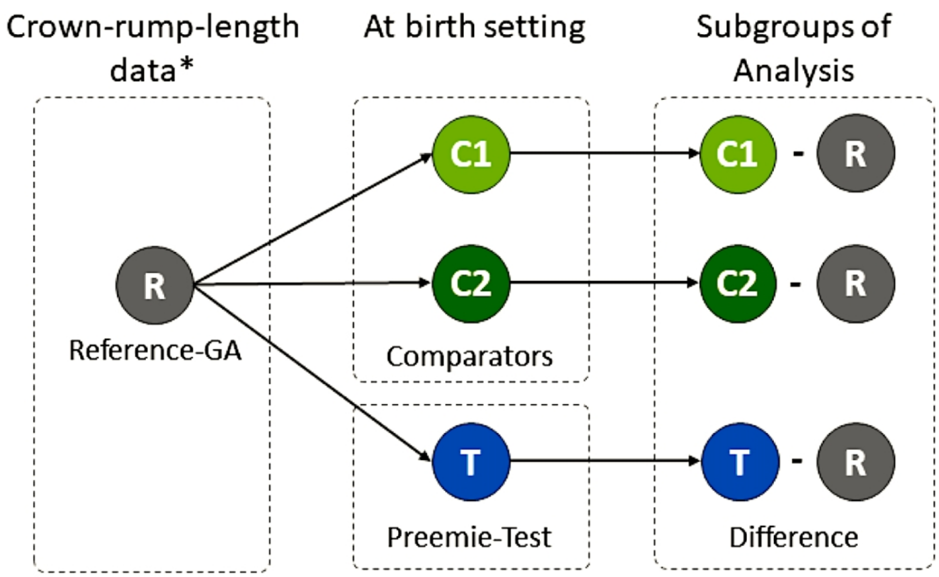

Secondary outcome comparisons between the reference GA and the Preemie-Test in a simulated scenario without best pregnancy dating

Legends: *Gestational age from crown-rump-length data adjusted to Intergrowth's 21st fetal standard19. R: reference. GA: gestational age. T: test. C1: comparator 1 is the gestational age calculated using the first ultrasound exam after 13 weeks and 6 days and before 22 weeks of gestation. C2: comparator 2 is the gestational age based on a reliable last menstrual period.

143x90mm (300 x 300 DPI)

	STUDY PERIOD			
	Enrollment	Assessment	Close-out	Allocation
TIMEPOINT	0	0	72 hours	Analysis
**ENROLLMENT:**				
Eligibility screen	X			
Informed consent	X			
**INTERVENTION:**				
Preemie-Test		X		
**ASSESSMENTS AND ANALYSIS:**				
Preemie-Test: data acquisition		X		
Reference GA: calculated by obstetric ultrasound at <14 weeks of gestation	X			X
Comparator 1: GA calculated by obstetric ultrasound at ≥ 14 and <22 weeks	X			X
Comparator 2: GA calculated by reliable LMP	X			X
Case-control nested study: lung maturity		◆———	———◆	

Participant timeline of the study

Legends: GA: gestational age. R: reference. LMP: last menstrual period.

157x123mm (300 x 300 DPI)

TERMO DE CONSENTIMENTO LIVRE E ESCLARECIDO

Detecção da prematuridade através da interação entre a luz e a pele neonatal: a validação do Preemie-Teste

Sob responsabilidade da pesquisadora Profa Zilma Silveira Nogueira Reis

Cara senhora, você está sendo convidada a participar deste estudo porque acaba de ter um parto no hospital (nome do hospital do centro colaborador) ____.

Apresentação do estudo

O objetivo deste estudo é descobrir novas técnicas para estimar a idade de um bebê ao nascer e identificar aqueles que nasceram antes de nove meses, os prematuros. A idade gestacional desconhecida pode aumentar o risco dos bebês no momento de seu nascimento. As técnicas atuais para se estimar a idade do bebê possuem grande margem de erro.

Acreditamos que a pele possui características que, se bem estudadas, podem refletir a idade das pessoas, e também dos bebês. Por isso, estamos desenvolvendo um novo equipamento médico que se encontra em teste. Ele utiliza a luz para avaliar a composição da pele do bebê e detectar sua idade. Os resultados poderão beneficiar os bebês que nascem sem a informação confiável da idade gestacional.

Instituições envolvidas no estudo

O estudo é desenvolvido pela Faculdade de Medicina da Universidade Federal de Minas Gerais (UFMG), em cooperação com maternidades brasileiras, entre elas a que você se encontra internada. A previsão deste estudo é que 787 crianças recém-nascidas sejam examinadas.

A participação no estudo, riscos e cuidados

Convidamos você e seu bebê para participar deste estudo. Isso incluirá um exame na pele do bebê com a luz, uma breve entrevista com você e a consulta aos registros de saúde sobre a gravidez e os do seu bebê neste hospital. Na entrevista serão tomados todos os cuidados a fim de minimizar os constrangimentos para você. A consulta ao prontuário médico será realizada resguardando o direito de sigilo da informação. Pedimos sua permissão para fotografar a caderneta da gestante ou outro documento equivalente, para conferir a idade gestacional calculada pelos ciclos menstruais e pelos exames de ultrassom. As partes da fotografia que contenham sua identificação serão retiradas da imagem e a manteremos até o final do estudo, quando o arquivo será apagado dos registros da pesquisa.

Pedimos sua permissão para fazer um exame na pele de seu bebê, na região da sola do pé, usando um equipamento em teste. O exame é indolor e externo ao corpo, considerado não-invasivo. A parte que encosta no bebê é pequena e não apresenta pontas que possam ferir a sua pele. Outros equipamentos parecidos, que emitem luz, já são usados nos bebês de forma segura. Por exemplo o oxímetro que faz teste do coraçãozinho. Assim como esse, não se espera que ocorram efeitos imediatos ou futuros na saúde do bebê. Os riscos do teste que faremos incluem a exposição do pé do bebê com perda temporária de calor do corpo e estresse. Cuidados serão tomados a fim de minimizar estes desconfortos. Esclarecemos que o teste dura alguns segundos reduzindo ao mínimo chance de causar marcas ou irritação no local. Caso seu bebê apresente sinais de desconforto durante o exame, o mesmo será interrompido. Você ou familiares poderão permanecer junto ao seu filho durante o exame. Nas crianças que estiverem na Unidade Neonatal, o exame será realizado onde ela já está sendo cuidada, acompanhado pelo profissional de saúde que já está cuidando dela. Caso o seu bebê seja prematuro, todos os devidos cuidados serão tomados antes de cada exame para reduzir a chance de perda de calor, seguindo todas as recomendações de um bebê que fica em incubadora.

Esclarecemos que este estudo não trará benefícios diretos a você ou seu filho, entretanto auxiliará na validação de um novo teste que poderá no futuro identificar o bebê prematuro. Os resultados poderão também gerar informações que ajudem a melhorar os cuidados com outros bebês, quando a idade gestacional é desconhecida. Informamos que os resultados da pesquisa serão publicados em revistas científicas e apresentados em congressos, sem contudo revelar sua identidade ou a do bebê.

For peer review only - http://bmjopen.bmj.com/site/about/guidelines.xhtml

As informações obtidas durante a pesquisa serão confidenciais, guardadas em computadores, protegidos por senha e não serão usadas para outros fins. O roubo das informações que coletaremos no estudo é um risco remoto. Para isso, as melhores práticas em segurança de dados serão empregadas. Também poderão ter acesso aos dados da pesquisa o comitê que coordena o estudo, assim como a agência reguladora ANVISA, sem jamais violar a confidencialidade e privacidade dos dados, para que seja possível monitorar se os procedimentos de qualidade e segurança da pesquisa estão sendo seguidos.

Seus direitos como participante

Informamos que a sua participação deve ser voluntária, ou seja, não é obrigatória e caso não concorde ou resolva desistir a qualquer momento isto não trará nenhum constrangimento para você ou para a forma como você será tratada neste hospital. Também não está previsto nenhum tipo de pagamento por sua participação na pesquisa. Este estudo não implica em gastos para você, pois não terá que se deslocar para outro local, permanecer mais tempo no hospital, uma vez que o exame é feito durante sua internação e de seu bebê na maternidade. Caso seja de seu interesse, os resultados do exame que estarão guardados com o pesquisador e lhe serão entregues assim que você solicitar.

Os pesquisadores garantem que acompanharão gratuitamente seu bebê durante a realização do exame e a qualquer momento que se fizer necessário, em qualquer problema que por ventura esteja associado ao estudo ou efeito do teste com a luz.

Este Termo de Consentimento está elaborado em duas vias iguais. Ambas devem ser assinadas por você, pelo pai da criança e pelo pesquisador. Uma via ficará com o participante e a outra com o pesquisador.

O Comitê de Ética em Pesquisa da UFMG pode ser contatado em caso de haver dúvidas quanto aos aspectos éticos da pesquisa, através do telefone (31) 3409-4592 ou endereço completo apresentado a seguir.

Meu nome	
Documento de identidade	
Data de hoje	

Eu declaro que estou em condições de tomar esta decisão e ciente do que foi exposto acima. Autorizo o uso de minhas informações de saúde e as do meu bebê para este projeto de pesquisa, assim como a realização do novo teste. Participo voluntariamente deste estudo e estou ciente que o exame na pele do meu bebê com a luz não traz prejuízo à sua saúde

Assinatura da puérpera:
Assinatura do pai da criança:
Assinatura do pesquisador:

Telefones de contato:
Maternidade Hospital das Clínicas da UFMG – (31) 34099422
Hospital (nome e telefone do hospital colaborador)
Zilma Reis – (31) 985177473 e-mail: skinage.ufmg@gmail.com
Comitê de Ética em Pesquisa da UFMG – Av. Prof. Antônio Carlos, 6627, Unidade Administrativa II, 2° andar, sala 2005, Campus Pampulha, CEP: 31270-901. E-mail:coep@prpq.ufmg.br. Fone (31) 34094592.
Comitê de Ética em Pesquisa do centro colaborador e endereço completo, com e-mail.

For peer review only - http://bmjopen.bmj.com/site/about/guidelines.xhtml

Reporting checklist for protocol of a clinical trial.

Based on the SPIRIT guidelines.

Instructions to authors

Complete this checklist by entering the page numbers from your manuscript where readers will find each of the items listed below.

Your article may not currently address all the items on the checklist. Please modify your text to include the missing information. If you are certain that an item does not apply, please write "n/a" and provide a short explanation.

Upload your completed checklist as an extra file when you submit to a journal.

In your methods section, say that you used the SPIRIT reporting guidelines, and cite them as:

Chan A-W, Tetzlaff JM, Altman DG, Laupacis A, Gøtzsche PC, Krleža-Jerić K, Hróbjartsson A, Mann H, Dickersin K, Berlin J, Doré C, Parulekar W, Summerskill W, Groves T, Schulz K, Sox H, Rockhold FW, Rennie D, Moher D. SPIRIT 2013 Statement: Defining standard protocol items for clinical trials. Ann Intern Med. 2013;158(3):200-207

		Reporting Item	Page Number
Title	#1	Descriptive title identifying the study design, population, interventions, and, if applicable, trial acronym	1
Trial registration	#2a	Trial identifier and registry name. If not yet registered, name of intended registry	2
Trial registration: data set	#2b	All items from the World Health Organization Trial Registration Data Set	2
Protocol version	#3	Date and version identifier	6
Funding	#4	Sources and types of financial, material, and other support	18
Roles and responsibilities: contributorship	#5a	Names, affiliations, and roles of protocol contributors	19

For peer review only

Roles and responsibilities: sponsor contact information	#5b	Name and contact information for the trial sponsor	19
Roles and responsibilities: sponsor and funder	#5c	Role of study sponsor and funders, if any, in study design; collection, management, analysis, and interpretation of data; writing of the report; and the decision to submit the report for publication, including whether they will have ultimate authority over any of these activities	18
Roles and responsibilities: committees	#5d	Composition, roles, and responsibilities of the coordinating centre, steering committee, endpoint adjudication committee, data management team, and other individuals or groups overseeing the trial, if applicable (see Item 21a for data monitoring committee)	18
Background and rationale	#6a	Description of research question and justification for undertaking the trial, including summary of relevant studies (published and unpublished) examining benefits and harms for each intervention	4
Background and rationale: choice of comparators	#6b	Explanation for choice of comparators	4
Objectives	#7	Specific objectives or hypotheses	5
Trial design	#8	Description of trial design including type of trial (eg, parallel group, crossover, factorial, single group), allocation ratio, and framework (eg, superiority, equivalence, non-inferiority, exploratory)	6
Study setting	#9	Description of study settings (eg, community clinic, academic hospital) and list of countries where data will be collected. Reference to where list of study sites can be obtained	6
Eligibility criteria	#10	Inclusion and exclusion criteria for participants. If applicable, eligibility criteria for study centres and	7

For peer review only

		individuals who will perform the interventions (eg, surgeons, psychotherapists)	
Interventions: description	#11a	Interventions for each group with sufficient detail to allow replication, including how and when they will be administered	7-8
Interventions: modifications	#11b	Criteria for discontinuing or modifying allocated interventions for a given trial participant (eg, drug dose change in response to harms, participant request, or improving / worsening disease)	9
Interventions: adherance	#11c	Strategies to improve adherence to intervention protocols, and any procedures for monitoring adherence (eg, drug tablet return; laboratory tests)	NA
Interventions: concomitant care	#11d	Relevant concomitant care and interventions that are permitted or prohibited during the trial	9
Outcomes	#12	Primary, secondary, and other outcomes, including the specific measurement variable (eg, systolic blood pressure), analysis metric (eg, change from baseline, final value, time to event), method of aggregation (eg, median, proportion), and time point for each outcome. Explanation of the clinical relevance of chosen efficacy and harm outcomes is strongly recommended	9-10
Participant timeline	#13	Time schedule of enrolment, interventions (including any run-ins and washouts), assessments, and visits for participants. A schematic diagram is highly recommended (see Figure)	11
Sample size	#14	Estimated number of participants needed to achieve study objectives and how it was determined, including clinical and statistical assumptions supporting any sample size calculations	11-12
Recruitment	#15	Strategies for achieving adequate participant enrolment to reach target sample size	7

For peer review only

Allocation: sequence generation	#16a	Method of generating the allocation sequence (eg, computer-generated random numbers), and list of any factors for stratification. To reduce predictability of a random sequence, details of any planned restriction (eg, blocking) should be provided in a separate document that is unavailable to those who enrol participants or assign interventions	NA
Allocation concealment mechanism	#16b	Mechanism of implementing the allocation sequence (eg, central telephone; sequentially numbered, opaque, sealed envelopes), describing any steps to conceal the sequence until interventions are assigned	NA
Allocation: implementation	#16c	Who will generate the allocation sequence, who will enrol participants, and who will assign participants to interventions	NA
Blinding (masking)	#17a	Who will be blinded after assignment to interventions (eg, trial participants, care providers, outcome assessors, data analysts), and how	8
Blinding (masking): emergency unblinding	#17b	If blinded, circumstances under which unblinding is permissible, and procedure for revealing a participant's allocated intervention during the trial	NA
Data collection plan	#18a	Plans for assessment and collection of outcome, baseline, and other trial data, including any related processes to promote data quality (eg, duplicate measurements, training of assessors) and a description of study instruments (eg, questionnaires, laboratory tests) along with their reliability and validity, if known. Reference to where data collection forms can be found, if not in the protocol	12
Data collection plan: retention	#18b	Plans to promote participant retention and complete follow-up, including list of any outcome data to be collected for participants who discontinue or deviate from intervention protocols	12
Data management	#19	Plans for data entry, coding, security, and storage, including any related processes to promote data quality	12

For peer review only

		7(eg, double data entry; range checks for data values). Reference to where details of data management procedures can be found, if not in the protocol	
Statistics: outcomes	#20a	Statistical methods for analysing primary and secondary outcomes. Reference to where other details of the statistical analysis plan can be found, if not in the protocol	12
Statistics: additional analyses	#20b	Methods for any additional analyses (eg, subgroup and adjusted analyses)	12
Statistics: analysis population and missing data	#20c	Definition of analysis population relating to protocol non-adherence (eg, as randomised analysis), and any statistical methods to handle missing data (eg, multiple imputation)	NA
Data monitoring: formal committee	#21a	Composition of data monitoring committee (DMC); summary of its role and reporting structure; statement of whether it is independent from the sponsor and competing interests; and reference to where further details about its charter can be found, if not in the protocol. Alternatively, an explanation of why a DMC is not needed	18
Data monitoring: interim analysis	#21b	Description of any interim analyses and stopping guidelines, including who will have access to these interim results and make the final decision to terminate the trial	NA
Harms	#22	Plans for collecting, assessing, reporting, and managing solicited and spontaneously reported adverse events and other unintended effects of trial interventions or trial conduct	19
Auditing	#23	Frequency and procedures for auditing trial conduct, if any, and whether the process will be independent from investigators and the sponsor	12
Research ethics approval	#24	Plans for seeking research ethics committee / institutional review board (REC / IRB) approval	6

For peer review only

Protocol amendments	#25	Plans for communicating important protocol modifications (eg, changes to eligibility criteria, outcomes, analyses) to relevant parties (eg, investigators, REC / IRBs, trial participants, trial registries, journals, regulators)	19
Consent or assent	#26a	Who will obtain informed consent or assent from potential trial participants or authorised surrogates, and how (see Item 32)	6
Consent or assent: ancillary studies	#26b	Additional consent provisions for collection and use of participant data and biological specimens in ancillary studies, if applicable	NA
Confidentiality	#27	How personal information about potential and enrolled participants will be collected, shared, and maintained in order to protect confidentiality before, during, and after the trial	6
Declaration of interests	#28	Financial and other competing interests for principal investigators for the overall trial and each study site	18
Data access	#29	Statement of who will have access to the final trial dataset, and disclosure of contractual agreements that limit such access for investigators	7
Ancillary and post trial care	#30	Provisions, if any, for ancillary and post-trial care, and for compensation to those who suffer harm from trial participation	NA
Dissemination policy: trial results	#31a	Plans for investigators and sponsor to communicate trial results to participants, healthcare professionals, the public, and other relevant groups (eg, via publication, reporting in results databases, or other data sharing arrangements), including any publication restrictions	7
Dissemination policy: authorship	#31b	Authorship eligibility guidelines and any intended use of professional writers	NA
Dissemination policy: reproducible research	#31c	Plans, if any, for granting public access to the full protocol, participant-level dataset, and statistical code	7

For peer review only

Informed consent materials	#32	Model consent form and other related documentation given to participants and authorised surrogates	6
Biological specimens	#33	Plans for collection, laboratory evaluation, and storage of biological specimens for genetic or molecular analysis in the current trial and for future use in ancillary studies, if applicable	NA

The SPIRIT checklist is distributed under the terms of the Creative Commons Attribution License CC-BY-ND 3.0. This checklist can be completed online using https://www.goodreports.org/, a tool made by the EQUATOR Network in collaboration with Penelope.ai

For peer review only
